# Reductive site-selective atypical *C*,*Z*-type/N2-C2 cleavage allows C-terminal protein amidation

**DOI:** 10.1126/sciadv.abl8675

**Published:** 2022-04-08

**Authors:** Tim A. Mollner, Andrew M. Giltrap, Yibo Zeng, Yana Demyanenko, Charles Buchanan, Daniel Oehlrich, Andrew J. Baldwin, Daniel C. Anthony, Shabaz Mohammed, Benjamin G. Davis

**Affiliations:** 1 Department of Chemistry, Chemistry Research Laboratory, University of Oxford, Oxford, UK.; 2 The Rosalind Franklin Institute, Oxfordshire, UK.; 3 Global Medicinal Chemistry, Janssen Research & Development, Beerse, Belgium.; 4 Department of Pharmacology, University of Oxford, Oxford, UK.; 5 Department of Biochemistry, University of Oxford, Oxford, UK.

## Abstract

Biomolecule environments can enhance chemistries with the potential to mediate and modulate self-modification (e.g., self-cleavage). While these enhanced modes are found in certain biomolecules (e.g., RNA ribozymes), it is more rare in proteins. Targeted proteolytic cleavage is vital to physiology, biotechnology, and even emerging therapy. Yet, purely chemically induced methods for the site-selective cleavage of proteins remain scarce. Here, as a proof of principle, we designed and tested a system intended to combine protein-enhanced chemistry with tag modification to enable synthetic reductive protein chemistries promoted by diboron. This reductively driven, single-electron chemistry now enables an operationally simple, site-selective cleavage protocol for proteins directed to readily accessible dehydroalanine (Dha) residues as tags under aqueous conditions and in cell lysates. In this way, a mild, efficient, enzyme-free method now allows not only precise chemical proteolysis but also simultaneous use in the removal of affinity tags and/or protein-terminus editing to create altered N- and C-termini such as protein amidation (─CONH_2_).

## Teaser

A biomimetic reductive reagent cleaves proteins at a nonpeptidic bond to leave amide termini that can switch function.

## INTRODUCTION

In several biomolecules, environments exist suitable for activating chemistry. In some specific cases, such as RNA ribozymes (*1*), diverse binding (“sampling”) of (co)reagents at many sites enables highly selective self-modifying chemistries, including self-cleavage. In this way, for example, divalent metals can bind RNA at many possible sites but may end up driving hydrolytic, self-cleavage chemistry in ribozymes at just one (*2*).

**Fig. 1.**
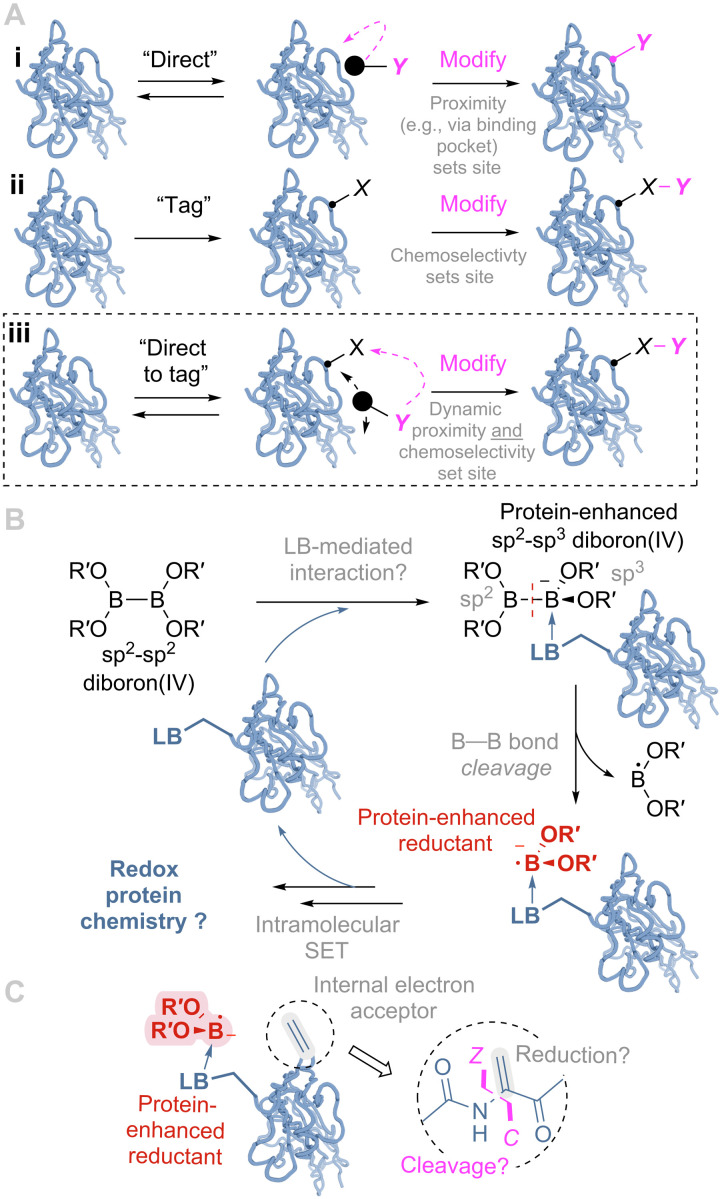
Design strategies for site-selective protein modification. (**A**) (i) Ligand directed, (ii) tag and modify, and (iii) design hypotheses that informed this work: Dynamic, reversible sampling of a directed reagent might drive enhanced chemistries via direct-to-tag selectivity in proteins. (**B**) Potential reductive direct-to-tag chemistry using diboron(4) compounds as possible dynamic, protein-enhanced reagents. (**C**) Possible differing reductive activity that would be directed to Dha using this method. LB, Lewis basic moiety. SET, single electron transfer.

In proteins, approaches using ligands to recruit a reactive moiety to a given, predetermined site for chemical protein modification have attracted increasing attention during the past decade (*3*, *4*). This method exploits the regioselectivity of a ligand to direct location (Fig. 1Ai). Other complementary approaches reliant upon the location of a preinserted functional group (or “tag”) (*5*, *6*) exploit instead chemoselectivity to direct location (Fig. 1Aii). However, we speculated that the exploitation of ligand direction in combination with chemoselectivity could provide additional reaction manifolds that would merge both (“direct-to-tag”; Fig. 1Aiii) in a manner that might exploit sampling similar to that seen in, e.g., ribozymes.

Boron sits at a dynamic, cusp between Lewis acidic sp^2^ 6e^−^ and liganded sp^3^ 8e^−^ states. Notably, its liganded state can be accomplished even in competing Lewis basic solvents such as water. Elegant prior methods have exploited the selective engagement of boron-containing ligands with Lewis bases (LBs) in predetermined regions of proteins, such as boron-containing inhibitors in enzyme active sites (*7*) or *bis-*boronates that target peptidic sequences (*8*). This localized binding might therefore, on initial inspection, prove too restrictive to an intended direct-to-tag manifold (Fig. 1Aiii). However, we have recently shown (through post-translational insertion of the minimal borono residue, boronoalanine, into proteins) that small boronyl moieties can transiently and dynamically sample protein surfaces via carbonyl backbone engagement in a broad manner (*9*). This therefore suggested a design in which boronyls R–B(OR′)_2_ might act as reagents whose reactivity could be enhanced by more complex protein environments.

The transformations achievable with boron reagents, and diborons specifically, have recently been shown to extend far beyond more classical borylation and transmetallation reactions, including various reductions, in situ hydrogen generation, and radical initiation (*10*–*17*). Diboron(4) compounds [R = B(OR′)_2_] are therefore reagents with diverse and flexible reactivity. Moreover, when liganded by LBs from a “resting” sp^2^-sp^2^ state into an sp^2^-sp^3^ (or even sp^3^-sp^3^) (*18*) state, reductive manifolds are activated (*10*). This could therefore allow protein environments to activate or enhance reductive states in diboron(4) (Fig. 1B).

We have previously identified low-level reduction-driven side reactions of dehydroalanine (Dha) that lead to either terminal reduction (to Ala) or subsequent oxidative cleavage following trapping of radical intermediates (*19*). We therefore considered a design that, if applied to an internal, reductively sensitive tag (such as Dha), could use diboron(4) compounds to act, in principle, as activatable reagents. In this way, it might allow enhancement of these previously low-yielding reductive protein pathways (Fig. 1C), giving access to unprecedented synthetic protein chemistries.

**Fig. 2.**
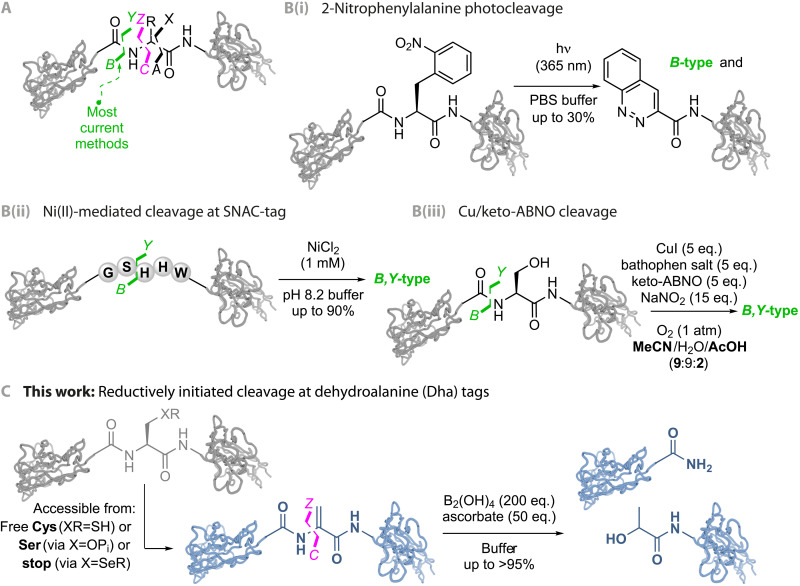
Selective chemical protein cleavage. (**A**) Modes of cleavage. Denoted here using the nomenclature of Roepstorff and Fohlman (*27*) as *A*,*X*-type, *B*,*Y*-type, and *C*,*Z*-type. (**B**) Known modes: (i) Noncanonical amino acids such as 2-nitrophenylalanine allow photolytic backbone cleavage under ultraviolet irradiation but proceed with lower efficiency (<30%) (*36*). (ii) Sequence-specific nickel-assisted cleavage (SNAC)-tag enables efficient Ni(II)-mediated cleavage (up to 90% after 18 hours) N-terminal of serine within a –GSHHW– sequence motif under ambient conditions and requires the introduction of a five amino acid cleavage tag (*34*). (iii) Cu/keto-ABNO (9-azabicyclo[3.3.1]nonan-3-one *N*-oxyl) oxidative cleavage facilitates serine-specific cleavage but requires relatively high concentrations of organic cosolvent, and so is not fully protein compatible/biocompatible (*35*). (**C**) This work: B_2_(OH)_4_-promoted Dha-selective protein cleavage following a direct-to-tag design described in Fig. 1 allows a rare *C*,*Z*-type cleavage.

Proteolytic cleavage (Fig. 2) is one of the most common posttranslational modifications and can be crucial for protein structure and function. It drives important biological processes such as zymogenesis, prohormone processing, and protein degradation, regulating a plethora of physiological and pathological pathways (*20*–*22*). In recent years, the exploitation of targeted protein degradation via direct or indirect control of proteolysis has seen a translational resurgence (*23*–*25*). A wide range of different proteases catalyze protein cleavage. These can display high sequence specificity (necessitating specific sequences for use), while others may be quite promiscuous (leading to nonselective product formation/degradation) (*22*, *26*). All operate via peptidic bond breaking [*B*,*Y*-type or C1-N2 cleavage using the nomenclatures of Roepstorff and Fohlman (*27*) and the International Union of Pure and Applied Chemistry (IUPAC) and the International Union of Biochemistry (IUB)joint commission (*28*), respectively] (Fig. 2A), exploiting nucleophilic addition-elimination.

In principle, more diverse proteolytic reactions can be considered. However, nonenzymatic chemical protocols for site-selective protein cleavage remain scarce; many classical chemical protocols lack selectivity and lead to protein denaturation and decomposition (see table S1) (*29*–*31*). More selective contemporary approaches exist. Some leading examples [see also (*32*, *33*)] include Ni-promoted (*34*) or Cu-promoted (*35*) cleavage and photochemical cleavage (*36*) but may require a specific, genetically introduced noncanonical amino acid (Fig. 2Bi) (*36*), an engineered sequence tag (Fig. 2Bii) (*34*), or do not proceed under purely aqueous (and so fully biocompatible) conditions (Fig. 2Biii) (*35*). Again, all result in C1-N2 or *B*,*Y*-type bond breaking. A controlled, reductively driven chemical protein cleavage with a new bond-breaking mode would therefore be of potential utility (Fig. 2C). Notably, oxidative cleavage mediated by the trapping of protein backbone radicals (e.g., by peptidylglycine α-hydroxylating monooxygenase) can drive peptide cleavage (*37*). Dha is readily accessible from low natural abundance residues—such as free cysteine (*38*), selenocysteine derivatives (*39*, *40*), or phosphoserine residues (*41*)—and, if addressed with new reactivity (*42*), could prove a useful tag for directed site-selective protein cleavage. Here, we explore proof-of-principle application using Dha to control reduction-initiated chemistries that allow an alternative mode of cleavage.

## RESULTS

As an initial model system to test possible inherent reductive reactivity, we first incubated diboron(4) with small-molecule dipeptide model substrate Ac-Gly-Dha-NHBn **1**. Encouragingly, despite the lack of an extensive peptidic environment (minimal Lewis basic motifs) for recruitment, treatment with tetrahydroxydiboron [B_2_(OH)_4_] displayed initial, albeit low, reactivity under simple aqueous conditions. Formation of acetyl glycinamide (as well as acetyl glycine) could be observed as N-terminal fragments arising from a putative reductive cleavage (fig. S4, A and B; see also fig. S14). We considered that the formation of acetyl glycine might proceed under these conditions from acetyl glycinamide via boric acid–catalyzed hydrolysis akin to previously observed transamidation (*43*). While therefore this model system did not allow a definitive conclusion on the mode (*B*,*Y*-type/C1-N2 versus *C*,*Z*-type*/*N2-C2) or mechanism of cleavage, these first results prompted further investigation in more complex protein systems.

**Fig. 3.**
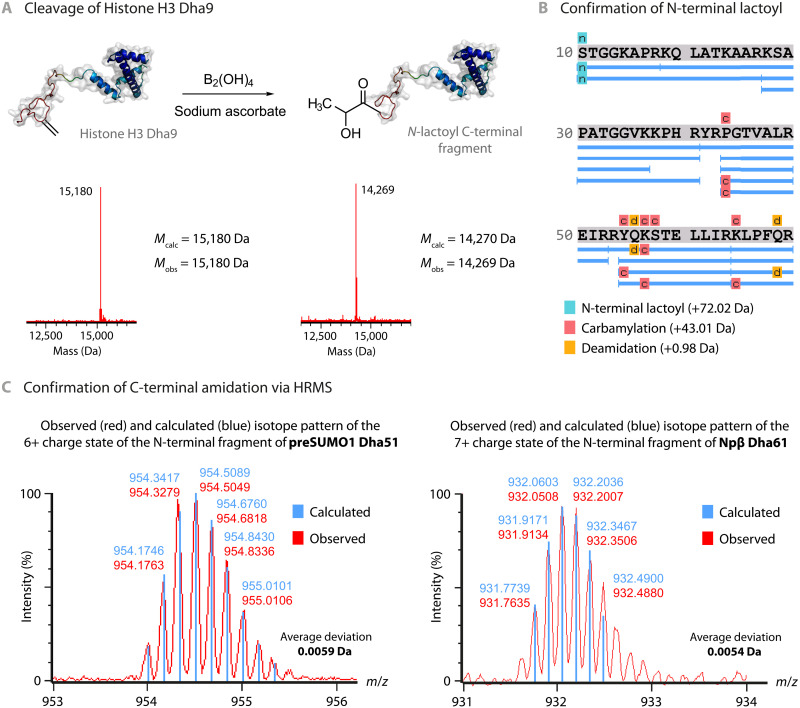
Analysis of the termini of the observed protein fragments reveals selective terminus editing. (**A**) N-terminal cleavage of histone H3 Dha9 leads to a C-terminal fragment carrying a *N*-lactoyl modification. (**B**) Confirmation of the lactoylated N-terminus by LC-MS/MS analysis (see also fig. S4). Note that carbamylation arises through the use of urea (*64*) during digestion (see also Materials and Methods). (**C**) Confirmation of the amidated C-terminus by high-resolution MS (HRMS) and analysis of the isotope patterns of the N-terminal fragments obtained after cleavage of preSUMO1-Dha51 (6+ charge state depicted here) and Npβ-Dha61 (7+ charge state depicted here). See figs. S5 and S6 for full details.

Next, diboron(4) compounds were tested with Dha-containing proteins [histone proteins eH3.1-Dha4, H3-Dha9, and H3-Dha10, prepared from Cys-containing precursors (*38*); see Materials and Methods] (fig. S4, C and D). Notably, after incubation with tetrahydroxydiboron [B_2_(OH)_4_] under ambient atmosphere, protein cleavage at a seemingly higher rate than in model system **1** was observed [after 30 min: histone H3-Dha9, 17.7 ± 0.7%; histone eH3.1-Dha4, 14.6 ± 0.2%; pentapeptide, 7.9 ± 0.1%; and dipeptide **1**, 6.3 ± 0.1% values are consumed Dha starting material (means ± SD, *n* = 3) as determined by liquid chromatography–mass spectrometry (LC-MS) and accompanied also by apparent nonspecific oxidation products (fig. S1A)]. Notably, the observed products suggested that cleavage occurred selectively and only N-terminal of Dha implying a unique mode of cleavage (Figs. 2C and 3A).

Reaction optimization and screening for suitable additives (fig. S1B) on model protein histone H3-Dha10 revealed that a combination of tetrahydroxydiboron and sodium ascorbate led to high cleavage efficiency (up to 88% after 3 hours and >95% after 24 hours at room temperature) while proceeding satisfactorily in a wide range of relevant buffers (sodium phosphate, Tris, HEPES, sodium acetate, and sodium borate) and at different pH values (pH 6 to pH 9; fig. S2). The additive sodium ascorbate allowed suppression of undesired protein oxidation while promoting cleavage. ^11^B nuclear magnetic resonance (NMR) experiments monitoring the effect of added ascorbate, in the presence of representative reactive oxygen species (ROS) hydrogen peroxide, revealed a clear protective effect upon diboron reagent; degradative decomposition to B(OH)_3_ was accelerated by ROS and mitigated by ascorbate (fig. S15). We investigated also whether the addition of mild transition metal reductants such as iron(II) or manganese(II) salts could have a similar effect. Addition of iron(II) sulfate led to increased oxidation without promoting cleavage, while manganese(II) sulfate promoted cleavage but was not able to inhibit oxidation.

We next investigated temperature dependence. The reaction was found to proceed best at elevated temperature (37°C), where full conversion was observed after 6 hours on model protein histone H3-Dha10. At room temperature, 86% conversion was observed after 6 hours and >95% after a day. Cleavage was found to even proceed at 4°C, albeit at a much slower rate. After 1 day, 73% conversion was observed and the reaction was found to proceed to completion (>95% conversion) after 4 days (fig. S3).

We next tested different diboron(4) [B_2_(OR′)_4_] compounds. To probe relative ligand ability, we varied substituents (R′ = H, catechol, pinacol; Table 1, entries 1 to 3). The reaction was found to proceed well with both tetrahydroxydiboron [B_2_(OH)_4_] and *bis*(catecholato)-diboron (B_2_cat_2_), while *bis*(pinacolato)diboron (B_2_pin_2_) gave reduced conversions. These results, coupled with lowered reactivity under acidic conditions, suggested modulation of ligand binding ability that correlated with reactivity (consistent with the design). They also reflect stability of B─B bond toward homolytic cleavage as judged by respective bond dissociation energies [B─B bond (kJ/mol): B_2_pin_2_: 460; B_2_(OH)_4_: 434] (*44*) (see also the mechanism discussion below).

**Table 1.** Site-selective cleavage efficiencies using a selection of boron-based reagents.

**Entry**	**Reagent**	Conversion*
1	B_2_(OH)_4_	79
2	B_2_cat_2_	80
3	B_2_pin_2_^†^	31
4	NaBH_4_	0
5	NaCNBH_3_	0
6	HBpin^‡^	0
7	HBcat^‡^	0

*Conditions: Histone H3-Dha10 in water (1 mg/ml, 66 μM), sodium ascorbate (50 equivalents), and reagent (200 equivalents) from freshly prepared aqueous stock solution. Incubation at room temperature for 3 hours. Analysis was performed by LC-MS and conversion is given in %..

†DMF [10% (v/v)] was used as organic cosolvent.

‡Stock solution in dry MeCN.

We also tested the possible role of potential decomposition products that might be formed under reaction conditions (Table 1, entries 4 to 7). Representative borohydrides, such as sodium borohydride (NaBH_4_) or sodium cyanoborohydride (NaCNBH_3_), did not afford any site-selective cleavage product and led to either protein degradation or no conversion at all. Similarly, no conversion was observed for either catecholborane or pinacolborane. This is notable as NaBH_4_ can slowly reduce Dha at much higher equivalents (*19*), and yet no reaction was seen; this again highlighted the postulated role of reagent association in enhancing reactivity.

The atypical mode of *C*,*Z*-type/N2-C2 cleavage indicated by intact protein MS (Fig. 3A) was unambiguously confirmed by proteolytic peptide mapping: The C-terminal fragment of cleavage bears an N-terminal lactamide (Fig. 3B). Furthermore, the exclusive presence of an α-amidated C-terminus [─CONH_2_ as a “*c*-type fragment” (*45*) rather than ─COOH as a “*b*-type fragment”) on the N-terminal fragment was confirmed by high-resolution MS of reaction mixtures obtained upon protein cleavage (preSUMO1-Dha51 and Npβ-Dha61; Fig. 3C).

**Fig. 4.**
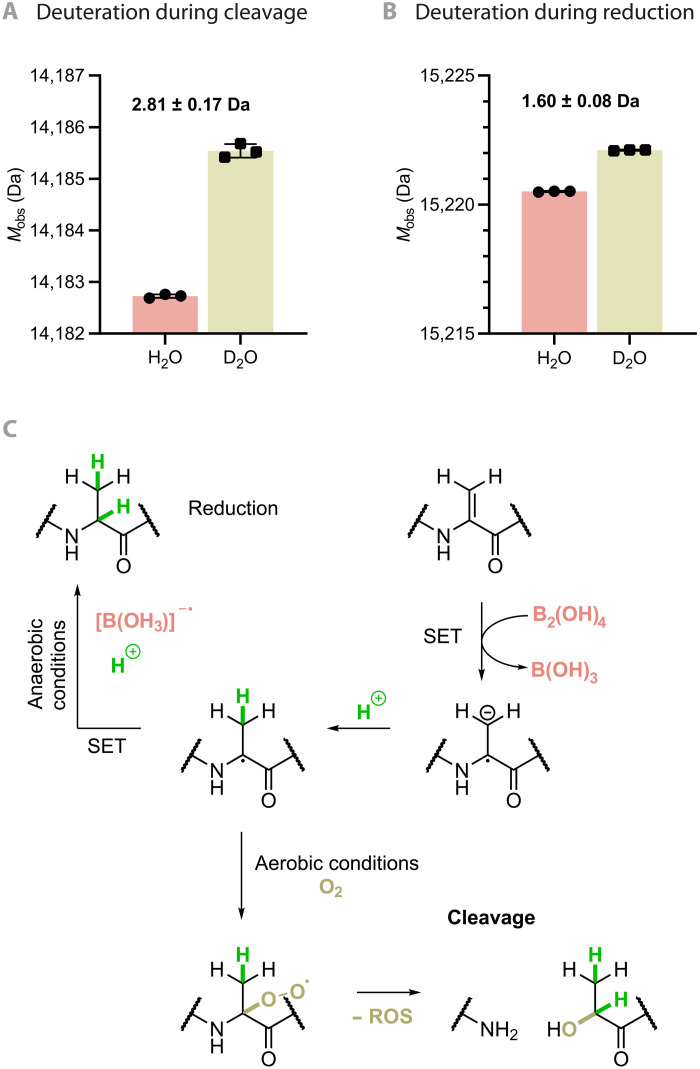
Proposed mechanism for the diboron-mediated protein cleavage at Dha residues. Isotopic labeling (**A** and **B**) suggests reductive single-electron transfer (SET) (**C**) consistent with different paths of reduction (B) and cleavage (A). The mechanism shown here is that in the absence of deuteration (see also fig. S8 for a deuteration mechanism, including additional possible contribution via keto-enol tautomerism in the pyruvamide prior to reduction to lactamide). Error bars depict means ± SD (*n* = 3).

Next, we probed the mechanism of this atypical reactivity. First, on-protein deuterium-labeling experiments gave further insight (Fig. 4); when reaction was conducted in D_2_O instead of H_2_O, the incorporation of ~3 deuterium atoms into the C-terminal cleavage fragment was observed (Fig. 4A). Second, notably, in the absence of atmospheric oxygen, no cleavage could be observed. Instead, reduction of Dha to Ala was observed under anaerobic conditions; in D_2_O, this was accompanied by incorporation of ~2 deuterium atoms. This highlighted a strict necessity for molecular (triplet) oxygen for cleavage and switchable product outcome dependent on conditions (anaerobic versus aerobic) (Fig. 4B). Last, in the presence of radical inhibitor TEMPOL (4-hydroxy-2,2,6,6-tetramethylpiperidin-1-oxyl), inhibition of both cleavage and oxidation was observed (figs. S1 and S8).

Together, these data suggested the involvement of a radical mechanism (Fig. 4C). While full mechanisms of diboron-initiated radical transformations have not been fully elucidated, B_2_(OH)_4_ has been applied as an apparently reductive radical initiator (*17*, *46*). Single-electron reduction of Dha would lead to the formation of a radical anion intermediate, leading to incorporation of one H/D from solvent. Under anaerobic conditions, a second single-electron transfer and subsequent protonation/deuteration would lead to the observed reduced Ala product, featuring the incorporation of two H/D from solvent (Fig. 4B). However, under aerobic conditions, the C_α_• radical would readily react with triplet oxygen; subsequent cleavage would result in formation of primary amide and ketoamide (*47*). These ketoamides are also reductively sensitive; small-molecule experiments (fig. S7) confirmed that reduction of ketoamide to corresponding lactamide by B_2_(OH)_4_ is feasible under such conditions. In model system **1**, when predicted C-terminal fragment *N*-benzyl pyruvamide **2** was treated with B_2_(OH)_4_, only reduction to corresponding lactamide **3** could be observed. Formation of the pyruvamide and enolization before reduction to lactamide would also explain the higher degree of deuterium incorporation observed on protein when the cleavage reaction was conducted in D_2_O (fig. S8). To further probe this mechanism, we also monitored and compared the products of cleavage using ^1^H NMR in a model Dha-containing pentapeptide system in both water and D_2_O (fig. S14). Consistent with the suggested mechanism, we saw site-selective incorporation of deuterium at the α-carbon and methyl group of the lactamide moiety.

**Fig. 5.**
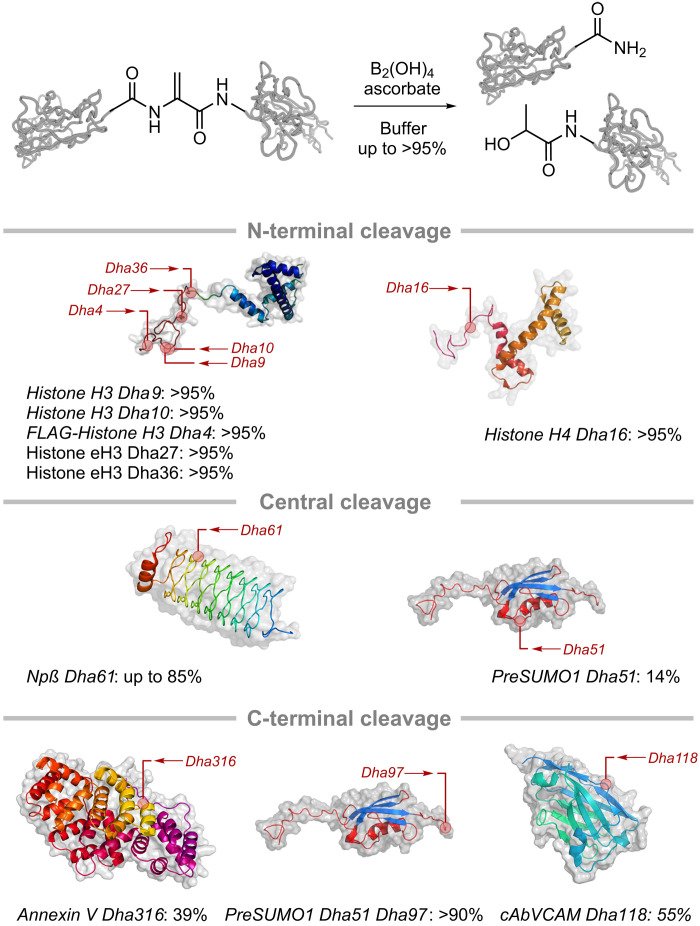
Scope of diboron-promoted radical protein cleavage at Dha residues. Different regions may be cleaved by inserting Dha at appropriate sites (N-terminal, central, and C-terminal). This allowed different access to short or long fragments with N- and C-terminal editing. For example, C-terminal–amidated proteins could be generated by placing Dha toward the C-terminus (see Fig. 6). Selective cleavage was observed in proteins with Dha at multiple sites (e.g., preSUMO1-Dha51-Dha97) or in the presence of disulfides (e.g., cAbVAM-Dha118). Indicative locations of cleavage sites are shown. Conditions for Dha formation: Histones: (i) 250 equivalents of dithiothreitol (DTT) [30 min at room temperature (RT)] and NaP_i_ [100 mM and 3 M Gdn·HCl (pH 8.0)]. (ii) 80 equivalents of 2,5-dibromohexanediamide (DBHDA), *N*,*N*′-dimethylformamide (DMF) (2 hours at RT and then 2.5 hours at 37°C), and NaP_i_ [100 mM and 3 M Gdn·HCl (pH 8.0)]. Npβ: (i) 50 equivalents of DTT (30 min at RT) and NaP_i_ [50 mM (pH 7.8)]. (ii) 500 equivalents of DBHDA, DMF (2 hours at 37°C), and NaPi [100 mM (pH 8.0)]. PreSUMO1-Dha51: (i) 50 equivalents of DTT (30 min at RT) and NaPi [50 mM (pH 8.0)]. (ii) 500 equivalents of methyl 2,5-dibromopentanoate (MDPBA), dimethyl sulfoxide (DMSO) (14 hours at 37°C) and NaPi [100 mM (pH 8.0)]. Annexin V: (i) 50 equivalents of DTT (20 min at RT) and NaP_i_ [50 mM (pH 7.8)]. (ii) 100 equivalents of MDBPA in DMSO (45°C at 3.5 hours), 50 equivalents of MDBPA in DMSO (25 min at 45°C), and NaP_i_ [100 mM (pH 8.0)]. PreSUMO1-Dha51-Dha97: (i) 12.5 equivalents of *N*-methyl-2-chloropyridinium iodide (10 min at 4°C) and NaP_i_ [50 mM (pH 8.0)]. (ii) NaOH (60 min at 4°C), NaP_i_ [50 mM and (pH 8.0)], and NaOAc [200 mM (pH 4.0)]. cAbVCAM: (i) 10 equivalents of DTT (30 min at 25°C). (ii) 250 equivalents of DBHDA and DMSO (2 hours at 37°C). Conditions for cleavage: Histones: 50 equivalents of sodium ascorbate, 200 equivalents of B_2_(OH)_4_ (17 to 24 hours at RT), and Milli-Q H_2_O. Npβ, annexin V: 5 mM sodium ascorbate, 20 mM B_2_(OH)_4_ (24 hours at 45°C), and sodium borate [100 mM (pH 8.0)]. *PreSUMO1-Dha51*: 2.5 mM sodium ascorbate, 10 mM B_2_(OH)_4_ (48 hours at RT), and Tris [100 mM (pH 8.0)]. PreSUMO1-Dha51-Dha97: 100 equivalents of sodium ascorbate, 400 equivalents of B_2_(OH)_4_ (18 hours at RT), and Tris [100 mM (pH 8.0)]. cAbVCAM: 125 equivalents of sodium ascorbate, 500 equivalents of B_2_(OH)_4_ (18 hours at RT,), and NaP_i_ [500 mM (pH 8.0)].

Next, the breadth of protein substrate was explored. A range of proteins differing in size, fold, and Dha-tag cleavage site was subjected to the optimized cleavage conditions (Fig. 5). Thus, histone-H3 and histone-H4 (small α-helical proteins with accessible tails), Npβ (pentapeptide repeat, β-helical), annexin V (globular α-helical protein), and preSUMO1 (SUMO1 precursor protein consisting of α-helices and β-sheets) were used as substrates (Fig. 5). Consistent with a postulated need for dynamic accessibility in the direct-to-tag manifold (Fig. 1Aiii), reactivity correlated strongly with site accessibility (as determined by the solvent accessible surface area; table S2). The method also proved compatible with disulfide-containing proteins; application of the method to a single-domain, anti-VCAM antibody that has been shown to be suitable for targeting to atherosclerotic lesions (*48*) also proved successful with retention of disulfide integrity (Fig. 5 and fig. S13).

Essentially, quantitative conversions (>95%) were observed for highly accessible terminal sites in several proteins: histone H3-Dha9, histone H3-Dha10, FLAG-histone H3-Dha4, histone eH3-Dha27, histone eH3-Dha36, and histone H4-Dha16. In contrast, only low conversion (~10%) was observed at a Dha residue in a central region (for preSUMO-Dha51) with limited accessibility. Moreover, while little to no conversion was observed initially for Npβ-Dha61 and annexin V–Dha316, greatly enhanced cleavage conversions could be achieved by increasing accessibility through partial melting. Thus, by heating reaction mixtures to 45°C just below their determined *T*_M_s (Npβ-Cys61 = 51°C; annexin V–Cys316 = 52°C), yields were greatly increased for annexin V (<5% → 39%) and Npβ (<5% → 85%).

Next, we explored one aspect of the potential functional utility of the products of this atypical *C*,*Z*-type cleavage reaction. The potential to now precisely generate C-terminal α-amidation allows access to a natural post-translational modification that is widely found on proteins and peptides and yet can be difficult to introduce synthetically, usually requiring additional enzymatic processing steps or coexpression of a suitable enzyme (*49*–*51*). While the biological function of this modification remains under debate in a protein context, C-terminal α-amidation is essential for the biological activity of certain therapeutic peptides (*52*) and has been proposed to modulate circulation half-life through generation of resistance to endopeptidases.

**Fig. 6.**
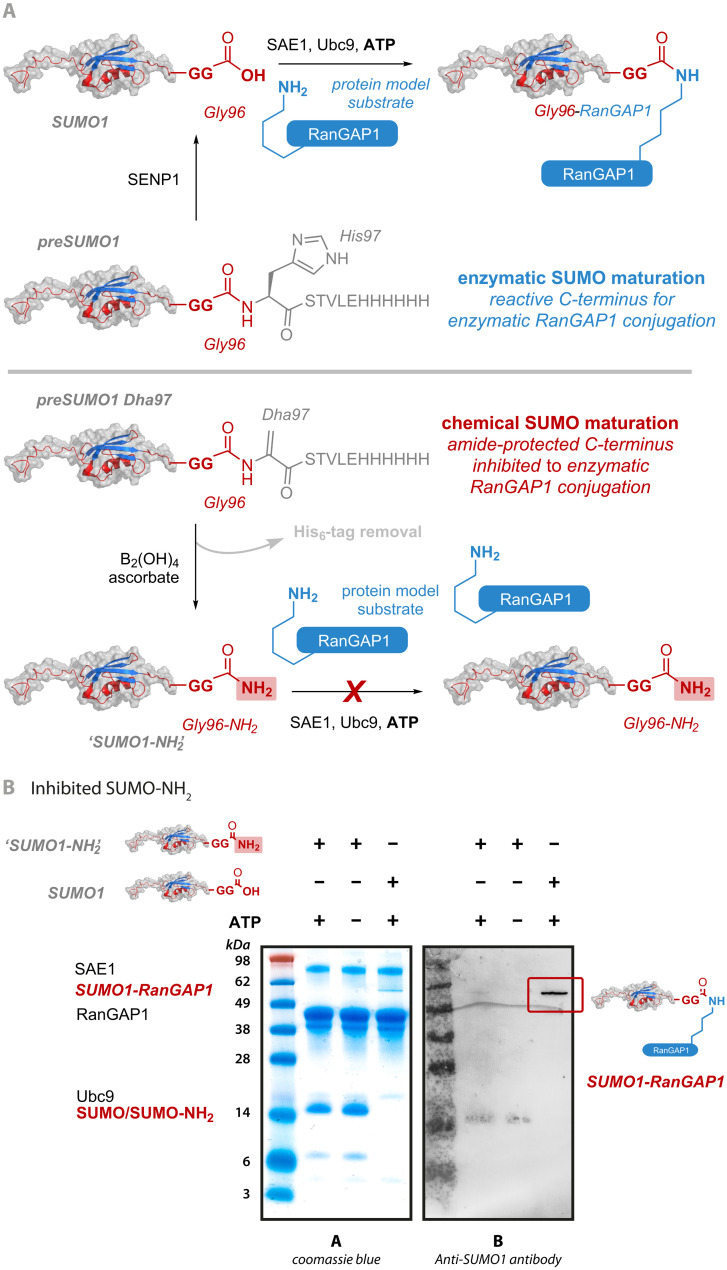
C-terminal editing allows creation of “SUMO-NH_2_.” (**A**) Comparison of enzymatic (top) and chemical maturation (bottom) in a reconstituted SUMOylation cascade. Diboron-promoted preSUMO1 maturation leads to a SUMO1-NH_2_ variant that instead bears a C-terminal CONH_2_ in contrast to the naturally matured C-terminal COOH SUMO1. (**B**) Introduction of an α-amidated C-terminus directly generated an inhibited SUMO1 variant that cannot participate in SUMOylation. The absence of a C-terminal carboxylate prevents SUMOylation of model protein fragment RanGAP1. While the SUMOylation cascade proceeded efficiently using native SUMO1, the cascade halted with the inhibited SUMO1-NH_2_ variant.

Our method gives potentially facile access to simultaneous C-terminal tag removal and α-amidation, a transformation that, to the best of our knowledge, has not been described previously. To test this, we expressed a C-terminally His_6_-tagged variant of preSUMO and installed an additional Dha cleavage site at its natural maturation site, which is normally recognized by its processing enzyme Sentrin-specific protease 1 (SENP1) (site 97; Fig. 6) (*53*). Subsequent SUMOylation typically proceeds via activation of the C-terminal carboxylate through adenosine 5′-triphosphate (ATP)–dependent thioester formation with SUMO-activating enzyme subunit 1 and subsequent transfer to Ubc9, catalyzing the formation of an isopeptide bond between the C-terminus and lysine residues (*54*).

Consistent with our prior observations on accessibility (see above), this accessible C-terminal site allowed ready *C*,*Z*-type cleavage diboron–promoted preSUMO1 “maturation” to a SUMO1-NH_2_ variant that instead bears a C-terminal CONH_2_ in contrast to the naturally matured C-terminal COOH SUMO1. C-terminal SUMO amides have been previously shown to inhibit SUMOylation cascades (*55*). Consistent with this, the introduction of an α-amidated C-terminus directly generated an “inhibited” SUMO1 variant that could not participate in SUMOylation (Fig. 6). The absence of a C-terminal carboxylate prevented SUMOylation of model protein fragment Ran GTPase-activating protein 1 (RanGAP1); while the SUMOylation cascade proceeded efficiently using native SUMO1, the cascade halted with the inhibited SUMO1-NH_2_ variant. Moreover, exploitation of the relative accessibility of the C-terminal region of preSUMO over its inaccessible central region (see above) allowed us to regioselectively cleave at accessible site Dha97 (>90% cleavage after 18 hours at room temperature) over that at Dha51 (~20%) (fig. S9, A and B), creating a SUMO-NH_2_ variant bearing a Dha51 site that in turn then allowed further reaction as a “tagged” and inhibited SUMO (fig. S9A). This dual-differentiated Dha reactivity (i.e., *C*,*Z*-type cleavage amidation at one Dha site and conjugate addition at another) raises exciting future potential to allow ready dual function (e.g., inhibition and pull down/retrieval) using such “edited” protein systems.

**Fig. 7.**
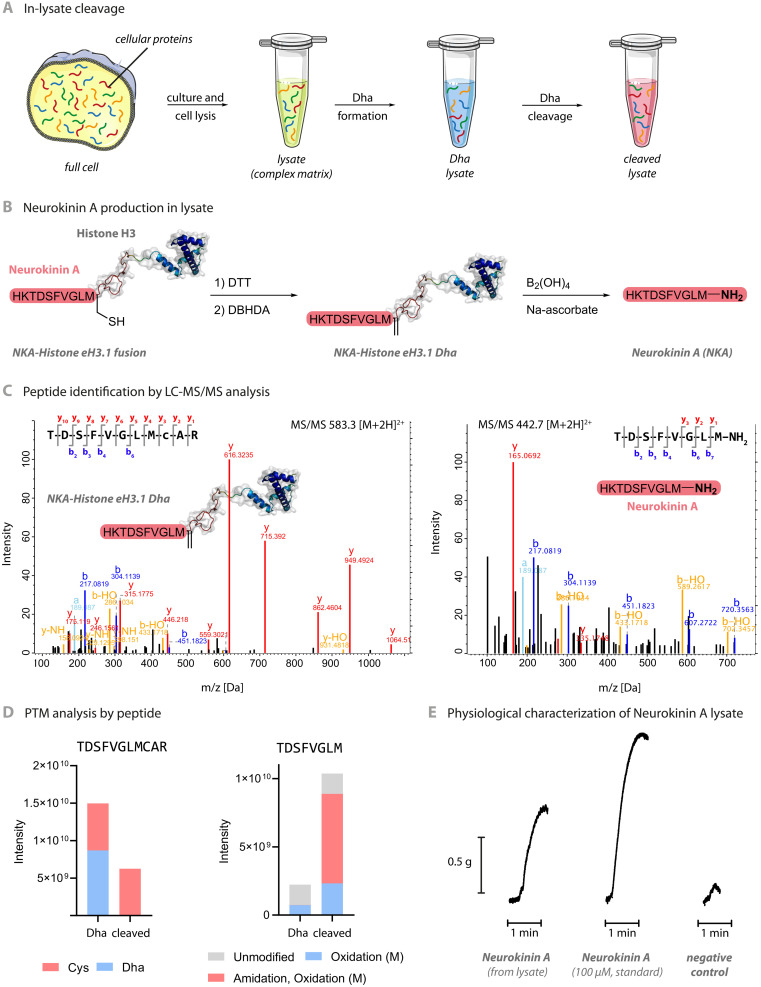
Diboron-promoted protein cleavage in a complex biological matrix. (**A**) In-lysate cleavage enables the direct production of cleaved proteins and peptides from a cellular proteome as a model complex biological matrix. (**B**) Construction of a genetic histone eH3.1 fusion construct for putative production of tachykinin neuropeptide NKA allowed testing of the in-lysate cleavage technology (see also fig. S10 for Western analyses in other representative proteomes). (**C**) MS/MS spectra for the peptides show successful detection of the Dha precursor protein (left) and product, cleaved NKA, (right) within reacted proteomes (see also fig. S12 for global analyses in other representative proteomes). *m*/*z*, mass/charge ratio. (**D**) LC-MS/MS total ion count (TIC) intensity analyses of precursor- (left) and product-associated (right) peptides also reveal consumption of Dha precursors (“Dha”) upon submission to cleavage conditions (“cleaved”). The intensity of modified peptides was normalized based on sum of intensity of unconverted TDSFVGLMCAR peptide. (**E**) Activity-based physiological characterization in a murine uterine contraction model reveals tachykinin activity from this treated proteome and confirms biological function only after cleavage. Uterine tissue in an organ bath was stimulated with treated lysate containing in-proteome–generated NKA, and the contractile force was recorded (*y* axis) over time (*x* axis) and compared with the response from positive control (commercial standard NKA). Both showed a clear contractile response compared to the negative control (untreated lysate). The experiment was repeated using five tissue samples from two different animals, and representative traces are shown.

Last, to test the applicability of these methods beyond in vitro conditions, we examined the application of these methods to more complex biological mixtures (Fig. 7). First, we tested the potential of the cleavage reaction through direct application to a representative wild-type proteome [derived from lysis of *Escherichia coli* BL21(DE3)]. Proteomic analysis of sequential global, in-lysate formation of Dha-containing proteins [using bis-alkylation–elimination reagent 2,5-dibromohexanediamide (DBHDA)] and then diboron-mediated cleavage revealed successful generation and then consumption of Dha-containing peptides (fig. S12). Second, we tested the applicability of this method to two different epitope-tagged proteomes containing an antigen marker (HA_98–106_ nonapeptide) in putative protein targets detectable by Western analysis; in both cases, these not only suggested minimal loss of the proteome upon such chemical transformation but also revealed direct in-proteome formation of cleaved epitope-tagged product (fig. S10). Last, we explored the potential of this in-proteome method to directly generate pharmacological activity via C-terminal amidation. The tachykinins represent an intriguing family of ancient neuropeptides found widely among the animal kingdom and have been implicated in diverse roles in both health and disease, including many homeostatic mechanisms such as pain, inflammation, immunity, and hormonal regulation (*56*). Of these tachykinin peptides, neurokinin A (NKA) is an archetypal peptide (*52*) with activity that typically depends critically on its formation as a C-terminal amide [amide: acid activity ratio of >10,000:1 (*57*)]. We therefore generated a proteome with the potential to contain tachykinin precursors that would directly drive pharmacological activity upon in-proteome cleavage (Fig. 7, B to E). Following application of the tested, in-proteome procedure to this putative “pretachykinin proteome,” tryptic-MS^n^ proteomic analysis revealed successful detection not only of required Dha-containing precursor protein (Fig. 7C, left) but also of subsequent product, cleaved NKA (Fig. 7C, right), within reacted proteomes. Intensity analyses of precursor- (Fig. 7D, left) and product-associated (Fig. 7D, right) peptides also revealed consumption of Dha precursors upon submission to cleavage conditions. Notably, activity-based physiological characterization in a murine uterine contraction model (rat uterus smooth muscle, 15-mm-long section; Fig. 7E) revealed clear tachykinin activity directly from treated proteome; untreated proteome as a negative control showed negligible activity. This physiologically relevant activity that is derived directly from a proof-of-principle “chemically altered” proteome raises exciting future potential to allow not only discovery of de novo neuropeptides (or other activities) but also possibly even activity-guided evolved function.

## DISCUSSION

In summary, we describe here an apparent free-radical procedure for the directed cleavage of protein backbones at readily introduced Dha tags. This method is enzyme and metal free, is operationally simple, and only requires inexpensive and commercially available reagents. Protein cleavage proceeds under biocompatible conditions in a range of relevant buffers and within a range of relevant temperatures (4° to 37°C). We have furthermore shown that this methodology is compatible with a range of different proteins and conditions.

Numerous recombinant therapeutic proteins are expressed either as their zymogen form requiring further proteolytic processing or carrying tags that need to be removed (*50*, *58*). We speculate that the methodology presented here could allow cost-efficient and protease-free zymogenesis. Furthermore, diboron-mediated radical cleavage could provide a general method for the removal of affinity tags while allowing for high specificity, omitting the need to introduce multiple residue recognition sites required for enzymatic cleavage and simplifying postcleavage purification as no protease has to be separated from the truncated protein.

Moreover, the selective editing of protein termini is now possible with this method. C-terminal amidation is known to modulate peptide function (*52*, *59*) and to modulate biomolecule half-life (*60*). The ability to readily create C-amidated proteins using the synthetic protein chemistry that we disclose here now allows this tactic to be applied to full-length biologics and even within complex proteomes.

We speculate based on design, observed enhanced reactivity (fig. S11) and modulation of ligand ability, that diboron compounds such as B_2_(OH)_4_ might interact with proteins through transient Lewis acid-base interactions (e.g., with backbone carbonyls or side chains. Activation of diboron reagents for homolytic B─B bond cleavage by Lewis bases is known (*46*, *61*–*63*). We should note that despite attempted observation of direct interaction (e.g., via protein NMR; fig. S16), we do not see evidence for the population of a clear pre-equilibrium akin to that we used for the basis of our design (Fig. 1Aiii), and therefore, the hypothesis of direct-to-tag is not proven. Nonetheless, any possible interaction regime has been limited to values that are reasonable (fig. S16), and we do observe rate enhancements for cleavage in protein scaffolds over not only dipeptide motifs but also pentapeptide motifs bearing the same primary sequence environment (fig. S11). We therefore cannot exclude other mechanisms (e.g., direct activation by other Lewis bases and even water) or even the operation of parallel combined mechanisms. Nonetheless, the enhancement in a protein environment suggests a key role for the protein as substrate; the mechanistic basis for this will be the subject of ongoing investigations. If operational, then such dynamic recruitment with activation, akin to protein cofactors, coupled with dynamic direction to functional groups, akin to ribozymes, that we propose here may suggest unprecedented modes of synthetic protein chemistry.

## MATERIALS AND METHODS

### General Dha formation protocol

Lyophilized Cys mutant protein and dithiothreitol (DTT) (250 equivalents) was dissolved in NaP_i_ buffer [100 mM (pH 8.0)] by repeated vortexing and sonication. The solution was incubated at room temperature for 1 hour before being desalted using GE MiniTrap G-25 desalting columns preconditioned with NaP_i_ buffer [100 mM (pH 8.0)] according to the manufacturer’s specifications. A solution of DBHDA (80 equivalents) in *N*,*N*′-dimethylformamide was added, and the mixture was briefly vortexed and shaken at room temperature for 2 hours. The temperature was raised to 37°C, and shaking was continued until completion of the reaction was determined by LC-MS. The solution was purified using GE MiniTrap G-25 columns preconditioned with NaP_i_ buffer [100 mM (pH 8.0)]. The sample was aliquoted, frozen in liquid nitrogen, and stored at −80°C. Addition of 3 M Gdn·HCl in all buffers can be used to accelerate reactions for less accessible sites.

### General protein cleavage protocol

Protein Dha mutant was dissolved in Milli-Q water or cleavage buffer [sodium borate, Tris, or NaP_i_ (pH 8.0)]. Stock solutions of tetrahydroxydiboron (5.6 mg in 1000 μl) and sodium ascorbate (14.0 mg in 500 μl) in Milli-Q water were prepared freshly directly before the reactions were conducted. Typically, 200 equivalents of tetrahydroxydiboron and 50 equivalents of sodium ascorbate were added from the previously prepared stock solutions, and the reaction mixtures were incubated at room temperature until no further progression of the reaction could be observed. Note that, for some protein substrates, higher reagent concentrations or reaction temperatures might improve cleavage conversion. The reaction can be conducted at lower temperatures (e.g., 4°C) if longer reaction times are accounted for.

### Proteolytic peptide mapping

#### ArgC digestion

Fifty micrograms of protein sample was dissolved in freshly prepared denaturing buffer (50 μl; 4 M urea and 100 mM ammonium bicarbonate) and incubated at room temperature for 10 min. The sample was diluted to a final concentration of 2 M urea using a 100 mM ammonium bicarbonate solution. CaCl_2_ was added to a final concentration of 2 mM. Activation solution (50 mM DTT and 5 mM EDTA in water) was added [1:10 (v/v)] before ArgC (1 μg; sequencing grade, Promega, catalog no. V1881) was added. The sample was shaken at 37°C (800 rpm) for 20 hours. The reaction was quenched by addition of formic acid [5% (v/v)] and centrifuged (30 min, 14,000 rpm, 4°C). The sample was desalted using a C_18_ resin containing tip and lyophilized in a protein LoBind microcentrifuge tube (Eppendorf, catalog no. 0030108116).

#### Data acquisition

Resulting peptides were separated using a nanoflow reversed-phase LC Ultimate 3000 UHPLC system (Thermo Fisher Scientific) and analyzed using a Q-Exactive Hybrid Quadrupole-Orbitrap Mass Spectrometer (Thermo Fisher Scientific).

#### Data analysis

Data analysis was performed with PEAKS Studio 8.5 (Bioinformatics Solutions Inc.). The data were searched against the given protein sequence. The precursor mass tolerance was set to 15 parts per million, and the fragment mass tolerance was set to 0.5 Da. A maximum number of three missed cleavages and nonspecific cleavage at one end of the peptide were specified. Methionine oxidation (+15.99 Da), deamidation (+0.98 Da; asparagine and glutamine), and carbamylation (+43.01 Da; lysine) were set as variable posttranslational modifications. In addition, N-terminal lactoylation (+72.02 Da) was included in the search. A maximum of four variable modifications was defined, and a false discovery rate of 1% on peptide level was applied.

### Determination of cleavage conversion via protein LC-MS

Protein samples were analyzed on Waters Xevo G2-S QTof or Waters Xevo G2-XS QTof mass spectrometers equipped with a Waters Acquity UPLC. Separation was achieved using a Thermo Scientific ProSwift RP-2H monolithic column (4.6 mm × 50 mm) using water + 0.1% formic acid (solvent A) and acetonitrile + 0.1% formic acid (solvent B) as mobile phase at a flow rate of 0.3 ml/min and running a 10-min linear gradient as follows: 5% solvent B for 1 min, 5 to 95% solvent B over 6 min, 95 to 5% solvent B over 1 min, and 5% solvent B for 2 min. Spectra were deconvoluted using MassLynx 4.1 (Waters) and the “MaxEnt1” deconvolution algorithm with the following settings: resolution: 1.0 Da per channel; damage model: uniform Gaussian; width at half height: 0.4 Da; minimum intensity ratios: 33% (left) and 33% (right); and iterate to convergence. Conversions were calculated from peak intensities.

### SUMOylation assays

SUMOylation was conducted using a commercially available SUMOylation assay kit (Abcam, catalog no. ab139470) following the manufacturer’s instructions. Five microliters (approximately 0.3 mg/ml) of a solution of chemically cleaved preSUMO1 in Tris buffer [100 mM (pH 8.0)] were treated with Milli-Q water (10 μl), 10× SUMOylation buffer (2 μl), 20× Mg-ATP (1 μl), 20× SUMO E1 (1 μl), and 20× RanGAP1 (1 μl). Control reactions containing no Mg-ATP or 20× SUMO1 (1 μl) were conducted. The amount of Milli-Q water used was adjusted to achieve a total reaction volume of 20 μl. The reaction mixtures were incubated at 37°C for 60 min before being quenched by addition of 5× Laemmli buffer (5 μl) and heating to 95°C (10 min).

The reactions were analyzed by SDS–polyacrylamide gel electrophoresis (2-(*N*-morpholino)ethanesulfonic acid (MES) running buffer, 10% bis-tris precast gel, 200 V, 40 min, room temperature) and Western blot. Western blot analysis was conducted using the primary rabbit anti-SUMO1 polyclonal antibody supplied with the SUMOylation Kit (1:1000 dilution) and a secondary goat anti-rabbit immunoglobulin G–alkaline phosphatase fusion (1:1000 dilution; Sigma-Aldrich, catalog no. A3687). Nitro blue tetrazolium/bromochloroindolyl phosphate substrate solution (Thermo Fisher Scientific, catalog no. 34042) was used to visualize proteins. Blocking was conducted using 5% (w/v) skimmed milk powder in Tris-buffered saline with Tween 20.

### In-proteome tachykinin generation

#### Plasmid preparation

The human histone *eH3.1 (C96A/C110A)* gene with a C-terminal FLAG and hemagglutinin tag was subcloned into a pET3d vector using Nco I and Bam HI sites (Thermo Fisher Scientific, GeneArt service). The eH3.1-K27C and eH3.1-K36C mutants were generated by site-directed mutagenesis (QuickChange II Site-Directed Mutagenesis Kit, Agilent). The gene *NKA-eH3.1* encoding the neuropeptide NKA was constructed by fusion with the N-terminus of histone eH3.1 using the In-Fusion cloning kit (Takara Bio). A cysteine residue was inserted between the *NKA* and *eH3.1* gene as a handle for Dha formation and cleavage.

#### Expression of NKA-eH3.1 and lysate preparation

The constructed NKA-eH3.1 plasmid was used to transform *E. coli* BL21(DE3) competent cells and expressed as histone H3 with the N-terminally fused neuropeptide NKA. Cells were grown in 300 ml of LB media [supplemented with ampicillin (100 μg/ml)] at 37°C and induced with 1 mM isopropyl-β-d-thiogalactopyranoside when an OD_600_ (optical density at 600 nm) of 0.6 was reached. The incubation was continued for another 3 hours at the same temperature. Cell pellets were resuspended in 20 ml of wash buffer [50 mM Tris and 100 mM NaCl (pH 8.0)] supplemented with cOmplete Mini protease inhibitor cocktail (Roche) and sonicated using a microtip. The supernatant was separated by centrifugation (20,000 rpm, 20 min at 4°C) and discarded. To dissolve histone proteins, the cell pellets were mixed with 0.5 ml of dimethyl sulfoxide (DMSO) at room temperature for 10 min before being shaken with 5 ml of unfolding buffer [7 M Gdn·HCl, 20 mM Tris, and 10 mM DTT (pH 7.5)] for 1 hour. The mixture was centrifuged at 20,000 rpm for 10 min at room temperature. The supernatant was desalted into DBHDA reaction buffer [3 M Gdn·HCl and 100 mM sodium phosphate (pH 8.0)] via PD-10 columns (Cytiva). After measuring the concentration of the protein mixtures in *E. coli* lysates by bicinchoninic acid (BCA) assay (Pierce BCA protein assay kit, Thermo Fisher Scientific), 250 molar equivalents of DBHDA were added. The mixture was incubated at 37°C for 3 hours to form NKA(Dha)-eH3.1 and desalted into cleavage buffer [10 mM Tris (pH 8.0)] to remove excess DBHDA using PD-10 columns (Cytiva). The diboron cleavage was proceeded in the presence of 100 mM tetrahydroxydiboron and 25 mM sodium ascorbate. A 100-μg aliquot from each reaction solution (Cys, Dha, and cleavage) was digested with trypsin for LC-MS/MS analysis. The remaining cleaved lysate was lyophilized on a freeze dryer (Christ Alpha 204 LSCbasic) for later assays.

### Ex vivo testing of in-proteome–generated tachykinin

The ability of the lysates to contract rat uterus smooth muscle was tested in an organ bath with a chamber volume of 10 ml. Briefly, individual sections of uterus (15 mm long) from three nonpregnant Wistar rats (250 g) were suspended in modified physiological Kreb’s solution containing low calcium (1.26 mM) and low glucose (2.8 mM) at 25°C to minimize the spontaneous activity. Each uterus was tensioned to 1g, and the viability of the muscles was tested with 0.3 nM 5-hydroxytryptamine. The contractile response of the tissue to the sample preparation or NKA standard (100 μM final concentration) was compared. In each case, the tissue was allowed to stabilize before adding the test compounds, and the effect of the sample preparation was tested in three independent uterus preparations. The response of the tissue was recorded using the PowerLab data acquisition system [ADInstruments with a bridge amplifier, a calibrated force (g) transducers] and LabChart software (v8.1.19). The response was monitored for 30 s after the addition of each challenge, and the tissue was allowed to recover for at least 2 min before the application of the next compound/sample.

## Supplementary Materials

### This PDF file includes:

Supplementary Methods, Experimental and Characterization Data Figs. S1 to S66 Tables S1 to S6 References

## Notes

View/request a protocol for this paper from *Bio-protocol*.
