# Supplementary material for: Reductive site-selective atypical C,Z-type/N2-C2 cleavage allows C-terminal protein amidation
Source: Sci Adv. 2022 Apr 8;8(14):eabl8675. doi: 10.1126/sciadv.abl8675 (PMC8993120; doi:10.1126/sciadv.abl8675)
Supplement: Supplementary file 1 — Supplementary Methods, Experimental and Characterization Data Figs. S1 to S66 Tables S1 to S6 References [file sciadv.abl8675_sm.pdf]

Science Advances
AAAS

Supplementary Materials for

Reductive site-selective atypical *C,Z*-type/N2-C2 cleavage allows C-terminal protein amidation

Tim A. Mollner, Andrew M. Giltrap, Yibo Zeng, Yana Demyanenko, Charles Buchanan, Daniel Oehlrich, Andrew J. Baldwin, Daniel C. Anthony, Shabaz Mohammed, Benjamin G. Davis*

*Corresponding author. Email: ben.davis@chem.ox.ac.uk

Published 8 April 2022, *Sci. Adv.* **8**, eabl8675 (2022)
DOI: 10.1126/sciadv.abl8675

The PDF file includes:

Supplementary Methods, Experimental and Characterization Data
Figs. S1 to S66
Tables S1 to S6
References

Other Supplementary Material for this manuscript includes the following:

Dataset S1 to S3

1 Supplementary Figures

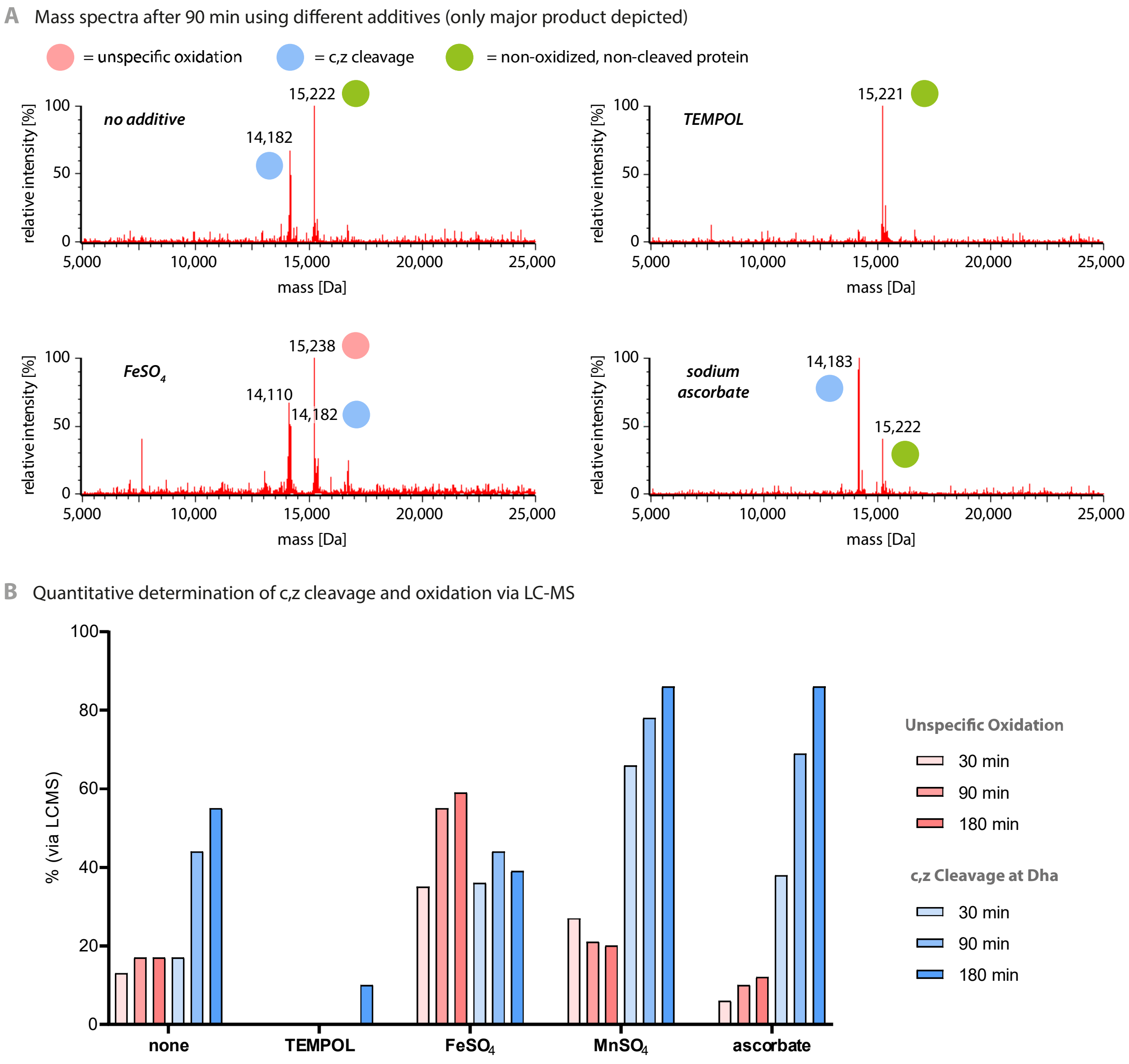

Supplementary Figure S1: **Ascorbate is an effective inhibitor of protein oxidation under tetrahydroxydiboron-promoted cleavage.** (**A**) Mass spectra for different additives after 90 min. (**B**) Quantification of time-dependent oxidation and c,z cleavage (n=1). Examples shown for Histone H3-Dha10. ‘Unspecific oxidation’ refers to an oxidized product (+16 Da from precursor) that was not identified, associated MS/MS analyses show heterogeneous peptides that suggest Met oxidation at various sites.

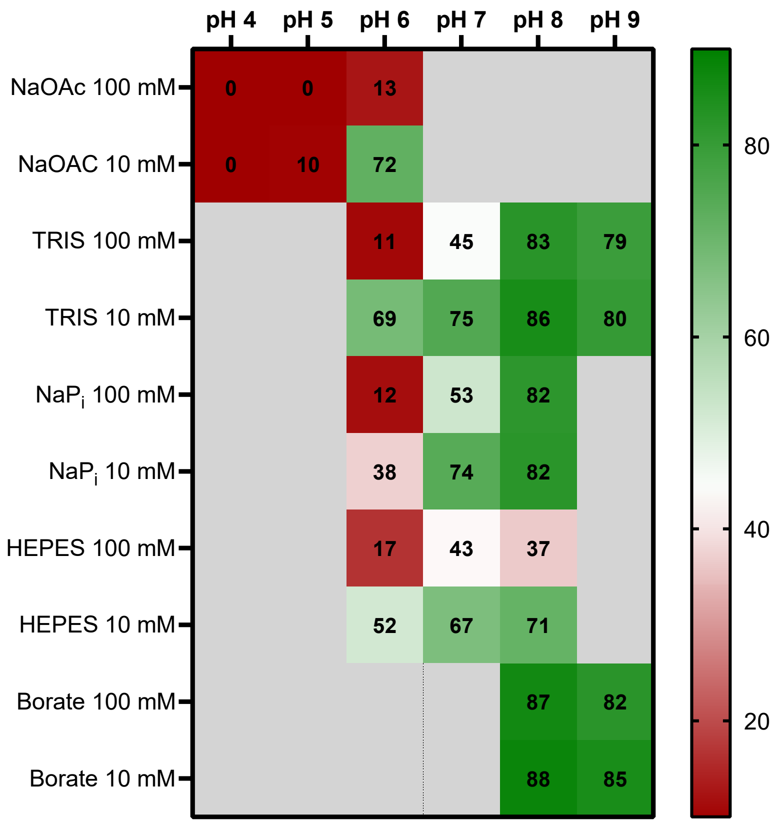

Supplementary Figure S2: Buffer screening reveals that tetrahydroxydiboron-promoted cleavage is compatible with a wide various common buffers. % Conversion to cleaved product observed during buffer screening for the tetrahydroxydiboron-promoted cleavage of Histone H3-Dha10 (1 mg/mL).

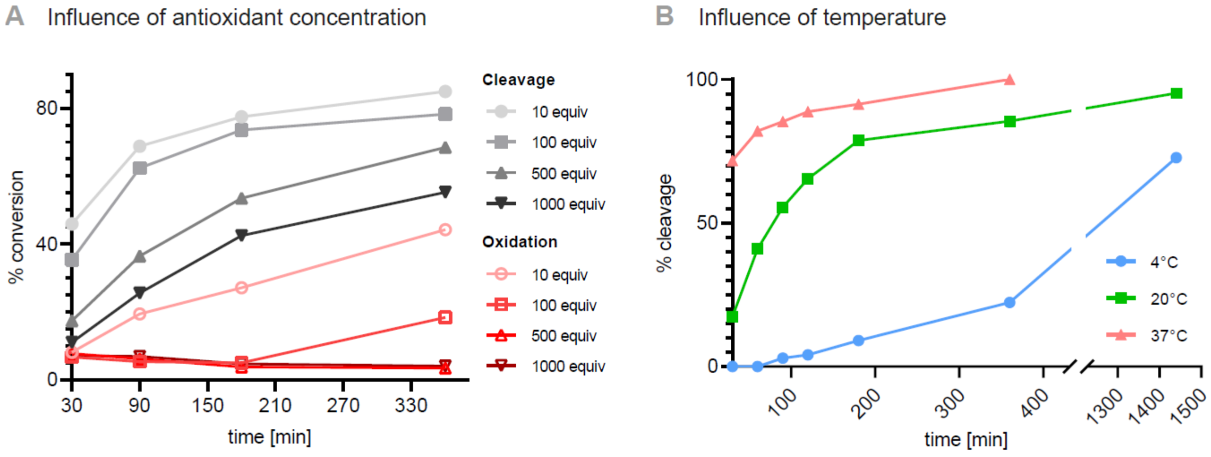

Supplementary Figure S3: The antioxidant concentration and reaction temperature have a large influence on reaction outcome. (**A**) Influence of the concentration of antioxidant sodium ascorbate on cleavage and oxidative damage as observed by LC-MS. (**B**) Temperature- and time-dependent progression of cleavage as observed by LC-MS. N.B. '% conversion' is used here to indicate a yield that is not isolated.

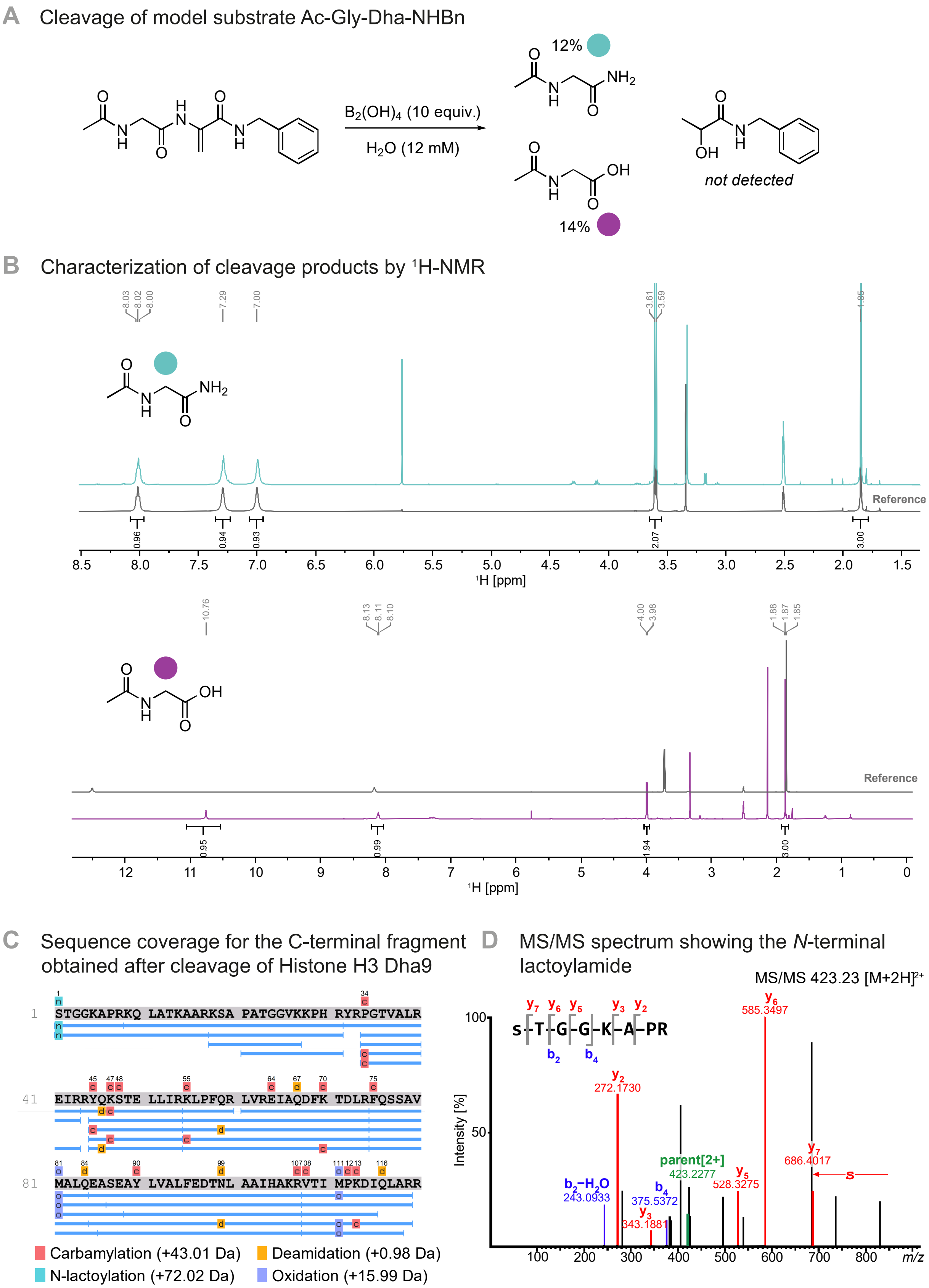

Supplementary Figure S4: Cleavage of model dipeptide 1 and MS/MS analysis of cleaved Histone H3 Dha9 confirm the identity of the proposed cleavage products. (A) Cleavage of model dipeptide **1**. The *N*-terminal fragments *N*-acetylglycine and *N*-acetylglycinamide were isolated and identified by

NMR via comparison to reference material. The *C*-terminal fragment could not be isolated. We speculate that under the conditions employed boric acid could promote amide hydrolysis akin to previously observed transamidation.*(41)* This would lead to not only the formation of *N*-acetylglycine from *N*-acetylglycinamide, but also to hydrolysis of *N*-benzyllactamide (C-terminal fragment). These hydrolysis products are volatile and were not observed. (**B**) Isolation, NMR analysis and comparison with a reference of two cleavage products: *N*-acetyl glycine (turquoise) and N-acetyl glycinamide (purple). (**C**) MS/MS analysis of cleaved Histone H3 Dha9. 95% sequence coverage were observed (1% FDR). Carbamylation is an artefact that stems from prolonged treatment with urea during digestion and is primarily found on primary amines (such as the *N*-terminus or Lys side chains).(*64*) (**D**) MS/MS spectrum of peptide fragment sTGGKAPR. s depicts the *N*-terminally lactoyl modified serine residue. −10lgP = 58.68.

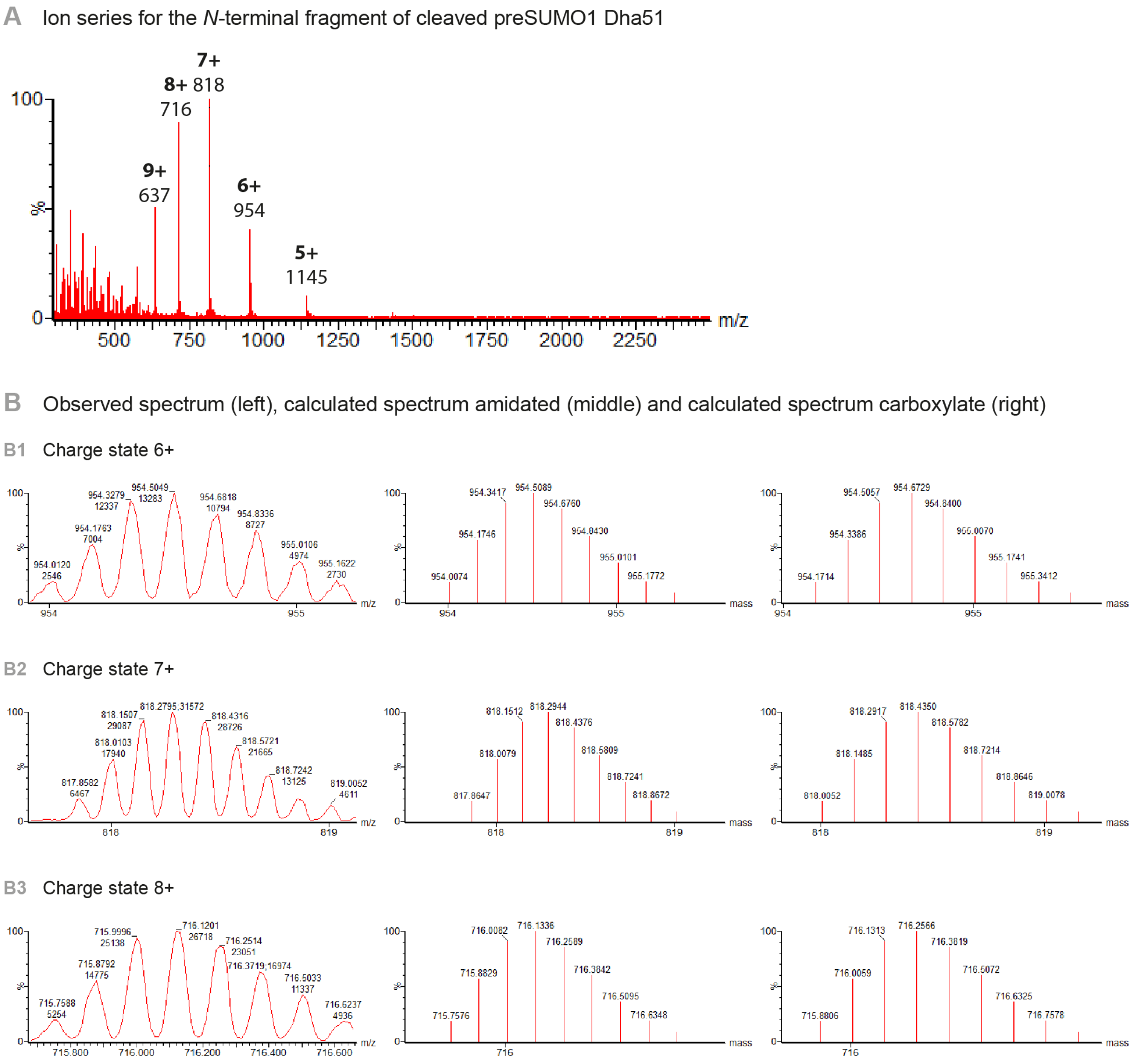

Supplementary Figure S5: Isotopic pattern analysis for the *N*-terminal fragment obtained after cleavage of preSUMO1 Dha51 confirms *C*-terminal amidation.

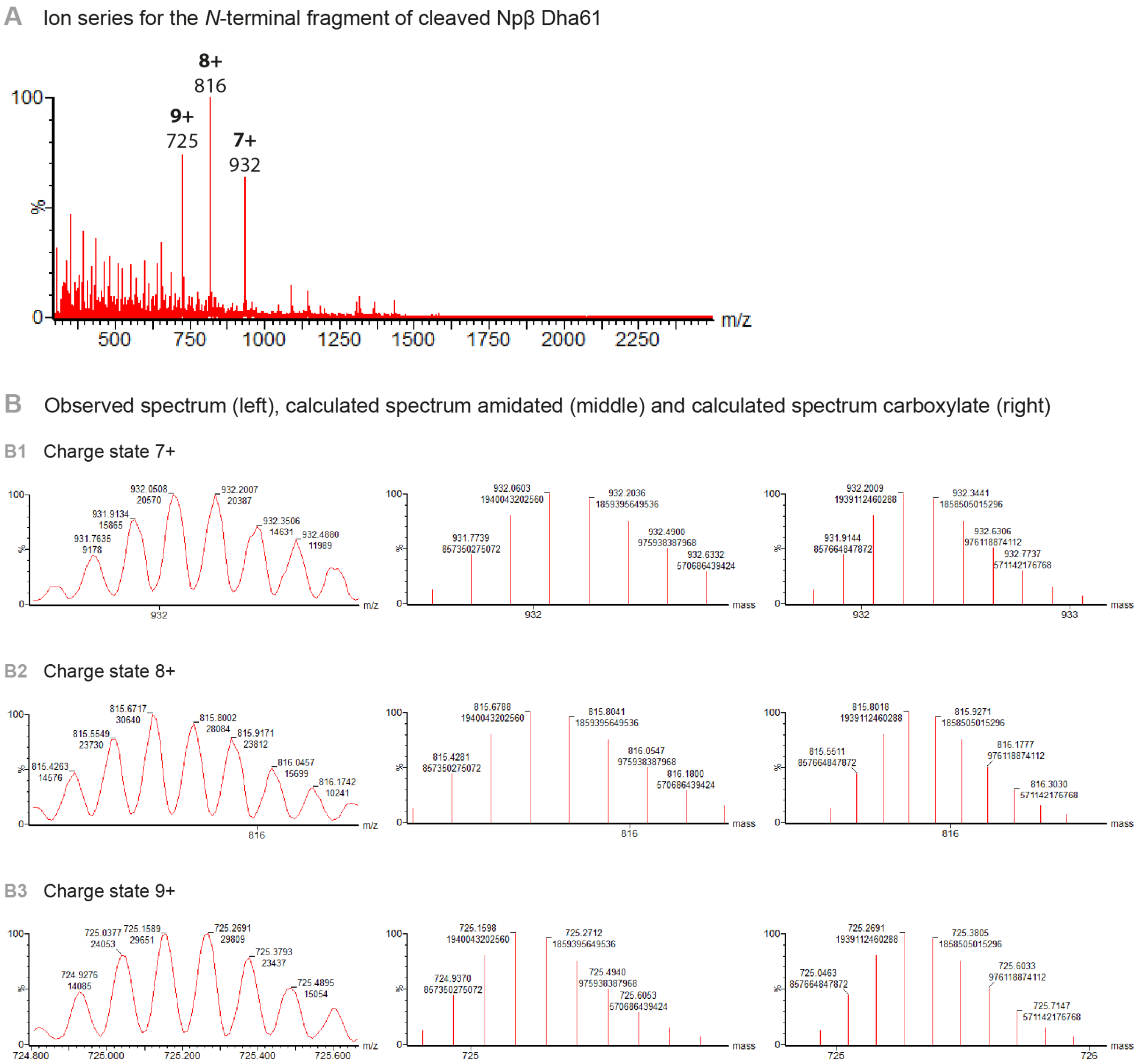

Supplementary Figure S6: Isotopic pattern analysis for the *N*-terminal fragment obtained after cleavage of Npβ Dha61 confirms *C*-terminal amidation.

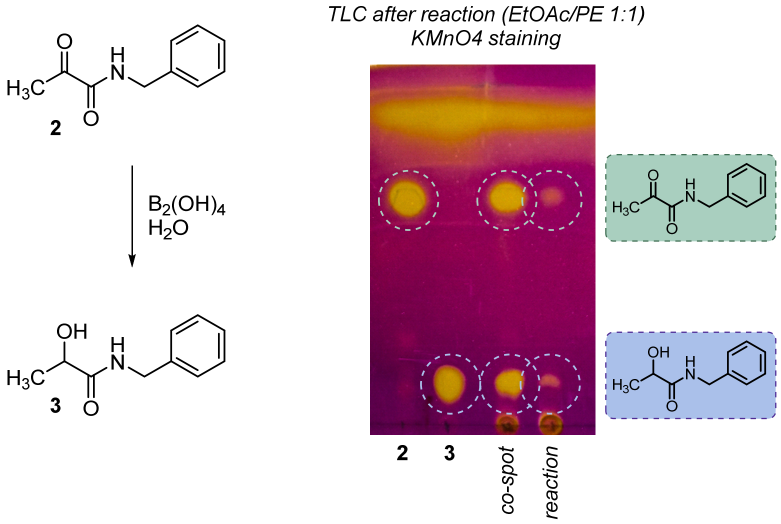

Supplementary Figure S7: Reduction of *N*-benzyl pyruvamide (2) to *N*-benzyl lactamide (3) is possible using tetrahydroxydiboron ($B_2(OH)_4$) in aqueous media.

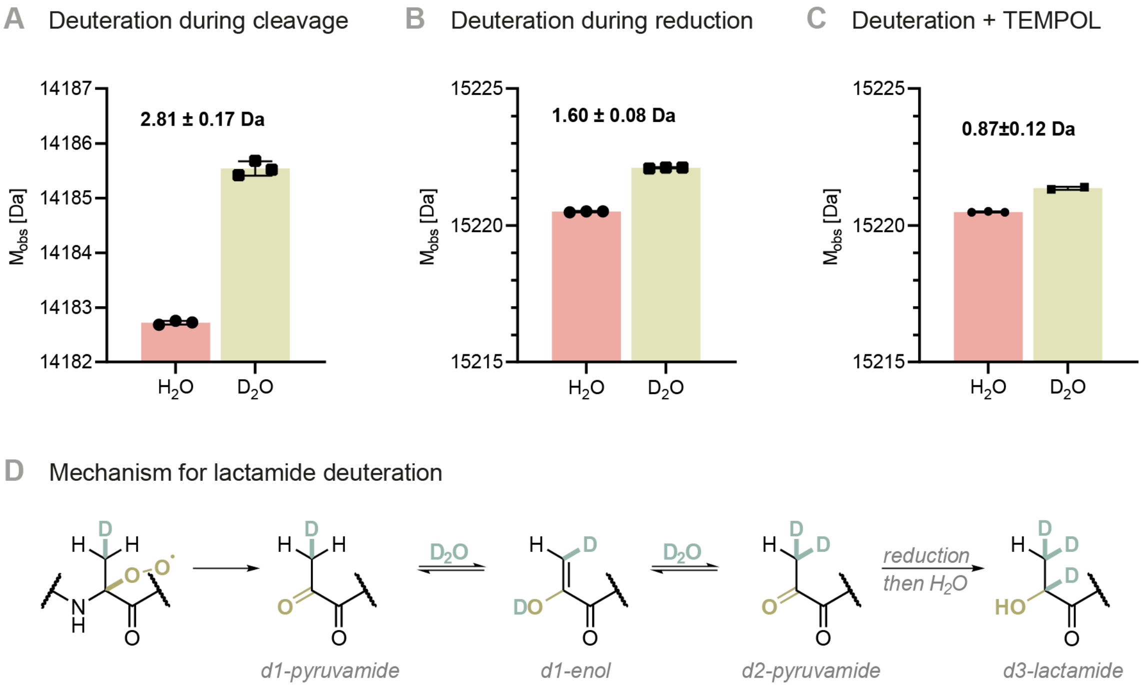

Supplementary Figure S8: Mechanistic investigations on protein using deuteration experiments reveal deuterium incorporation consistent with the proposed mechanism. Deuteration was observed by LC-MS and comparison of spectra obtained from reaction in H_2O and D_2O, respectively (n=3, mean±SD given). Spectra were deconvoluted using the MaxEnt1 algorithm as implemented in MassLynx 4.1 (Waters). The mass resolution was set to 0.04 Da/channel. (**A**) Deuteration under cleavage conditions (50 equiv. $B_2(OD)_4$, 200 equiv. sodium ascorbate, deuterium incorporation of the *C*-terminal, lactamide-containing fragment given) (**B**) Deuteration under reductive, anaerobic conditions. (**C**) Deuteration in presence of TEMPOL (50 equiv. $B_2(OD)_4$, 200 equiv. TEMPOL) (**D**) Proposed mechanism for lactamide deuterium incorporation.

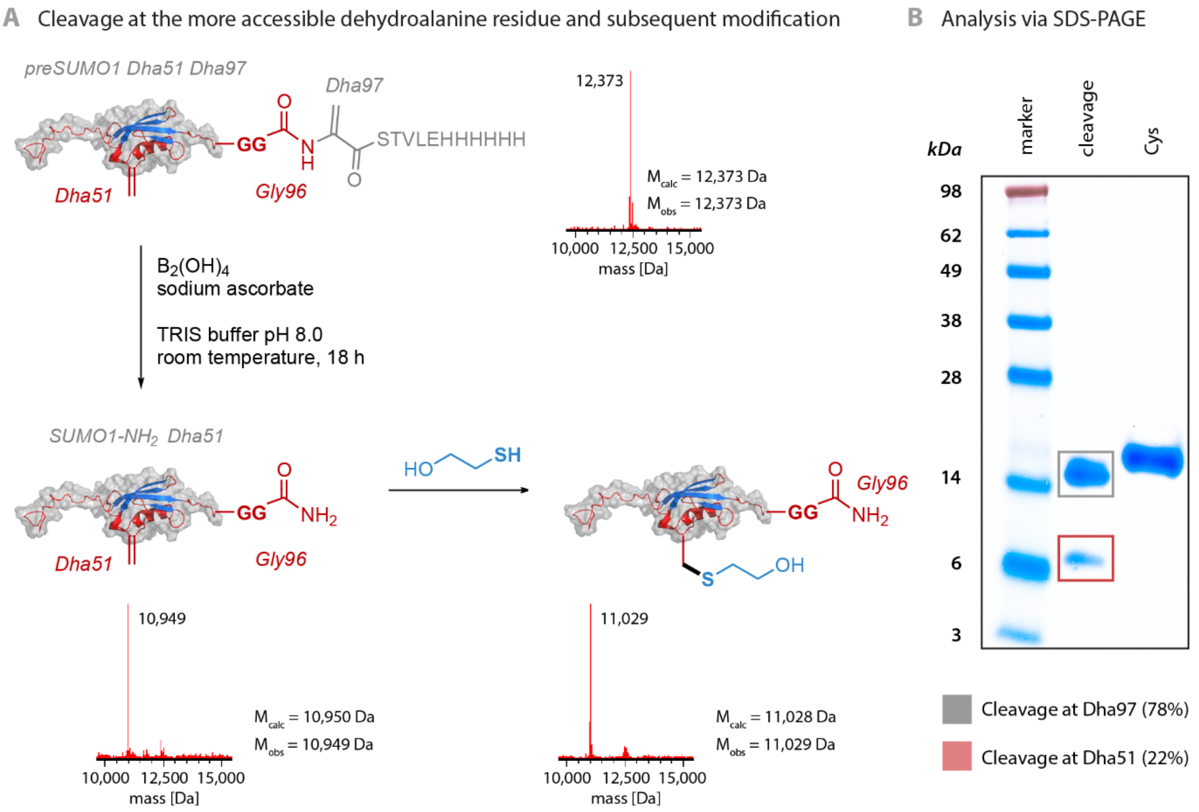

Supplementary Figure S9: Cleavage of preSUMO1 Dha51 Dha97 at the most accessible dehydroalanine residue proves selectivity based on accessibility. (**A**) Selective cleavage allows for subsequent modification via 'tag-and-modify' approach. (**B**) Cleavage at site 97 was determined to be 78% selective using SDS-PAGE and densitometry. Image analysis was conducted with ImageJ 1.53f.*(65)*

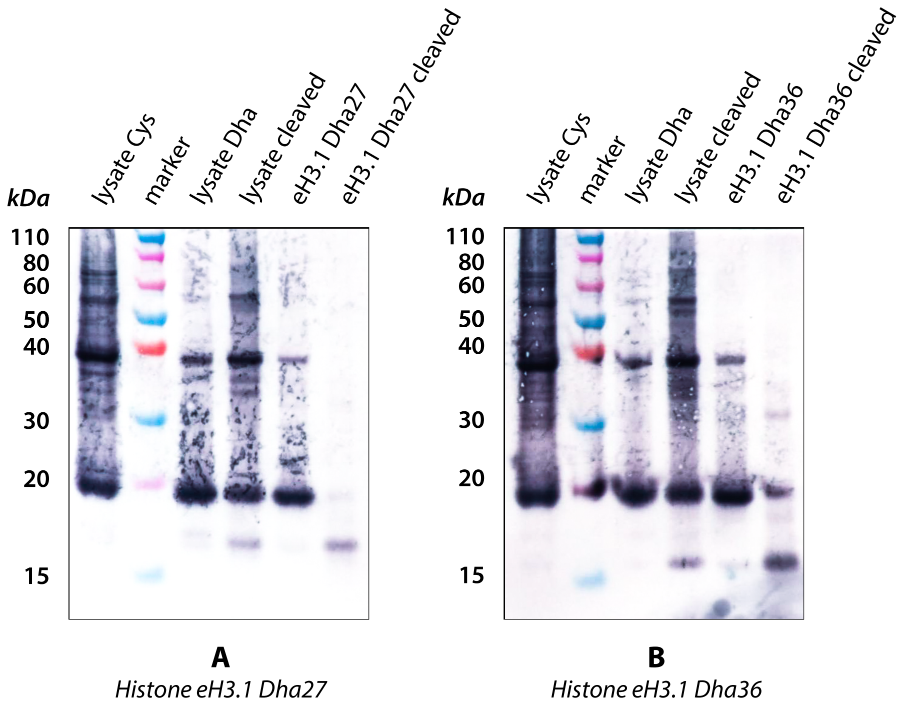

Supplementary Figure S10: Western blot analysis of cleavage reaction of crude *E. coli* lysate containing overexpressed Histone eH3.1 K27Dha confirms in-lysate cleavage. (**A**) and eH3.1 K36Dha (**B**). Conditions as follows: Lysate Cys – lysate only; lysate Dha – lysate post Dha formation with DTT and DBHDA; lysate cleaved – Dha lysate after treatment with 100 mM $B_2(OH)_4$ and 25 mM sodium ascorbate at 25°C for 20 h; eH3.1 Dha27 and eH3.1 Dha36 – purified Dha mutant; eH3.1 Dha27 cleaved and eH3.1 Dha36 cleaved – purified Dha mutant after treatment with 100 mM $B_2(OH)_4$ and 25 mM sodium ascorbate at 25°C for 20 h. Western blot analysis was conducted using a primary mouse anti-HA monoclonal antibody.

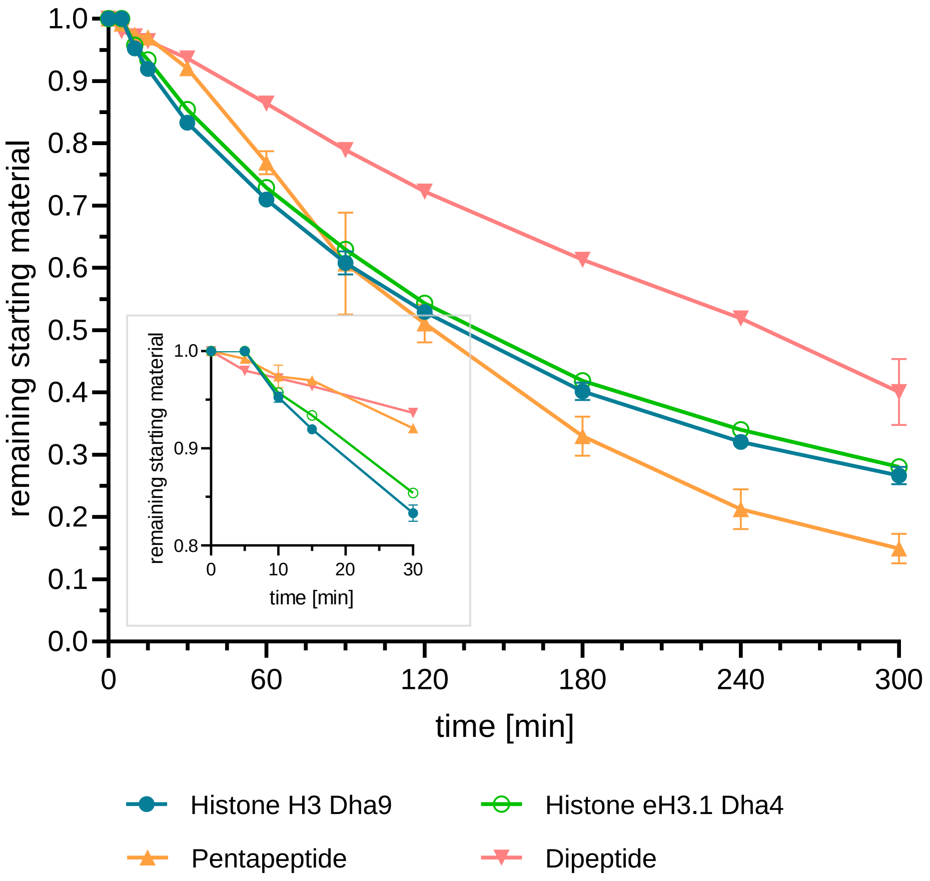

Supplementary Figure S11: Time-course comparison of the cleavage for Histone H3 Dha9 (teal), Histone eH3.1 Dha4 (green), pentapeptide Ac-Arg-Thr-Dha-Gln-Thr-NH_2 (4) (yellow) and dipeptide Ac-Gly-Dha-NHBn (1) (red) shows rate enhancement for protein over peptide. Reactions were conducted at 50 μM substrate concentration with $B_2(OH)_4$ (10 mM, 200 equiv), sodium ascorbate (2.5 mM, 50 equiv) in NaP_i buffer (10 mM, pH 8). The proportion of remaining starting material was monitored by LCMS. All reactions were conducted in triplicate (error bars depict S.D.).

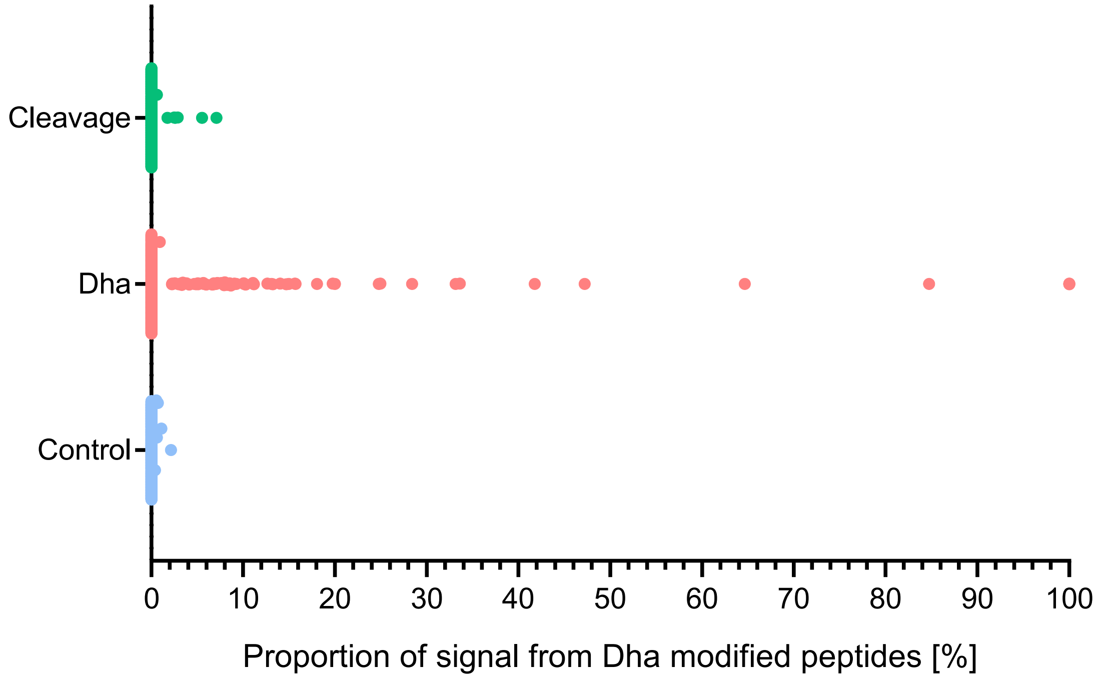

Supplementary Figure S12: Determination of Dha-containing peptides in *E. coli* BL21(DE3) lysate confirms depletion of Dha under cleavage conditions. Control: untreated lysate, Dha: after treatment with DBHDA under Dha formation conditions, Cleavage: after Dha formation and cleavage. Each point represents one peptide and the proportion of its Dha mutant is depicted on the x-axis. For this analysis, only Cys-containing peptides identified in all three samples were taken into consideration and not identified peptides were excluded. A total of n=276 Cys-containing peptides were identified, Dha mutants were identified for n=64 peptides after DBHDA (2,5-dibromohexanediamide) treatment, for n=8 peptides after cleavage and for n=8 peptides in the control sample.

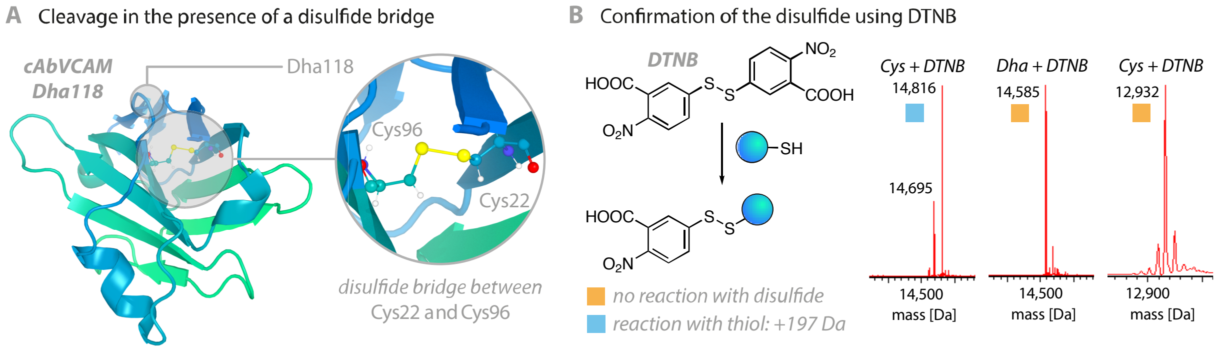

Supplementary Figure S13: Dha formation and cleavage on nanobody cAbVCAM proceeds without cleavage of the disulfide bond between Cys22 and Cys96. (**A**) as confirmed by LCMS analysis after incubation with DTNB (Ellman's reagent). A crystal structure of the structurally similar nanobody H4-H11 was used for the graphical representation (PDB ID 6ZBP).

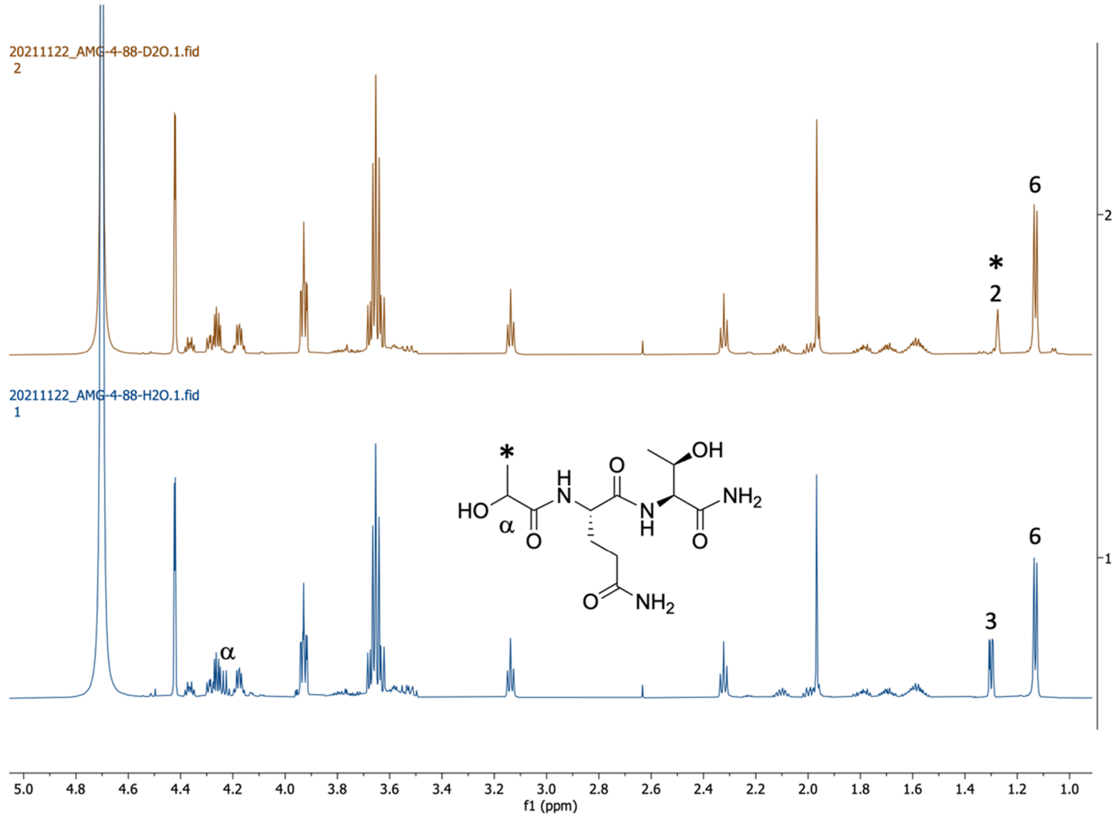

Supplementary Figure S14: NMR analysis of the crude cleavage reaction of pentapeptide 4 (Ac-Arg-Thr-Dha-Gln-Thr-NH_2) in D_2O confirms deuterium incorporation. The reaction was conducted in D_2O (top) and H_2O (bottom). Clearly observed are two D atoms incorporated into the *C*-terminal fragment as indicated by the * (methyl proton) and α (alpha proton). Note the difference in integrals between sample run in D_2O and H_2O.

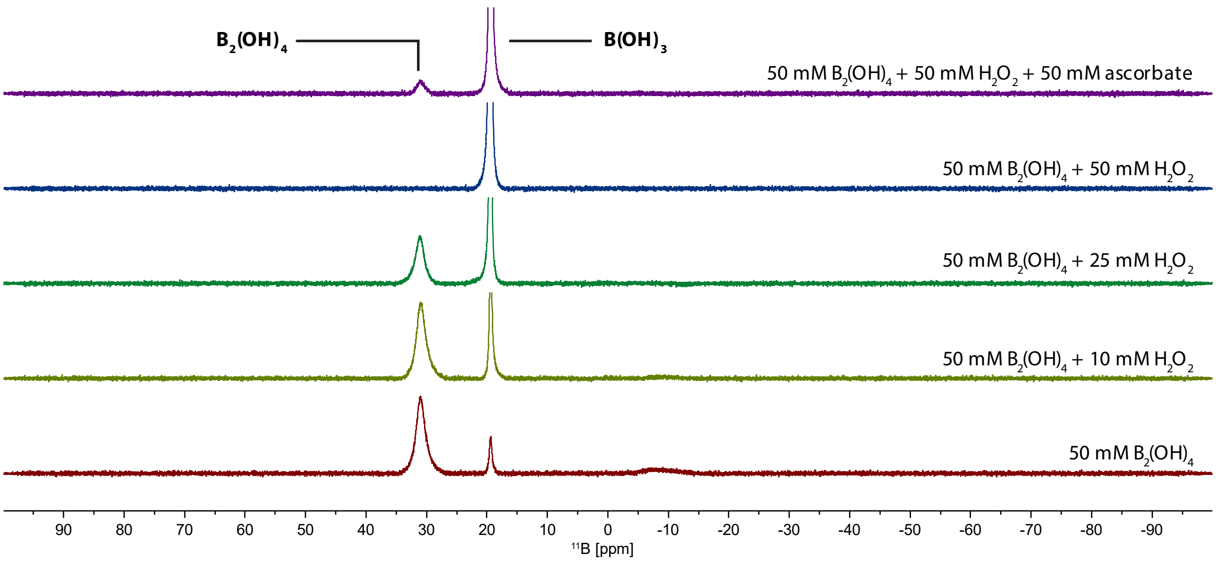

Supplementary Figure S15: Sodium ascorbate protects tetrahydroxydiboron ($B_2(OH)_4$) from oxidative decomposition to boric acid ($B(OH)_3$). Reactions were conducted at 50 mM $B_2(OH)_4$ in the presence (top) or absence or 50 mM sodium ascorbate at varying concentrations of H_2O_2.

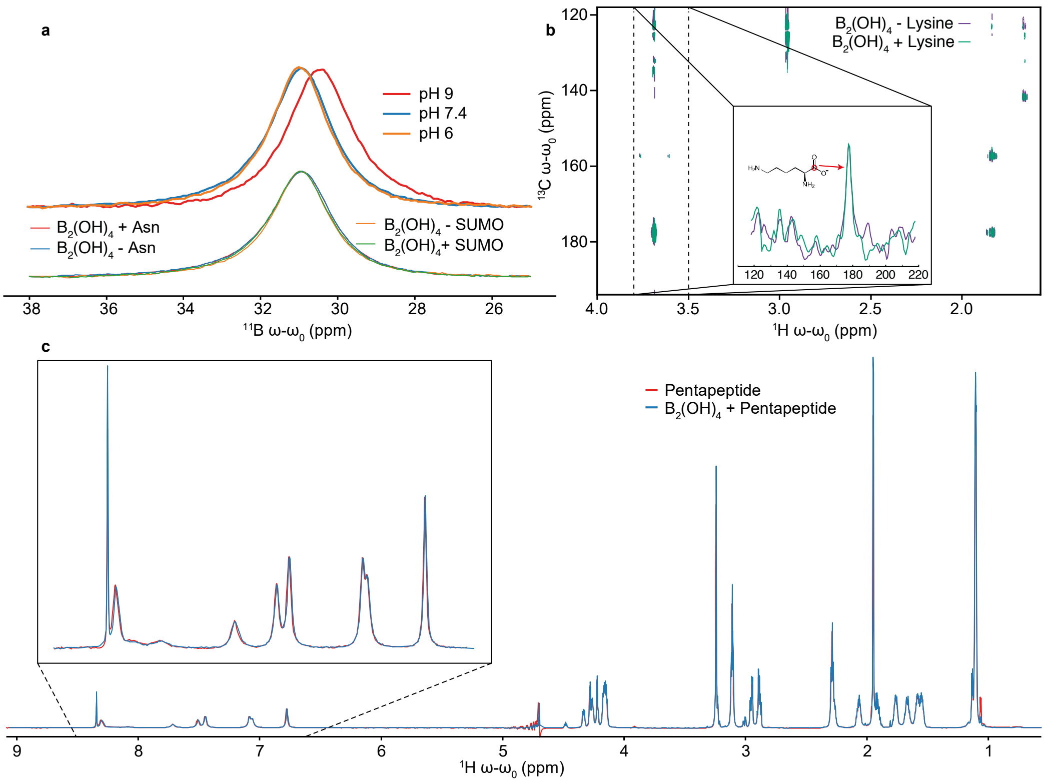

Supplementary Figure S16: Steady state titrations of tetrahydroxyboron with proteins, amino acids and polypeptide substrates enables an estimation of their interaction strength. To investigate the interaction between tetrahydroxyboron and peptides, a series of NMR titrations were performed, observing the ^{11}B, ^{1}H and ^{13}C chemical shifts where appropriate (**a**,**b**,**c**). The titrations with Asn and Lys had the amino acids at 50 mM, with equimolar tetrahydroxyboron (**a**,**b**). The polypeptide experiments were performed at 5 mM, and the SUMO concentration was in the presence of 50 mM tetrahydroxyboron (**c**). The experiments were performed in PBS with pH adjusted to 7.4 unless otherwise state. No significant difference was observed in either peak positions or intensities of resonances from polypeptide (**c**) or lysine (**b**) in the presence and absence of tetrahydroxyboron. This includes resonances from carbonyls detected via proton detected HMBC experiments (**b**), known to have an affinity for borates(9). Moreover, the resonances of boron were unaffected by the addition of substrates Asn and SUMO (**a**), though significant variation as observed when the pH was varied (**a**). The lack of a change in peak positions and intensities in these spectra places limits on the strength of the interaction between boron and Lewis bases with proteins and polypeptides to a K_D >> 100 mM, 10-fold larger than the highest concentration used.

Taken together, we have a range of data from which we can ascertain approximate rate constants for the association of boron with Lewis bases on proteins and polypeptides, by estimating k_{on} to be 10^{-2} $M^{-1}s^{-1}$ from the macroscopic chemical kinetics, and k_{off} to be 10^{3} s^{-1}. The details of the calculations are described below. The rate constants that characterise the interaction between boron and polypeptide substrates need to explain the 3 sets of experimental observations 1) macroscopic chemical kinetics

show the reaction to be second order with respect to boron and peptide, and that the reaction is ca. 80% complete after 3 hours with boron concentration of 10 mM and polypeptide concentration 50 μM (Supplementary Figure S11). Assuming irreversible second order kinetics, k_{on} for the association needs to be ca. 10^{-2} $M^{-1}s^{-1}$. 2) the observation from our previous work that shows covalently attaching a borate to a flexible protein results in transient interactions between the boron and adjacent carbonyl groups in a manner that leads to 'intermediate chemical exchange', which results in loss of HN signal intensity in HSQC NMR spectra*(9)*. The effective concentration for a single boron in the context of the chain can be estimated to be $C = 1/VN_A$ where N_A is Avogadro's constant and V is the volume occupied by the boron in dm^{-3}. Taking the volume occupied by the 30 residue chain to be approximate a box of 1 nm^3 *(9,67)* we obtain an effective concentration of Boron as ca. 1 M, a typical value expected for the enhanced 'effective concentration' when a substrate is covalently locked inside the active site of an enzyme. With these approximations, even with the high effective concentration, the exchange rate for the reaction will be $k_{ex} = k_{on}C + k_{off}$ which because k_{on} is small, is equal to k_{off}. The condition for intermediate chemical exchange, where intensity is lost from an NMR spectrum but the locations of observed resonances do not change, is that the ratio of k_{ex} to the chemical shift difference caused by the process is approximately 1, and cannot exceed 10 otherwise the exchange will be 'fast' and we will not observe losses in signal intensity. For typical changes in chemical shift expected in proteins due to conformational rearrangement, this typically requires millisecond dynamics and an exchange rate in the vicinity of 10^3 s^{-1}*(67)*. This allows us to estimate k_{off} to be $10^3 s^{-1}$. 3) no direct interaction was observed in the steady state titrations (**a-c**), putting the K_D limit to be >100 mM. Increasing or decreasing either of these values by an order of magnitude leads to very different predictions in the three experiments described. Increasing k_{on} for example leads to the chemical reaction completing one order magnitude more quickly than observed and vice versa, and so this value is most safely estimated from the combined observations and chemical kinetics presented here. Increasing or decreasing k_{off} by an order of magnitude, depending on the specific chemical shift differences incurred during the reaction could still explain the DICE data. These values are challenging to determine experimentally. Altering k_{off} in this matter alters the K_D by an order of magnitude, which still leaves the expected value to be greater than the limit inferred from the titrations. These results illustrate that a weak, transient interaction between boron and Lewis bases on a substrate are in principle sufficient to drive reactions to completion on an hour timescale, as observed. A more detailed characterisation of the rates constants is beyond the scope of this manuscript.

2 Supplementary Tables

Supplementary Table S1: Cleavage conditions, conversions and site-accessibility.

Entry	Method	Cleavage site	typical conditions	type	Notes	Ref
**1**	**TEV**	–ENLYFQ↓G/S–	50 mM TRIS-HCl, 0.5 mM EDTA, 1 mM DTT, 30°C	enzymatic		*(58, 68, 69)*
**2**	**Factor Xa**	–IE/NGR↓P2'–	20 mm TRIS-HCl, 1 mm CaCl2, 100 mm NaCl, pH 8.0, room temperature	enzymatic	unspecific cleavage possible	*(58, 70–72)*
**3**	**CNBr**	–P1-M↓P2'–	50 mg/mL CNBr in 70% formic acid, room temperature	chemical	harsh, denaturing conditions, highly toxic	*(31, 70)*
**4**	**Keto-ABNO**	–P1↓S-P2'–	CuI (5 equiv), bathophen salt (5 equiv), keto-ABNO (5 equiv), $NaNO_2$ (15 equiv), MeCN/water/AcOH (9/9/2), O_2 (1 atm), room temperature	chemical	harsh, denaturing conditions	*(35)*
**5**	**SNAC-tag**	–G↓SHHW–	0.1 M CHES, 0.1 M acetone oxime, 0.1 M NaCl, 1 mM $NiCl_2$, pH 8.6, room temperature	chemical	feasible under native conditions	*(34)*
**6**	**2-Nitrophenylalanine**	2-Nitrophenylalanine	PBS, pH 7.4, UV irradiation (365 nm)	photochemical	low cleavage efficiency (~30%)	*(36)*
**7**	**BNPS-skatole**	–P1-W↓P2'–	BNPS-skatole (100 equiv) in aqueous acetic acid, aqueous 0.1% TFA or aqueous 0.1% TFA in EtOH, 47°C or room temperature	chemical	cleavage efficiencies can be low, harsh conditions	*(31, 73)*
**8**	**NTCB**	–P1-C↓P2'–	200 mM TRIS, 1 mM EDTA, 0.1% SDS, 4 mM NTCB, pH 8.0; then cleavage 50°C, pH 9.0	chemical	harsh conditions, incomplete cleavage, side reactions	*(28, 31, 74)*
**9**	**formic acid**	–D↓P–	70% aqueous formic acid, 37°C	chemical	harsh conditions, incomplete cleavage	*(31, 75)*
**10**	**hydroxylamine**	–N↓G–	1.8 M aqueous hydroxylamine, pH 9.0, 45°C	chemical	harsh conditions, incomplete cleavage	*(31)*

**11**	**DAPase**	N-terminal amino acid	20 mM NaP_i, 150 mM NaCl, pH 7.0	enzymatic	stops at K, R or P	*(76)*
**12**	**Diboron-ascorbate (this work)**	–P1↓Dha-P2'–	100 mM TRIS-HCl, 10 mM $B_2(OH)_4$, 2.5 mM sodium ascorbate, pH 8.0, room temperature	chemical	feasible under native conditions	
**13**	**Thrombin**	–LVPR↓GS–	50 mM TRIS-HCl, 150 mM NaCl, 2.5 mM $CaCl_2$, 10 µM heparin, pH 7.5, room temperature	enzymatic	unspecific cleavage possible	*(70, 71)*

Supplementary Table S2: Cleavage conditions, conversions and site-accessibility.

Entry	Protein	rSASA [%]†	Conditions	Yield [%]*
1	Histone H3 Dha9	42	23 h, rt	>95%
2	Histone H3 Dha10	79	24 h, rt	>95%
3	FLAG-Histone H3 Dha4	61	3 h, rt	>95%
4	Histone eH3 Dha27	94	18 h, rt	>95%
5	Histone eH3 Dha36	89	18 h, rt	>95%
6	Histone H4 Dha16	69	17 h, rt	>95%
7	Npβ Dha61	26	24 h, 45°C	85%
8	preSUMO1 Dha51	2	48 h, rt	14%#
9	Annexin V Dha316	12	3 h, 45°C	39%
10	preSUMO1 Dha97	103	18 h, rt	>90%
11	cAbVCAM Dha118	N/A	18 h, rt	55%

†Relative solvent-accessible surface areas (rASA) were calculated for the respective Cys mutants relative to the accessibility of Cys within the tripeptide Gly-Cys-Gly. Calculations were conducted using the Lee & Richards algorithm, NACCESS atom radii and a probe size of 1.4 Å size as implemented in FreeSASA.*(77, 78)* Previously published crystal structures with the following PDB IDs were used: 1KX5 (Histones), 2J8K (Npβ), 1A5R (preSUMO1) and 1HVD (Annexin V). No rSASA was calculated for cAbVCAM as no crystal structure was available. Mutation of the residue of interest to Cys was conducted in PyMol 2.4.0. *Yields were determined by LC-MS unless stated otherwise. #Yield determined by SDS-PAGE and Coomassie staining via densitometry

3 Supplementary Methods

3.1 Proteolytic Peptide Mapping

ArgC digestion

50 µg of protein sample were dissolved in freshly prepared denaturing buffer (50 µL, 4 M urea, 100 mM ammonium bicarbonate) and incubated at room temperature for 10 min. The sample was diluted to a final concentration of 2 M urea using a 100 mM ammonium bicarbonate solution. $CaCl_2$ was added to a final concentration of 2 mM. Activation solution (50 mM DTT, 5 mM EDTA in water) was added (1:10, v/v) before ArgC (1 µg, sequencing grade, Promega, Catalogue No. V1881) was added. The sample was shaken at 37°C (800 rpm) for 20 hours. The reaction was quenched by addition of formic acid (5%, v/v) and centrifuged (30 min, 14,000 rpm, 4°C). The sample was desalted using a C_{18} resin containing tip and lyophilized in a protein LoBind microcentrifuge tube (Eppendorf, Catalogue No. 0030108116).

Data Acquisition

Resulting peptides were separated using a nano-flow reversed-phase liquid chromatography Ultimate 3000 UHPLC system (Thermo Fisher Scientific) and analysed using a Q-Exactive Hybrid Quadrupole-Orbitrap mass spectrometer (Thermo Fischer Scientific). Data acquisition was performed by the Advanced Proteomics Facility, University of Oxford.

Data Analysis

Data analysis was performed with PEAKS Studio 8.5 (Bioinformatics Solutions Inc.). The data was searched against the given protein sequence. The precursor mass tolerance was set to 15 ppm and the fragment mass tolerance was set to 0.5 Da. A maximum number of 3 missed cleavages and non-specific cleavage at one end of the peptide were specified. Methionine oxidation (+15.99 Da), deamidation (+0.98 Da, asparagine, glutamine) and carbamylation (+43.01 Da, lysine) were set as variable post-translational modifications. Additionally, *N*-terminal lactoylation (+72.02 Da) was included in the search. A maximum of 4 variable modifications was defined and a FDR of 1% on peptide level was applied.

3.2 Determination of Conversion via Protein LCMS

Protein samples were analysed on Waters Xevo G2-S QTof or Waters Xevo G2-XS QTof mass spectrometers equipped with a Waters Acquity UPLC. Separation was achieved using a Thermo Scientific™ ProSwift™ RP-2H monolithic column (4.6 × 50 mm) using water + 0.1% formic acid (Solvent A) and acetonitrile + 0.1% formic acid (Solvent B) as mobile phase at a flow rate of 0.3 mL/min and running a 10 min linear gradient as follows: 5% Solvent B for 1 min, 5% to 95% Solvent B over 6 min, 95% to 5% Solvent B over 1 min, 5% Solvent B for 2 min. Spectra were deconvoluted using MassLynx 4.1 (Waters) and the "MaxEnt1" deconvolution algorithm with the following settings: resolution: 1.0 Da/channel; damage model: uniform Gaussian; width at half height: 0.4 Da; minimum intensity ratios: left 33%, right 33%; iterate to convergence. Conversions were calculated from peak intensities.

3.3 Reaction Optimization

3.3.1 Cleavage Additive Screening

Lyophilized Histone H3 Dha10 was dissolved in milli-Q water at 1.0 mg/mL. Stock solutions of tetrahydroxydiboron (5.9 mg in 1000 µL), TEMPOL (4-Hydroxy-2,2,6,6-tetramethylpiperidinyloxyl, 14.2 mg in 1000 µL), iron(II) sulfate heptahydrate (22.8 mg in 1000 µL) and sodium ascorbate (16.3 mg in 1000 µL) in milli-Q water were prepared freshly directly before the reactions were conducted.

50 µL of Histone H3 Dha10 (50 µg, 3.29 nmol, 1.00 equiv) were incubated at room temperature with 2 µL of additive (164 nmol, 50 equiv) and 10 µL of tetrahydroxydiboron (657 nmol, 200 equiv). Samples were taken after 30 min, 60 min and 180 min and directly analysed by LC-MS. Cleavage and oxidation were determined from relative intensities after deconvolution of the corresponding mass spectra.

3.3.2 Cleavage Boron Reagent Screening

Lyophilized Histone H3 Dha10 was dissolved in milli-Q water at 1.0 mg/mL. Stock solutions of tetrahydroxydiboron (5.9 mg in 1000 µL), *bis*(catecholato)diboron (15.6 mg in 1000 µL), $NaBH_4$ (2.5 mg in 1000 µL), $NaBH_3CN$ (4.1 mg in 1000 µL) and sodium ascorbate (13.0 mg in 1000 µL) in milli-Q water were prepared. A stock solution of *bis*(pinacolato)diboron (16.7 mg in 1000 µL) was prepared in DMF. Stock solutions of catecholborane (14.1 µL, 15.8 mg in 1000 µL) and pinacolborane (19.1 µL, 16.8 mg in 1000 µL) were prepared in dry MeCN. All stock solutions were prepared freshly before use.

40 µL of Histone H3 Dha10 (40 µg, 2.63 nmol) were treated with sodium ascorbate (2 µL, 131 nmol, 50 equiv) followed by boron reagent (8 µL, 526 nmol, 200 equiv) and incubated for 3 hours at room temperature. The samples were analysed by LC-MS and cleavage was determined from relative intensities after deconvolution of the corresponding mass spectra.

3.3.3 Antioxidant Concentration Screening

Lyophilized Histone H3 Dha10 was dissolved in milli-Q water at 1.0 mg/mL. Stock solutions of tetrahydroxydiboron (5.9 mg in 1000 µL) and sodium ascorbate (32.5 mg in 500 µL) in milli-Q water were prepared freshly directly before the reactions were conducted.

50 µL of Histone H3 Dha10 (50 µg, 3.29 nmol, 1.00 equiv) were incubated at room temperature with varying concentrations of sodium ascorbate (10 µL, 3.29 mmol, 1000 equiv; 5 µL, 1.64 mmol, 500 equiv; 1.0 µL, 329 µmol, 100 equiv; 0.1 µL, 16.4 µmol, 10 equiv) and 10 µL of tetrahydroxydiboron (657 nmol, 200 equiv). Samples were taken after 30 min, 90 min, 180 min and 360 min and directly analysed by LC-MS. Cleavage and oxidation were determined from relative intensities after deconvolution of the corresponding mass spectra.

3.3.4 Temperature Screening

Lyophilized Histone H3 Dha10 was dissolved in milli-Q water at 1.0 mg/mL. Stock solutions of tetrahydroxydiboron (5.9 mg in 1000 µL) and sodium ascorbate (13.0 mg in 1000 µL) in milli-Q water were prepared.

40 µL of Histone H3 Dha10 (40 µg, 2.63 nmol) were treated with sodium ascorbate (2 µL, 131 nmol, 50 equiv) followed by tetrahydroxydiboron (8 µL, 526 nmol, 200 equiv) and incubated at 4°C, room temperature or 37°C. Samples were taken after 30 min, 60 min, 90 min, 120 min, 180 min, 360 min, 24 h and 4 days. No further samples were taken once the reaction was found to have gone to completion. The samples were analysed by LC-MS and cleavage was determined from relative intensities after deconvolution of the corresponding mass spectra.

3.3.5 Buffer Screening

Lyophilized Histone H3 Dha10 was dissolved in milli-Q water at 4.0 mg/mL. A 2× stock of each buffer was prepared. Stock solutions of tetrahydroxydiboron (5.9 mg in 1000 µL) and sodium ascorbate (13.0 mg in 1000 µL) in milli-Q water were prepared.

A mixture of 2× buffer (20 µL), Histone H3 Dha10 (10 µL, 40 µg, 2.63 nmol), sodium ascorbate (2 µL, 131 nmol, 50 equiv) and tetrahydroxydiboron (8 µL, 526 nmol, 200 equiv)

was incubated at room temperature for 3 hours. A sample was taken and directly analysed by LC-MS. Cleavage was determined from relative intensities after deconvolution of the corresponding mass spectrum.

3.4 Deuteration Experiments

3.4.1 LC-MS Analysis of Protein Deuteration

LC-MS analysis of protein deuteration was conducted on high resolution QTof mass spectrometers (Waters Xevo G2-S or Waters Xevo G2-XS). All samples were measured and analysed in triplicate. Samples were analysed in MassLynx 4.1 (Waters) using the integrated MaxEnt1 algorithm for deconvolution. For deuteration experiments, the mass range was set to 5000 Da to 25000 Da, the mass resolution was set to 0.04 Da/channel, the damage model was set to uniform Gaussian with 0.4 Da width at half-height. Minimum intensity ratios were set to 33% left and 33% right.

3.4.2 Deuterative Cleavage

Lyophilized Histone H3 Dha10 was dissolved in D_2O at 1.0 mg/mL. Stock solutions of $B_2(OD)_4$ (5.9 mg in 1000 µL) and sodium ascorbate (13.0 mg in 1000 µL) in D_2O were prepared.

40 µL of Histone H3 Dha10 (40 µg, 2.63 nmol) were treated with sodium ascorbate (2 µL, 131 nmol, 50 equiv) followed by $B_2(OD)_4$ (8 µL, 526 nmol, 200 equiv) and incubated room temperature for 3 hours. A control experiment was conducted under identical conditions using H_2O instead of D_2O. Samples were taken and directly analysed by LC-MS as described above.

3.4.3 Transfer Deuteration under Anaerobic Conditions

Lyophilized Histone H3 Dha10 was dissolved in D_2O at 1.0 mg/mL and transferred to a nitrogen filled glove box (2.1 ppm O_2). The sample was degassed overnight. Fresh stock solutions of $B_2(OD)_4$ (5.9 mg in 1000 µL) and sodium ascorbate (13.0 mg in 1000 µL) in D_2O were prepared in the glove box.

100 µL of Histone H3 Dha10 (100 µg, 6.57 nmol) were treated with sodium ascorbate (5 µL, 329 nmol, 50 equiv) followed by $B_2(OD)_4$ (20 µL, 1.31 µmol, 200 equiv) and incubated room temperature for 3 hours. A control experiment was conducted under identical conditions using H_2O instead of D_2O. Samples were taken and directly analysed by LC-MS as described above.

3.4.4 Deuteration under Aerobic Conditions in the Presence of TEMPOL

Lyophilized Histone H3 Dha10 was dissolved in D_2O at 1.0 mg/mL. Stock solutions of $B_2(OD)_4$ (5.9 mg in 1000 µL) and TEMPOL (11.3 mg in 1000 µL) in D_2O were prepared.

100 µL of Histone H3 Dha10 (100 µg, 6.57 nmol) were treated with TEMPOL (5 µL, 329 nmol, 50 equiv) followed by $B_2(OD)_4$ (20 µL, 1.31 µmol, 200 equiv) and incubated room temperature for 24 hours. A control experiment was conducted under identical conditions using H_2O instead of D_2O. Samples were taken and directly analysed by LC-MS as described above.

3.5 SUMOylation

SUMOylation was conducted using a commercially available SUMOylation assay kit (Abcam, Catalogue No. ab139470) following the manufacturer's instructions. 5 µL (approx. 0.3 mg/mL) of a solution of chemically cleaved preSUMO1 in TRIS buffer (100 mM, pH 8.0) were treated with milli-Q water (10 µL), 10× SUMOylation buffer (2 µL), 20× Mg-ATP (1 µL), 20× SUMO E1 (1 µL) and 20× RanGAP1 (1 µL). Control reactions containing no Mg-ATP or 20× SUMO1 (1 µL) were conducted. The amount of milli-Q water used was adjusted to achieve a total reaction volume of 20 µL. The reaction mixtures were incubated at 37°C for 60 minutes before being quenched by addition of 5× Laemmli buffer (5 µL) and heating to 95°C (10 min).

The reactions were analysed by SDS-PAGE (MES running buffer, 10% bis-TRIS precast gel, 200 V, 40 min, room temperature) and Western blot. Western blot analysis was conducted using the primary rabbit anti-SUMO1 polyclonal antibody supplied with the SUMOylation kit (1:1000 dilution) and a secondary goat anti-rabbit IgG–alkaline phosphatase fusion (1:1000 dilution; Sigma Aldrich, Catalogue No. A3687). NBT/BCIP substrate solution (Thermo Scientific, Catalogue No. 34042) was used to visualize proteins. Blocking was conducted using 5% (w/v) skimmed milk powder in TBS-T buffer.

3.6 Protein Scope

3.6.1 Protein Expression

3.6.1.1 Histone H3 S10C

Protein Sequence

```
1          11         21         31         41         51
ARTKQTARKC TGGKAPRKQL ATKAARKSAP ATGGVKKPHR YRPGTVALRE IRRYQKSTEL
61         71         81         91         101        111
LIRKLPFQRL VREIAQDFKT DLRFQSSAVM ALQEASEAYL VALFEDTNLA AIHAKRVTIM
121        131
PKDIQLARRI RGERA
```

Formula: $C_{670}H_{1131}N_{215}O_{185}S_3$
Calculated molecular weight: 15254.9 g/mol
Extinction coefficient $\varepsilon = 4470\ M^{-1}\ cm^{-1}$

Plasmid amplification

The plasmid was transformed into Agilent XL10-Gold® ultra-competent *E. coli* (Agilent) according to the manufacturer's transformation protocol and grown on agar plates (supplemented with ampicillin) over night. A single colony was selected and transferred to 5 mL of LB media containing 100 µg/mL ampicillin. The culture was incubated at 37 °C for 14 h and the cell pellet was harvested. Plasmid purification was conducted using a QIAprep Spin Miniprep kit and following the manufacturer's instructions.

Transformation and Expression

Protein Expression

BL21(DE3)pLysS competent *E. coli* (Agilent) were transformed with the plasmid following the manufacturer's instructions and grown on agar plates (supplemented with 100 µg/mL carbenicillin and 34 µg/mL chloramphenicol) over night. Single colonies were selected and transferred to 5 mL of LB media (containing 100 µg/mL carbenicillin and 34 µg/mL chloramphenicol) each. The starter cultures were incubated at 37 °C for 16 h. One starter culture was prepared for each 450 mL of expression media. Eight 2.5 L Erlenmeyer flasks charged with

450 mL of LB media supplemented with carbenicillin (at final concentration of 100 µg/mL) and chloramphenicol (at a final concentration of 34 µg/mL) were inoculated with the starter cultures. The bacterial cultures were incubated at 37 °C with vigorous shaking (250 rpm) an optical density value (OD_{600}) of 0.6 – 0.8 was observed. Protein expression was induced by addition of 1 mM IPTG and the culture was further incubated at 37 °C for 2 h. The cells were harvested by centrifugation (7,500 rpm, 20 min, 4 °C). The cell pellet was suspended in 25 mL of lysis buffer (50 mM TRIS base, 100 mM NaCl, 1 mM EDTA, 5 mM BME, pH 7.5) containing a tablet of cOmplete™ mini EDTA-free protease inhibitor cocktail. The sample was frozen in liquid nitrogen and stored at −80 °C until purification.

Protein Purification

The samples were thawed on ice and DNase I (1 mg) was added. The cell suspension was then sonicated on ice (5 cycles, 40% amplitude, 30 sec. on, 60 sec. off). The samples were centrifuged (20,000 rpm, 20 min, 4 °C) and the supernatant was discarded. The cell pellet was re-suspended in 20 mL of TW buffer (50 mM TRIS base, 100 mM NaCl, 1 mM EDTA, 5 mM BME, 1% Triton X-100, pH 7.5) using a mixer and sonicated on ice (2 cycles, 40% amplitude, 30 sec. on, 60 sec. off). The mixture was centrifuged (20,000 rpm, 10 min, 4 °C) and the supernatant was discarded. The pellet was again suspended in 20 mL of TW buffer, centrifuged (20,000 rpm, 10 min, 4 °C) and the supernatant was discarded. Washingn was repeated one more time with TW buffer and one more time with wash buffer (50 mM TRIS base, 100 mM NaCl, 1 mM EDTA, 5 mM BME, pH 7.5). The pellet was incubated with 1 mL of DMSO at room temperature for 10 min before 10 mL of unfolding buffer (7 M Gdn·HCl, 10 mM TRIS base, 1 mM EDTA, 10 mM DTT, 1 mM benzamidine) were added. The mixture was shaken at room temperature for 1 h and centrifuged (20,000 rpm, 10 min, 4 °C). The supernatant was purified by FPLC using a size-exclusion column running with SAU-100 buffer (7 M urea, 20 mM NaOAc, 100 mM NaCl, 1 mM EDTA, 5 mM BME, pH 5.2). The Histone H3 containing fractions were further purified by anion exchange chromatography using a linear gradient from SAU-100 buffer to SAU-1000 buffer (7 M urea, 20 mM NaOAc, 1000 mM NaCl, 1 mM EDTA, 5 mM BME, pH 5.2). The clean fractions were pooled together and dialyzed four times against 4 L of water containing 2 mM BME. The protein solution was lyophilized yielding 230 mg of Histone H3 S10C (64 mg/L expression volume). The lyophilized protein stored at −20 °C until needed.

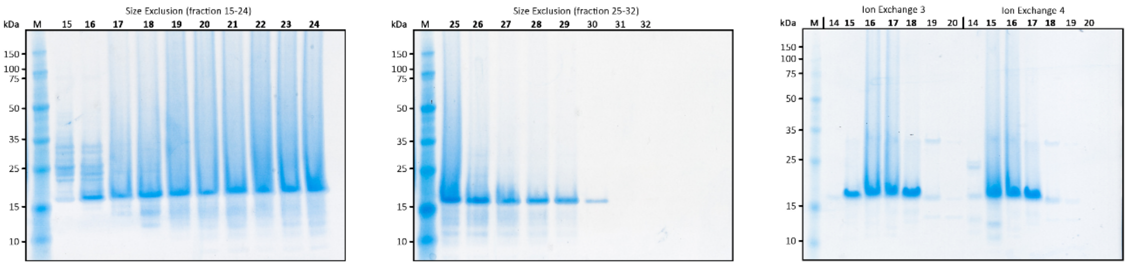

Supplementary Figure S17: Purification and analysis of purified fractions of Histone H3 S10C via SDS-PAGE and coomassie staining shows expected bands. M = marker.

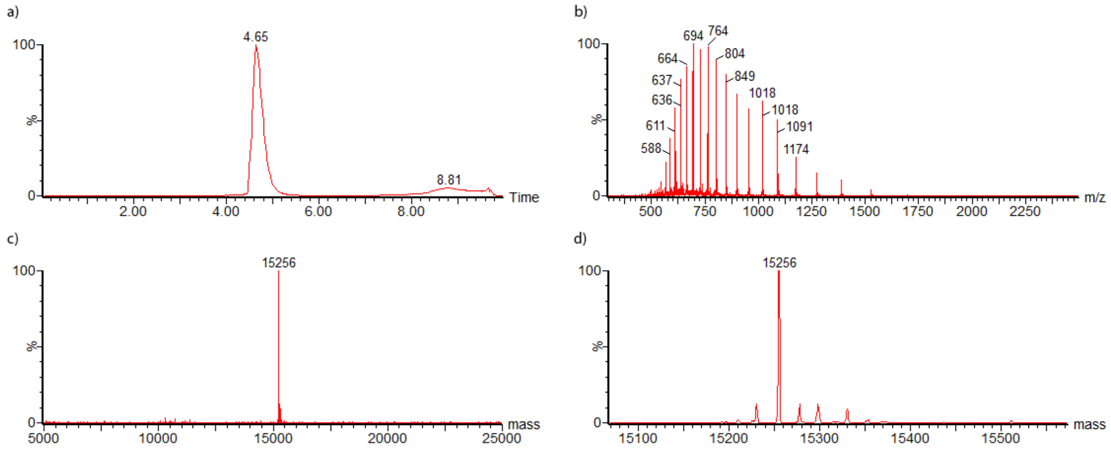

Supplementary Figure S18: LCMS analysis of Histone H3 S10C shows expected protein mass. a) total ion chromatogram b) ion series c) deconvoluted spectrum d) magnification of the major peak**;** calculated mass: 15255 g/mol; observed masses: 15256 g/mol.

3.6.1.2 Histone H3 K9C

Protein Sequence

```
1          11         21         31         41         51
ARTKQTARCS TGGKAPRKQL ATKAARKSAP ATGGVKKPHR YRPGTVALRE IRRYQKSTEL
61         71         81         91         101        111
LIRKLPFQRL VREIAQDFKT DLRFQSSAVM ALQEASEAYL VALFEDTNLA AIHAKRVTIM
121        131
PKDIQLARRI RGERA
```

Formula: $C_{667}H_{1124}N_{214}O_{186}S_3$

Calculated molecular weight: 15213.8 g/mol

Extinction coefficient ε = 4470 M^{-1} cm^{-1}

Plasmid amplification

The plasmid was transformed into Agilent XL10-Gold® ultra-competent *E. coli* (Agilent) according to the manufacturer's transformation protocol and grown on agar plates (supplemented with ampicillin) over night. A single colony was selected and transferred to 5 mL of LB media containing 100 µg/mL ampicillin. The culture was incubated at 37 °C for 14 h and the cell pellet was harvested. Plasmid purification was conducted using a QIAprep Spin Miniprep kit and following the manufacturer's instructions.

Transformation and Expression

Protein Expression

BL21(DE3)pLysS competent *E. coli* (Agilent) were transformed with the plasmid following the manufacturer's instructions and grown on agar plates (supplemented with 100 µg/mL carbenicillin and 34 µg/mL chloramphenicol) over night. Single colonies were selected and transferred to 5 mL of LB media (containing 100 µg/mL carbenicillin and 34 µg/mL chloramphenicol) each. The starter cultures were incubated at 37 °C for 16 h. One starter culture was prepared for each 450 mL of expression media. Eight 2.5 L Erlenmeyer flasks charged with 450 mL of LB media supplemented with carbenicillin (at final concentration of 100 µg/mL) and chloramphenicol (at a final concentration of 34 µg/mL) were inoculated with the starter cultures. The bacterial cultures were incubated at 37 °C with vigorous shaking (250 rpm) an optical density value (OD_{600}) of 0.6 – 0.8 was observed. Protein expression was induced by

addition of 1 mM IPTG and the culture was further incubated at 37 °C for 2 h. The cells were harvested by centrifugation (7,500 rpm, 20 min, 4 °C). The cell pellet was suspended in 25 mL of lysis buffer (50 mM TRIS base, 100 mM NaCl, 1 mM EDTA, 5 mM BME, pH 7.5) containing a tablet of cOmplete™ mini EDTA-free protease inhibitor cocktail. The sample was frozen in liquid nitrogen and stored at −80 °C until purification.

Protein Purification

The samples were thawed on ice and DNase I (1 mg) was added. The cell suspension was then sonicated on ice (5 cycles, 40% amplitude, 30 sec. on, 60 sec. off). The samples were centrifuged (20,000 rpm, 20 min, 4 °C) and the supernatant was discarded. The cell pellet was re-suspended in 20 mL of TW buffer (50 mM TRIS base, 100 mM NaCl, 1 mM EDTA, 5 mM BME, 1% Triton X-100, pH 7.5) using a mixer and sonicated on ice (2 cycles, 40% amplitude, 30 sec. on, 60 sec. off). The mixture was centrifuged (20,000 rpm, 10 min, 4 °C) and the supernatant was discarded. The pellet was again suspended in 20 mL of TW buffer, centrifuged (20,000 rpm, 10 min, 4 °C) and the supernatant was discarded. Washingn was repeated one more time with TW buffer and one more time with wash buffer (50 mM TRIS base, 100 mM NaCl, 1 mM EDTA, 5 mM BME, pH 7.5). The pellet was incubated with 1 mL of DMSO at room temperature for 10 min before 10 mL of unfolding buffer (7 M Gdn·HCl, 10 mM TRIS base, 1 mM EDTA, 10 mM DTT, 1 mM benzamidine) were added. The mixture was shaken at room temperature for 1 h and centrifuged (20,000 rpm, 10 min, 4 °C). The supernatant was purified by FPLC using a size-exclusion column running with SAU-100 buffer (7 M urea, 20 mM NaOAc, 100 mM NaCl, 1 mM EDTA, 5 mM BME, pH 5.2). The Histone H3 containing fractions were further purified by anion exchange chromatography using a linear gradient from SAU-100 buffer to SAU-1000 buffer (7 M urea, 20 mM NaOAc, 1000 mM NaCl, 1 mM EDTA, 5 mM BME, pH 5.2). The clean fractions were pooled together and dialyzed four times against 4 L of water containing 2 mM BME. The protein solution was lyophilized yielding 187 mg of Histone H3 K9C (52 mg/L expression volume). The lyophilized protein stored at −20 °C until needed.

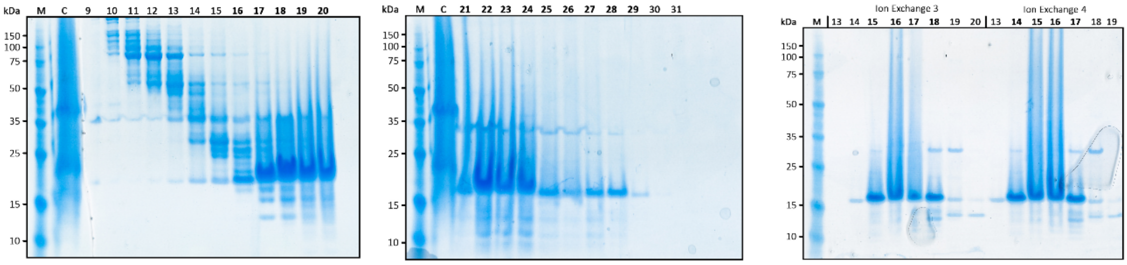

Supplementary Figure S19: Purification and analysis of purified fractions of Histone H3 S10C via SDS-PAGE and coomassie staining shows expected bands. M = marker.

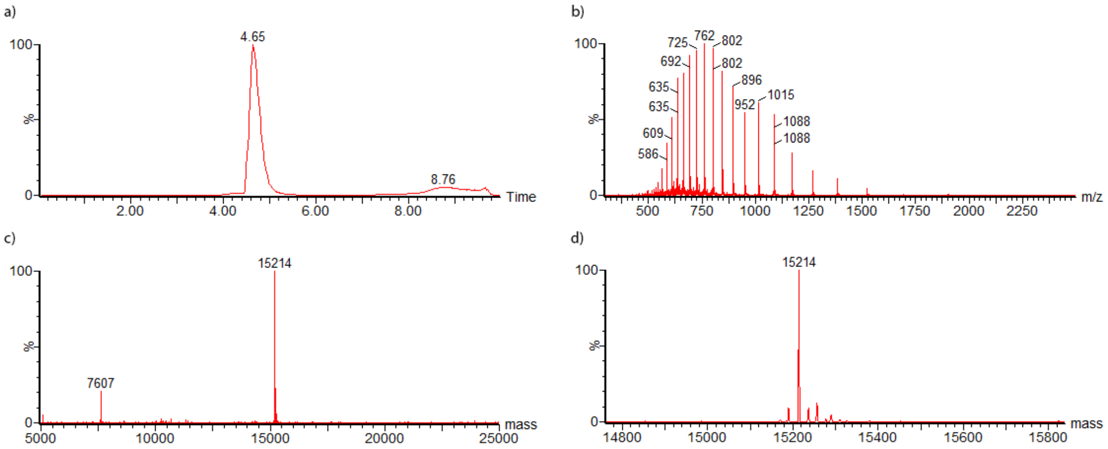

Supplementary Figure S20: LCMS analysis of Histone H3 K9C shows expected protein mass. a) total ion chromatogram b) ion series c) deconvoluted spectrum d) magnification of the major peak**;** calculated mass: 15214 g/mol; observed masses: 15214 g/mol.

3.6.1.3 Histone H4 K16C

```
        10         20         30         40         50         60
SGRGKGGKGL GKGGACRHRK VLRDNIQGIT KPAIRRLARR GGVKRISGLI YEETRGVLKV

        70         80         90        100
FLENVIRDAV TYTEHAKRKT VTAMDVVYAL KRQGRTLYGF GG
```

Formula: $C_{491}H_{830}N_{162}O_{134}S_2$

Calculated molecular weight: 11211.1 g/mol

Extinction coefficient $\varepsilon = 5960\ M^{-1}\ cm^{-1}$

Histone H4 K16C was expressed and purified following a previously published procedure.*(19)*

3.6.1.4 FLAG-Histone H3 K4C

```
        10         20         30         40         50         60
ARTCQTARKS TGGKAPRKQL ATKAARKSAP ATGGVKKPHR YRPGTVALRE IRRYQKSTEL

        70         80         90        100        110        120
LIRKLPFQRL VREIAQDFKT DLRFQSSAVM ALQEAAEAYL VGLFEDTNLA AIHAKRVTIM

       130        140        150
PKDIQLARRI RGERAGGDYK DDDDKSAAGG YPYDVPDYA
```

Formula: $C_{777}H_{1272}N_{240}O_{228}S_3$

Calculated molecular weight: 17720.3 g/mol

Extinction coefficient $\varepsilon = 10430\ M^{-1}\ cm^{-1}$

C-terminally FLAG-tagged Histone H3 K4C was expressed and purified following a previously published procedure.*(79)*

3.6.1.5 FLAG-Histone H3 K27C

```
        10         20         30         40         50         60
ARTKQTARKS TGGKAPRKQL ATKAARCSAP ATGGVKKPHR YRPGTVALRE IRRYQKSTEL

        70         80         90        100        110        120
LIRKLPFQRL VREIAQDFKT DLRFQSSAVM ALQEAAEAYL VGLFEDTNLA AIHAKRVTIM

       130        140        150
PKDIQLARRI RGERAGGDYK DDDDKSAAGG YPYDVPDYA
```

Formula: $C_{777}H_{1272}N_{240}O_{228}S_3$

Calculated molecular weight: 17720.3 g/mol

Extinction coefficient $\varepsilon = 10430\ M^{-1}\ cm^{-1}$

C-terminally FLAG-tagged Histone H3 K27C was expressed and purified following a previously published procedure.*(79)*

3.6.1.6 FLAG-Histone H3 K36C

```
        10         20         30         40         50         60
ARTKQTARKS TGGKAPRKQL ATKAARKSAP ATGGVCKPHR YRPGTVALRE IRRYQKSTEL

        70         80         90        100        110        120
LIRKLPFQRL VREIAQDFKT DLRFQSSAVM ALQEAAEAYL VGLFEDTNLA AIHAKRVTIM

       130        140        150
PKDIQLARRI RGERAGGDYK DDDDKSAAGG YPYDVPDYA
```

Formula: $C_{777}H_{1272}N_{240}O_{228}S_3$

Calculated molecular weight: 17720.3 g/mol

Extinction coefficient $\varepsilon = 10430\ M^{-1}\ cm^{-1}$

Genes for the human histone cysteine mutants were prepared as previously described.*(79)* The gene for the WT human histone eH3.1 (*C*-terminal FLAG-HA tag, C96A and C110A) was ordered from Thermofischer (GeneArt service) and cloned into the pET3d expression plasmid at the NcoI and BamHI restriction enzyme sites. Quickchange mutagenesis was performed per the manufacturer's instructions (QuikChange II Site-Directed Mutagenesis Kit, Agilent) to create the desired cysteine mutant at site K36. Expression and purification of the histone was carried out following exactly the same procedure as previously described for the other histones.*(79)*

3.6.1.7 Annexin V

Protein Sequence

```
1          11         21         31         41         51
AQVLRGTVTD FPGFDERADA ETLRKAMKGL GTDEESILTL LTSRSNAQRQ EISAAFKTLF
61         71         81         91         101        111
GRDLLDDLKS ELTGKFEKLI VALMKPSRLY DAYELKHALK GAGTNEKVLT EIIASRTPEE
121        131        141        151        161        171
LRAIKQVYEE EYGSSLEDDV VGDTSGYYQR MLVVLLQANR DPDAGIDEAQ VEQDAQALFQ
181        191        201        211        221        231
AGELKWGTDE EKFITIFGTR SVSHLRKVFD KYMTISGFQI EETIDRETSG NLEQLLLAVV
241        251        261        271        281        291
KSIRSIPAYL AETLYYAMKG AGTDDHTLIR VMVSRSEIDL FNIRKEFRKN FATSLYSMIK
301        311
GDTSGDYKKA LLLLCGEDD
```

Formula: $C_{1585}H_{2535}N_{423}O_{502}S_{8}$

Calculated molecular weight: 35805.6 g/mol

Extinction coefficient $\varepsilon = 23380\ M^{-1}\ cm^{-1}$

Plasmid amplification

The plasmid was transformed into Invitrogen One Shot TOP10 chemically competent *E. coli* according to the manufacturer's transformation protocol and grown on agar plates (supplemented with ampicillin) over night. A single colony was selected and transferred to 5 mL of LB media containing 100 µg/mL ampicillin. The culture was incubated at 37 °C for 14 h and the cell pellet was harvested. Plasmid purification was conducted using a QIAprep Spin Miniprep kit and following the manufacturer's instructions.

Transformation and Expression

Protein Expression

T7 Express Competent *E. coli* (New England BioLabs) were transformed with the plasmid following the manufacturer's instructions and grown on agar plates (supplemented with 100 µg/mL ampicillin) over night. Single colonies were selected and transferred to 10 mL of LB media (containing 100 µg/mL ampicillin) each. The starter culture was incubated at 37 °C for 15 h. One starter culture was prepared for every 500 mL of expression media. Four 2.5 L

Erlenmeyer flasks charged with 500 mL of LB media supplemented with carbenicillin (at final concentration of 100 µg/mL) were inoculated with the starter cultures. The bacterial cultures were incubated at 37 °C with vigorous shaking (250 rpm) an optical density value (OD_{600}) of 0.45 – 0.55 was observed. Protein expression was induced by addition of 1 mM IPTG and the culture was further incubated at 37 °C for 3 h. The cells were harvested by centrifugation (8,000 rpm, 10 min, 4 °C). The cell pellet was suspended in 60 mL of TBS buffer (50 mM TRIS base, 150 mM NaCl, pH 8.0) and re-pelleted by centrifugation (10,000 rpm, 10 min, 4 °C), then re-suspended in 60 mL of $CaCl_2$ buffer (50 mM TRIS base, 10 mM $CaCl_2$, pH 7.2) containing a tablet of cOmplete™ mini EDTA-free protease inhibitor cocktail. The samples were frozen in liquid nitrogen and stored at −80 °C until purification.

Protein Purification

The samples were thawed on ice and DNase I was added. The cell suspension was then sonicated on ice (5 cycles, 50% amplitude, 30 sec. on, 60 sec. off). The samples were centrifuged (18,000 rpm, 20 min, 4 °C) and the supernatant was discarded. The cell pellet was re-suspended in 40 mL of EDTA buffer (50 mM TRIS base, 20 mM EDTA, pH 7.2) and the cell debris was removed by centrifugation (18,000 rpm, 20 min, 4 °C). The annexin V containing supernatant was filtered through a 0.45 µm syringe filter and dialyzed against 3 × 4 L of TRIS buffer (20 mM TRIS base, pH 7.8). The dialyzed protein solution was purified by FPLC using an anion exchange column (10 mL) and running a gradient from TRIS buffer (20 mM TRIS base, pH 7.8) to NaCl buffer (20 mM TRIS base, 500 mM NaCl, pH 7.8). The purified protein was dialyzed against 3 × 4 L of NaP_i buffer (50 mM NaP_i, pH 8.0). The protein solution was concentrated using a VivaSpin 20 centrifugal concentrator (MWCO 10,000, 4,000 rpm, 10 min, 4 °C) to yield 10 mL of a protein solution. The protein concentration was determined to be 5.40 mg/mL using an A280 spectrophotometer. The expression yield was determined to be 27 mg/L. The protein solution was divided into aliquots, frozen in liquid nitrogen and stored at −80 °C until needed.

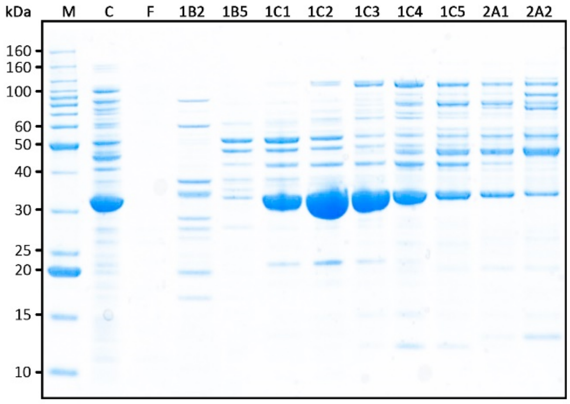

Supplementary Figure S21: Analysis of purified fractions of Annexin V via SDS-PAGE and coomassie staining shows expected bands. M = BenchMark™ Unstained Protein Ladder, C = crude, F = flow through.

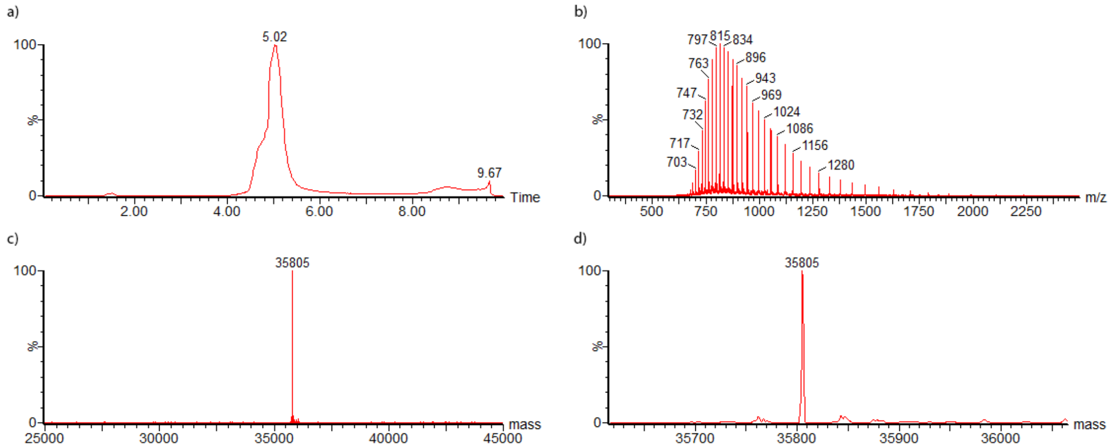

Supplementary Figure S22: LCMS analysis of Annexin V shows expected protein mass. a) total ion chromatogram b) ion series c) deconvoluted spectrum d) magnification of the major peak**;** calculated mass: 35806 g/mol; observed mass: 35805 g/mol.

3.6.1.8 Npβ C61

Protein Sequence

```
1          11         21         31         41         51
MFSSHHHHHH SSGLVPRGSH IDVGKLRQLY AAGERDFSIV DLRGAVLENI NLSGAILHGA
61         71         81         91         101        111
CLDEANLQQA NLSRADLSGA TLNGADLRGA NLSKADLSDA ILDNAILEGA ILDEAVLNQA
121        131        141        151        161        171
NLKAANLEQA ILSHANIREA DLSEANLEAA DLSGADLAIA DLHQANLHQA ALERANLTGA
181        191        201
NLEDANLEGT ILEGGNNNLA T
```

Formula: $C_{898}H_{1456}N_{276}O_{303}S_2$

Calculated molecular weight: 21031.2 g/mol

Extinction coefficient $\varepsilon = 1490\ M^{-1}\ cm^{-1}$

Plasmid amplification

The plasmid was transformed into Invitrogen One Shot TOP10 chemically competent *E. coli* according to the manufacturer's transformation protocol and grown on agar plates (supplemented with kanamycin) over night. A single colony was selected and transferred to 10 mL of LB media containing 50 µg/mL kanamycin. The culture was incubated at 37 °C for 14 h and the cell pellet was harvested. Plasmid purification was conducted using a QIAprep Spin Miniprep kit and following the manufacturer's instructions.

Transformation and Expression

Protein Expression

T7 Express Competent *E. coli* (New England BioLabs) were transformed with the plasmid following the manufacturer's instructions and grown on agar plates (supplemented with 50 µg/mL kanamycin) over night. Single colonies were selected and transferred to 10 mL of LB media (containing 50 µg/mL kanamycin) each. The starter culture was incubated at 37 °C for 15 h. One starter culture was prepared for every 900 mL of expression media. Two 2.5 L Erlenmeyer flasks charged with 900 mL of LB media supplemented with kanamycin (at final concentration of 30 µg/mL) were inoculated with the starter cultures. The bacterial cultures were incubated at 37 °C with vigorous shaking (250 rpm) an optical density value (OD_{600}) of

0.7 – 0.8 was observed. Protein expression was induced by addition of 1 mM IPTG and the culture was further incubated at 37 °C for 4 h. The cells were harvested by centrifugation (8,000 rpm, 15 min, 4 °C). The cell pellet was suspended in 25 mL of lysis buffer (20 mM imidazole, 20 mM TRIS base, 500 mM NaCl, 2 mM DTT, 5% glycerol, pH 7.8) containing half a tablet of cOmplete™ mini EDTA-free protease inhibitor cocktail. The sample was frozen in liquid nitrogen and stored at −80 °C until purification.

Protein Purification

The samples were thawed on ice and DNase I (2 mg) was added. The cell suspension was then sonicated on ice (10 cycles, 60% amplitude, 15 sec. on, 60 sec. off). The samples were centrifuged (18,000 rpm, 10 min, 4 °C) and the supernatant was filtered through a 0.45 µm syringe filter and purified via FPLC. The sample was loaded onto a 5 mL GE HisTrap™ HP column at a flow rate of 1 mL/min. The column was washed with 20 CV of buffer A (40 mM imidazole, 20 mM TRIS base, 500 mM NaCl, 2 mM DTT, 5% glycerol, pH 7.8) and the protein was eluted running a linear gradient of 20 CV to 100% buffer B (500 mM imidazole, 20 mM TRIS base, 500 mM NaCl, 2 mM DTT, 5% glycerol, pH 7.8). The fractions were analyzed by SDS-PAGE and clean fractions containing Npβ were pooled together yielding 60 mL of protein solution/ The purified protein solution was dialyzed against 3 × 4 L of NaP_i buffer (50 mM NaP_i, pH 7.8) and concentrated using four VivaSpin 20 centrifugal concentrators (MWCO 10,000, 4,000 rpm, 15 min, 4 °C) to yield 10.3 mL of a protein solution. The protein concentration was determined to be 5.48 mg/mL using an A280 spectrophotometer. The expression yield was determined to be 31 mg/L. The protein solution was divided into aliquots, frozen in liquid nitrogen and stored at −80 °C until needed.

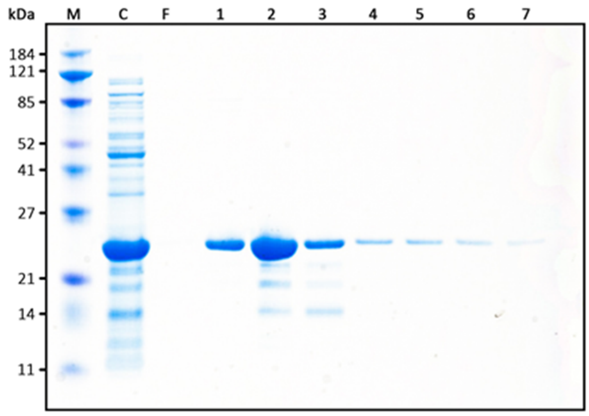

Supplementary Figure S23: Analysis of purified fractions of Npβ via SDS-PAGE and coomassie staining shows expected bands. M = ProSieve Color Protein Marker, C = crude lysate, F = flow through.

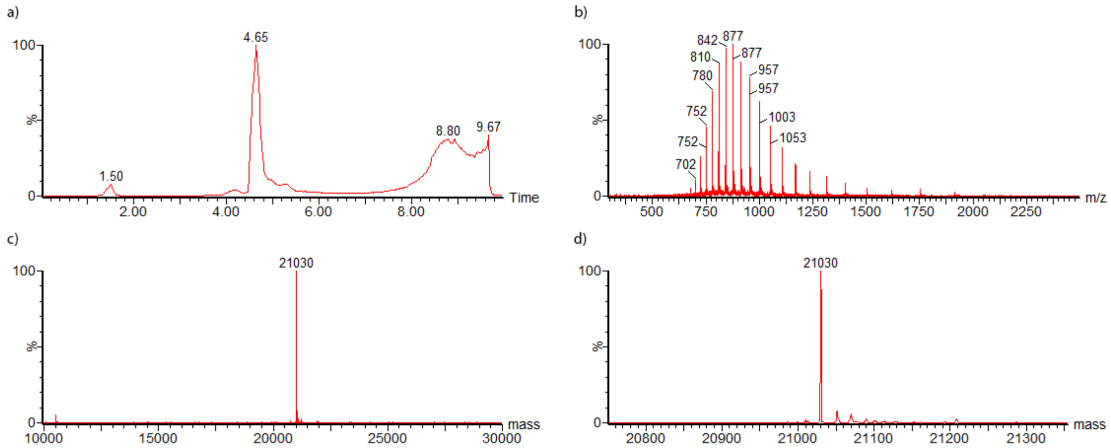

Supplementary Figure S24: LCMS analysis of Npβ shows expected protein mass. a) total ion chromatogram b) ion series c) deconvoluted spectrum d) magnification of the major peak; calculated mass: 21031 g/mol; observed mass: 21030 g/mol.

3.6.1.9 pre-SUMO1 C51

Protein Sequence

```
1          11         21         31         41         51
ADQEAKPSTE DLGDKKEGEY IKLKVIGQDS SEIHFKVKMT THLKKLKESY CQRQGVPMNS
61         71         81         91         101
LRFLFEGQRI ADNHTPKELG MEEEDVIEVY QEQTGGHSTV LEHHHHHH
```

Formula: $C_{543}H_{851}N_{157}O_{173}S_4$

Calculated molecular weight: 12474.9 g/mol

Extinction coefficient $\varepsilon = 4470\ M^{-1}\ cm^{-1}$

Plasmid amplification

A pET28a plasmid encoding the gene for pre-SUMO1 was a gift from Guy Salvesen distributed by Addgene (Addgene plasmid #25101) as an agar stab. Bacteria were streaked and grown on agar plates (supplemented with kanamycin) over night. A single colony was selected and transferred to 5 mL of LB media containing 50 µg/mL kanamycin. The culture was incubated at 37°C overnight and the cell pellet was harvested. Plasmid purification was conducted using a QIAprep Spin Miniprep kit (Qiagen) and following the manufacturer's instructions.

Transformation and Expression

Protein Expression

Chemically competent *E. coli* BL21(DE3) pLysS (Agilent) were transformed with pET28a-pre-SUMO1 and plated on agar plates (supplemented with 50 µg/mL kanamycin and 34 µg/mL chloramphenicol) according to the manufacturer's instructions. After over-night incubation, single colonies were transferred to 2 × 10 mL of freshly prepared LB media (supplemented with 50 µg/mL kanamycin and 34 µg/mL chloramphenicol). The starter cultures were incubated overnight and used to inoculate 2 × 900 mL of LB media (supplemented with 50 µg/mL kanamycin and 34 µg/mL chloramphenicol). The cultures were shaken at 37°C (250 rpm) until an optical density value (OD_{600}) of 0.8 – 1.0 was reached. Protein expression was induced by addition of 1 mM IPTG and the cultures were shaken for an additional 3 hours. The cells were harvested by centrifugation (8,500 rpm, 30 min, 4°C). The cell pellets were resuspended in 15 mL of lysis buffer (50 mM NaH_2PO_4, 10 mM imidazole, 300 mM NaCl, 10 mM BME, pH 8.0,

one tablet cOmplete™ Mini EDTA-free Protease Inhibitor Cocktail (Roche)), flash frozen in liquid nitrogen and stored at −80°C until purification.

Protein Purification

The samples were thawed and 1 mg of DNase I was added. The suspension was sonicated (10 cycles, 40% amplitude, 15 sec. on, 60 sec. off) and the cell debris was removed by centrifugation (20,000 rpm, 30 min, 4°C). The solution was loaded onto two HisTrap HP 5 mL columns (GE Healthcare) operated in series, the columns were washed with 20 CV of binding buffer (50 mM NaH_2PO_4, 20 mM imidazole, 500 mM NaCl, 10 mM BME, pH 8.0) and eluted with a 20 CV gradient 0-100% elution buffer (50 mM NaH_2PO_4, 500 mM imidazole, 500 mM NaCl, 10 mM BME, pH 8.0). The fractions were analysed by SDS-PAGE, clean fractions were pooled together and dialyzed twice against phosphate buffer supplemented with BME (50 mM NaH_2PO_4, 10 mM BME, pH 8.0) and once phosphate buffer (50 mM NaH_2PO_4, pH 8.0). The protein concentration was determined using an A280 spectrophotometer. The expression yield was determined to be 81.3 mg (45 mg/L expression volume). The protein solution was divided into aliquots, frozen in liquid nitrogen and stored at −80 °C until needed.

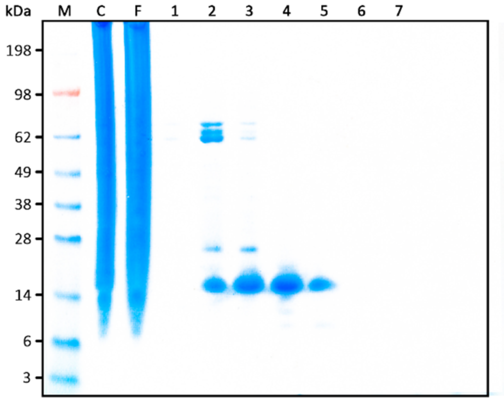

Supplementary Figure S25: Analysis of purified fractions via SDS-PAGE shows expected bands. Conditions: MES buffer, 200 V, 40 min, 10% Bis-TRIS gel and coomassie staining (M = SeeBlue® Plus2 Protein Standard, C = crude lysate, F = flow through).

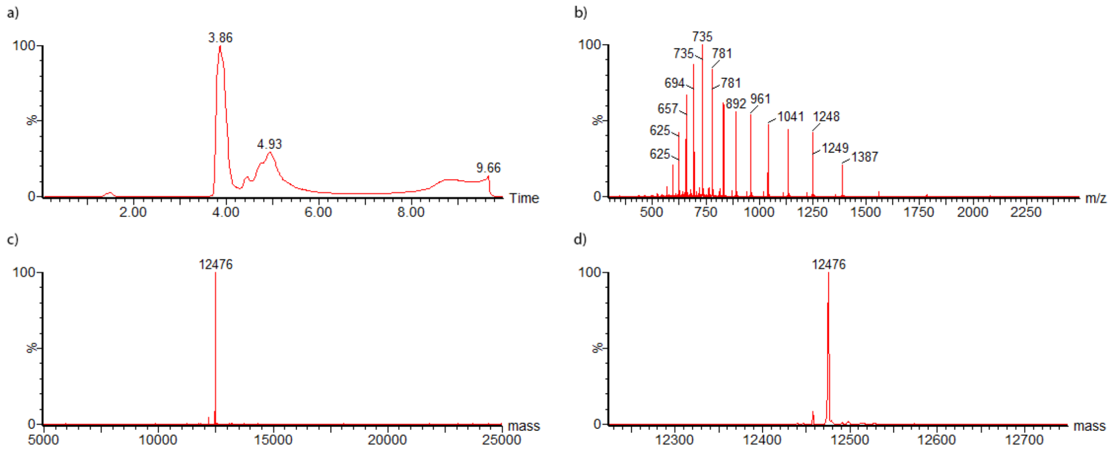

Supplementary Figure S26: LCMS analysis of preSUMO1 shows expected protein mass. a) total ion chromatogram b) ion series c) deconvoluted spectrum d) magnification of the major peak**;** calculated mass: 12475 g/mol; observed masses: 12476 g/mol.

3.6.1.10 preSUMO1 C51 C97

Protein Sequence

```
1          11         21         31         41         51
ADQEAKPSTE DLGDKKEGEY IKLKVIGQDS SEIHFKVKMT THLKKLKESY CQRQGVPMNS
61         71         81         91         101
LRFLFEGQRI ADNHTPKELG MEEEDVIEVY QEQTGGCSTV LEHHHHHH
```

Formula: $C_{540}H_{849}N_{155}O_{173}S_5$

Calculated molecular weight: 12440.9 g/mol

Extinction coefficient ε = 4470 M^{-1} cm^{-1}

Site-Directed Mutagenesis

Site-directed mutagenesis was conducted on a template pET28a plasmid encoding the gene for pre-SUMO1 (received as a gift from Guy Salvesen and distributed by Addgene; Addgene plasmid #25101). Site-directed mutagenesis was performed using the QuikChange II XL site-directed mutagenesis kit (Agilent, Catalogue No. 200521-5) following the manufacturer's instructions. The following primers (purchased from Merck Life Science UK) were used:

**Forward primer**	5'-TCAGGAACAAACGGGAGGTTGTTCAACAGTTCTCGAGCAC-3'
**Reverse primer**	5'-GTGCTCGAGAACTGTTGAACAACCTCCCGTTTGTTCCTGA-3'

The reaction mixture was transformed into One Shot™ MAX Efficiency™ DH5α-T1^R chemically competent cells (Invitrogen, Catalogue No. 12297016) and grown on agar plates (supplemented with kanamycin at 50 µg/mL) over night. Single colonies were picked and grown in 5 mL of LB media shaking (225 rpm) at 37°C for 18 hours. The cells were pelleted by centrifugation (8,000 rpm, 15 min) and the plasmid was extracted and purified using a QIAprep Spin Miniprep kit (Qiagen, Catalogue No. 27104) according to the manufacturer's instructions.

The presence of the desired mutation was confirmed by Sanger sequencing (Source BioScience, Cambridge, UK) using T7 forward and reverse primers.

5’-ATGGCTGACCAGGAGGCAAAACCATCAACTGAGGACTTGGGGGATAAGAAGGAAGGTGAATATAT
TAAACTCAAAGTCATTGGACAGGATAGCAGTGAGATTCACTTCAAAGTGAAAATGACAACACATCTCA
AGAAACTCAAAGAATCATACTGTCAAAGACAGGGTGTTCCAATGAATTCACTCAGGTTTCTCTTTGAG
GGTCAGAGAATTGCTGATAATCATACTCCAAAAGAACTGGGAATGGAGGAAGAAGATGTGATTGAAGT
TTATCAGGAACAAACGGGAGGT**TGT**TCAACAGTTCTCGAGCACCACCACCACCACCAC-3’

Transformation and Expression

Protein Expression

Chemically competent *E. coli* BL21(DE3) pLysS (Agilent) were transformed with the mutated pET28a-pre-SUMO1 C51 C97 plasmid and plated on agar plates (supplemented with 50 µg/mL kanamycin and 34 µg/mL chloramphenicol) according to the manufacturer’s instructions. After over-night incubation, single colonies were transferred to 2 × 10 mL of freshly prepared LB media (supplemented with 50 µg/mL kanamycin and 34 µg/mL chloramphenicol). The starter cultures were incubated overnight and used to inoculate 2 × 500 mL of LB media (supplemented with 50 µg/mL kanamycin and 34 µg/mL chloramphenicol). The cultures were shaken at 37°C (250 rpm) until an optical density value (OD_{600}) of 0.8 – 1.0 was reached. Protein expression was induced by addition of 1 mM IPTG and the cultures were shaken for an additional 3 hours. The cells were harvested by centrifugation (8,500 rpm, 30 min, 4°C). The cell pellets were resuspended in 15 mL of lysis buffer (50 mM NaH_2PO_4, 20 mM imidazole, 500 mM NaCl, 10 mM BME, pH 8.0, one tablet cOmplete™ Mini EDTA-free Protease Inhibitor Cocktail (Roche)).

Protein Purification

1 mg of DNase I was added to the resuspended cell pellet. The suspension was sonicated (10 cycles, 40% amplitude, 15 sec. on, 60 sec. off) and the cell debris was removed by centrifugation (20,000 rpm, 30 min, 4°C). The solution was loaded onto a HisTrap HP 5 mL column (GE Healthcare), the column was washed with 10 CV of binding buffer (50 mM NaH_2PO_4, 20 mM imidazole, 500 mM NaCl, 10 mM BME, pH 8.0) and eluted with elution buffer (50 mM NaH_2PO_4, 500 mM imidazole, 500 mM NaCl, 10 mM BME, pH 8.0). The fractions were analysed by SDS-PAGE, clean fractions were pooled together and dialyzed twice against milli-Q water supplemented with BME (500 µL/L). The protein concentration was determined using an A280 spectrophotometer. The expression yield was determined to be 25.8 mg. The protein solution was lyophilized and stored at −80 °C until needed.

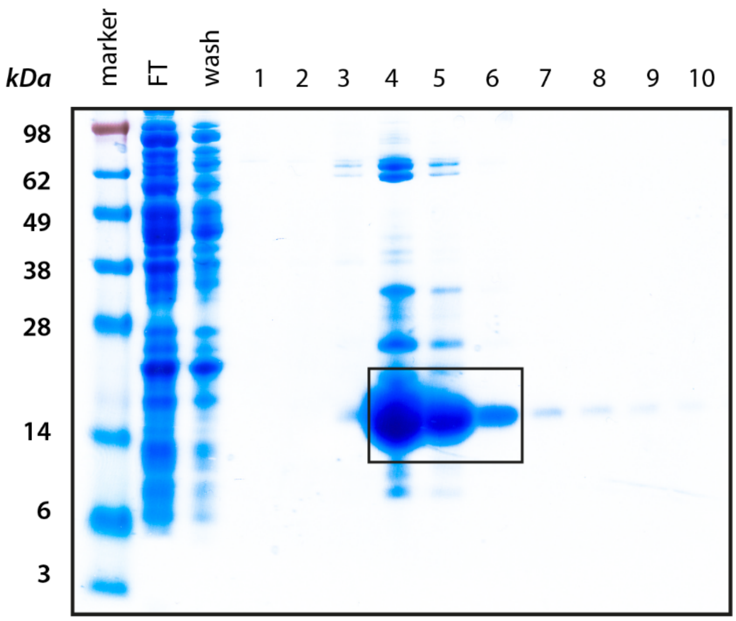

Supplementary Figure S27: Analysis of purified fractions via SDS-PAGE shows expected bands. Conditions: MES buffer, 200 V, 50 min, 4-12% Bis-TRIS gel and coomassie staining (M = SeeBlue® Plus2 Protein Standard, FT = flow through).

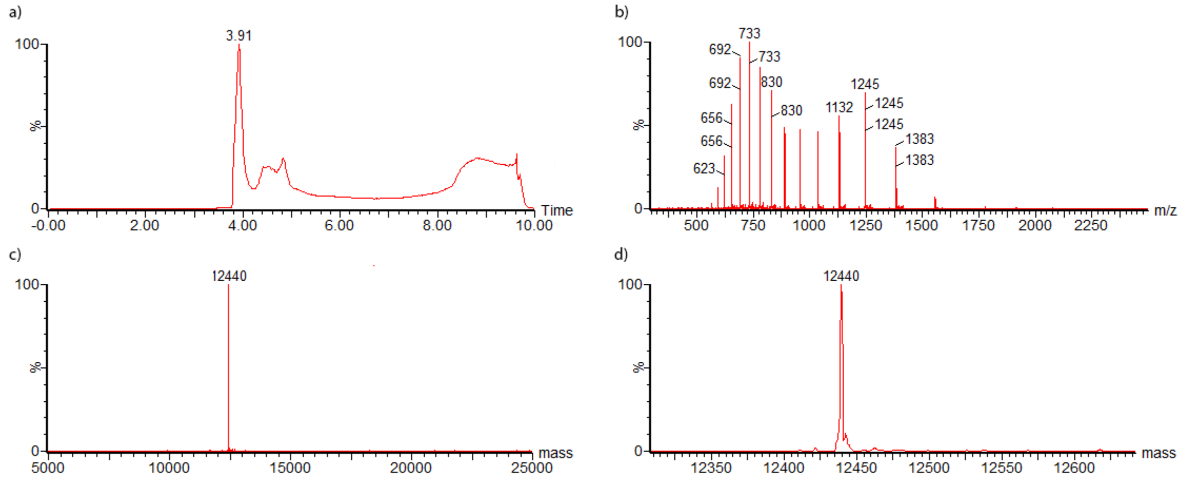

Supplementary Figure S28: LCMS analysis of preSUMO1 C51 C97 shows expected protein mass. a) total ion chromatogram b) ion series c) deconvoluted spectrum d) magnification of the major peak**;** calculated mass: 12441 g/mol; observed mass: 12440 g/mol.

3.6.1.11 cAbVCAM Q1N Q118C

Protein Sequence

```
1          11         21         31         41         51
NVQLQESGGG SVQTGGSLRL SCAASGYTNS IMYMAWFRQA PGKKREGVAA IRFPDDSAYY
61         71         81         91         101        111
AGSVKGRFTI SHDNAKNTVY LQMNNLNPED TAMYYCAARS SPYSFAWNDP SNYNYWGCGT
121         131
QVTVSSHHHH HH
```

Formula: $C_{639}H_{943}N_{187}O_{197}S_7$

Calculated molecular weight: 14621.1 g/mol

Extinction coefficient ε = 31525 M^{-1} cm^{-1}

Site-Directed Mutagenesis

Site-directed mutagenesis was conducted on a template pHEN4 plasmid encoding the gene for cAbVCAM1-5 (received as a gift from Prof. Nick Devoogdt). Site-directed mutagenesis was performed using the QuikChange II XL site directed mutagenesis kit (Agilent, Catalogue No. 200521-5) following the manufacturer's instructions. The following primers (purchased from Merck Life Science UK) were used:

Mutation 1 - Substitution of glutamine-118 for cysteine

Forward Primer 5'cccagtaactataactactggggctgcgggacccaggtcaccgtctcc3'

Reverse Primer 3'ggagacggtgacctgggtcccgcagccccagtagttatagttactggg5'

Mutation 2- Substitution of glutamine-1 for asparagine

Forward Primer 5'ctagcggcccagccggccatggccaacgtgcagctgcaggagtctggg3'

Reverse Primer 3'cccagactcctgcagctgcacgttggccatggccggctgggccgctag5'

The reaction mixture was transformed into XL10-Gold Ultracompetent Cells (Agilent, Catalogue NO. 200315) and grown on agar plates (supplemented with ampicillin at 100 µg/mL) over night. Single colonies were picked and grown in 5 mL of LB media shaking (225 rpm) at 37 °C for 12 h. The cells were pelleted by centrifugation (8,000 rpm, 5 min) and the plasmid was extracted and purified using a QIAprep Spin Miniprep kit (Qiagen, Catalogue No. 27104) according to the manufacturer's instructions.

The presence of the desired mutation was confirmed by Sanger sequencing (Source BioScience, Cambridge, UK) using T7 forward and reverse primers.

5’**aac**gtgcagctgcaggagtctgggggaggctcggtccagactggagggtctctgagactctcctgcgcagcctctggata
caccaatagtatcatgtacatggcctggttccgccaggctccagggaagaagcgcgagggggtcgcagctataagatttccc
gatgatagtgcctattatgccggctccgtgaagggccgattcaccatttcccacgacaacgccaagaacacggtgtatctgc
aaatgaacaacctgaatcctgaggacactgccatgtactactgtgcagcgcggtcgtcgccgtacagttttgcctggaacga
ccccagtaactataactactggggc**tgc**gggacccaggtcaccgtctcctcacaccaccatcaccatcac3’

Protein Expression and Purification

Protein Expression

Chemically competent WK6 *E.coli* (received as a gift from Prof. Ray Owens) were transformed with the mutated cAbVCAM Q1N Q118C plasmid by incubation on ice for 30 min, followed by heat-shock at 42 °C for 45 s, addition of SOC medium (180 µL), and incubation at 37 °C for 1 h with shaking (225 rpm). The cells were plated on agar plates (supplemented with ampicillin (100 µg/mL)) and grown at 37 °C for 16 h. Single colonies were transferred to 2 x 15 mL of freshly prepared LB media (supplemented with ampicillin (100 µg/mL)). The starter cultures were incubated at 37 °C for 16 h and 1 mL of the culture was used to inoculate 4 x 1 L Terrific Broth (TB) media (supplemented with ampicillin (100 µg/mL), 0.1 % glucose, and 2 mM $MgCl_2$). The cultures were shaken at 37 °C (180 rpm) until an optical density value (OD_{600}) of 0.6 – 0.9 was reached. Protein expression was induced by addition of 1 mM IPTG and the cultures were shaken at 28 °C (180 rpm) overnight. The cells were harvested by centrifugation (11,300 x g, 8 min, 4 °C) and stored at –80°C until purification.

Protein Purification

A single cell pellet was thawed on ice and mixed with 40 mL of TES lysis buffer (0.2 M Tris pH 7.8, 0.5 mM EDTA, 0.5 M Sucrose) containing one pre-dissolved cOmplete protease inhibitor mix tablet (EDTA free, Roche). The cell pellet was vortexed until bacterial clumps were not visible and then shaken via end-over-end mixing for 30 minutes at 4 ºC. 2 mg of DNase I were added and the mixture was further shaken via end-over-end mixing for 2 hours at 4 ºC. The lysate was centrifuged at 4°C and 22000×g for 15 min.

Supernatant was filtered through 0.2µm syringe filter and loaded to a pre-equilibrated HisTrap HP 5mL column (GE Healthcare, 2.5 mL/min), using a 50mL superloop (GE Healthcare). The protein was eluted running a stepwise gradient of 30 CV to 100% buffer B (10 CV Buffer A, 2 CV 5% Buffer B, 2 CV 7% Buffer B, 2 CV 10% Buffer B, 2 CV 20% Buffer B, 2 CV 35% Buffer B, 8 CV 100% Buffer B). The fractions were analyzed by SDS-PAGE and clean fractions containing protein were combined. The protein fractions were buffer exchanged to 50mM Na_2PO_4, pH 8 using an Amicon® Ultra-15 Centrifugal Filter Unit and samples were flash-frozen in liquid nitrogen and stored at -80°C. Protein expression yield was measured after the final buffer exchange and was measured at 11.2 mg/L.

Buffer A - 20 mM Tris-HCl, 15 mM imidazole, 500 mM NaCl, 0.05% (v/v) β-mercaptoethanol, pH 7.8

Buffer B - 20 mM Tris-HCl, 500 mM imidazole, 500 mM NaCl, 0.05% (v/v) β-mercaptoethanol, pH 7.8

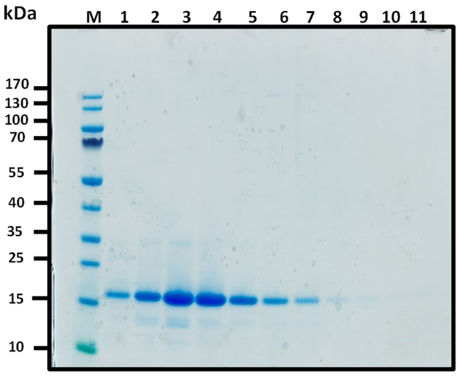

Supplementary Figure S29: Analysis of purified fractions of cAbVCAM Q1N Q118C via SDS-PAGE shows expected bands. Conditions: MES buffer, 200V, 40 min, 10% bis-TRIS gel) and coomassie staining (M = Perfect Protein™ Marker, Lane 1-7 = Pure protein)

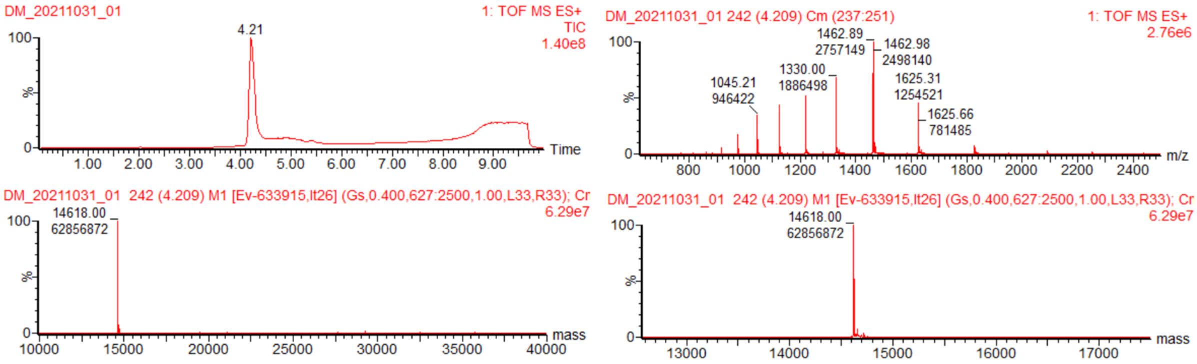

Supplementary Figure S30: LCMS analysis of cAbVCAM Q1N Q118C shows expected protein mass. a) total ion chromatogram b) ion series c) deconvoluted spectrum d) magnification of the major peak**;** calculated mass: 14619 g/mol; observed mass: 14618 g/mol.

3.6.2 Preparation of Dehydroalanine Protein Substrates

3.6.2.1 Histone H3 S10Dha

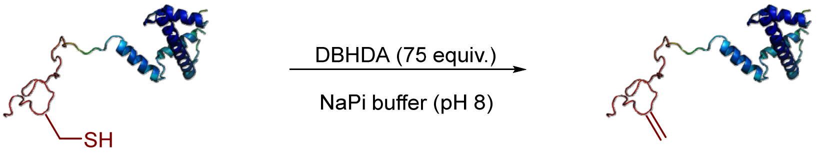

Histone H3 S10C **Histone H3 S10Dha**

Histone H3 S10C (11.4 mg, 0.75 µmol, 1.00 equiv.) and DTT (30 mg, 194 µmol, 260 equiv.) were dissolved in 500 µL of NaP_i buffer (100 mM, 3 M Gdn·HCl, pH 8.0) by sonication. The solution was incubated at room temperature for 1 h, before it was desalted by passing through a GE MiniTrap G-25 desalting column pre-equilibrated with NaP_i buffer (100 mM, 3 M Gdn·HCl, pH 8.0) according to the manufacturer's instructions. To the solution was added a solution of DBHDA (2,5-dibromohexanediamide) (18.0 mg, 59.8 µmol, 80 equiv.) in DMF (100 µL). The mixture was vortexed and gently shaken at room temperature for 75 min. until no starting material could be detected anymore by LCMS. The temperature was raised to 37 °C and the reaction mixture was shaken for an additional 2.5 h. The protein was desalted by passing through a GE MiniTrap G-25 column pre-equilibrated with NaP_i buffer (100 mM, 3 M Gdn·HCl, pH 8.0) to yield 2.00 mL of a protein solution with a concentration of 4.58 mg/mL as determined by A280 spectrophotometry (9.16 mg, 0.60 µmol, 80%).

The sample was aliquoted, frozen in liquid nitrogen and stored at −80°C.

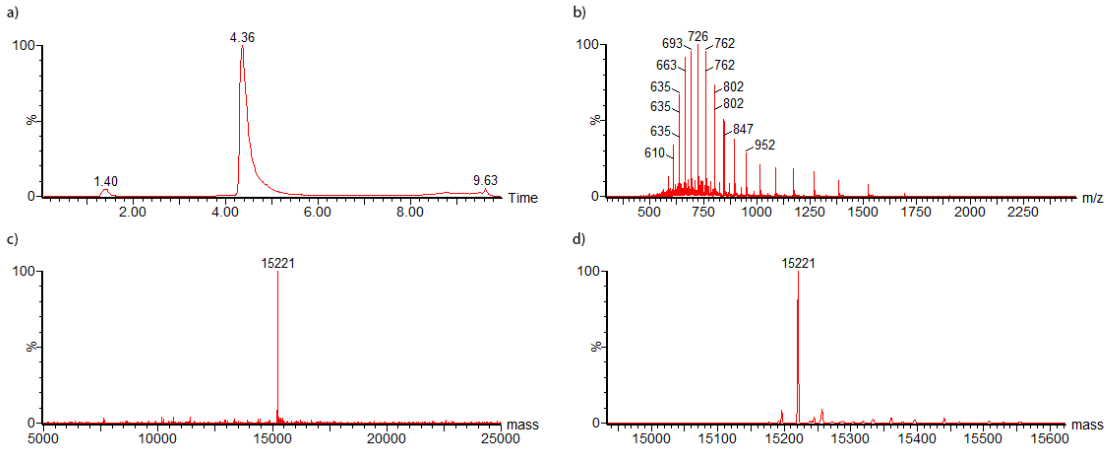

Supplementary Figure S31: LCMS analysis of Histone H3 S10Dha shows expected protein mass. a) total ion chromatogram b) ion series c) deconvoluted spectrum d) magnification of the major peak; calculated mass: 15221 g/mol, observed mass: 15221 g/mol.

3.6.2.2 Histone H3 K9Dha

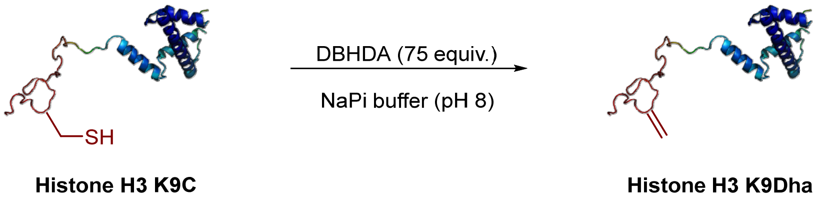

Histone H3 K9C (15.0 mg, 0.99 µmol, 1.00 equiv.) and DTT (37.5 mg, 243 µmol, 250 equiv.) were dissolved in 1000 µL of NaP$_i$ buffer (100 mM, 3 M Gdn·HCl, pH 8.0) by repeated vortexing and sonication. The solution was incubated at room temperature for 1 h before being desalted using GE MiniTrap G-25 desalting columns preconditioned with NaP$_i$ buffer (100 mM, 3 M Gdn·HCl, pH 8.0) according to the manufacturer's specifications. A solution of DBHDA (23.8 mg, 78.9 µmol, 80 equiv.) in 90 µL of DMF was added and the mixture was briefly vortexed and shaken at room temperature for 2 h. The temperature was raised to 37°C and shaking was continued for an additional 2.5 h until completion of the reaction was determined by LCMS. The solution was purified using GE MiniTrap G-25 columns preconditioned with NaP$_i$ buffer (100 mM, 3 M Gdn·HCl, pH 8.0) to yield 4000 µL of a protein solution with a concentration of 2.54 mg/mL as determined by A280 spectrophotometry (10.16 mg, 0.67 µmol, 68%).

The sample was aliquoted, frozen in liquid nitrogen and stored at −80°C.

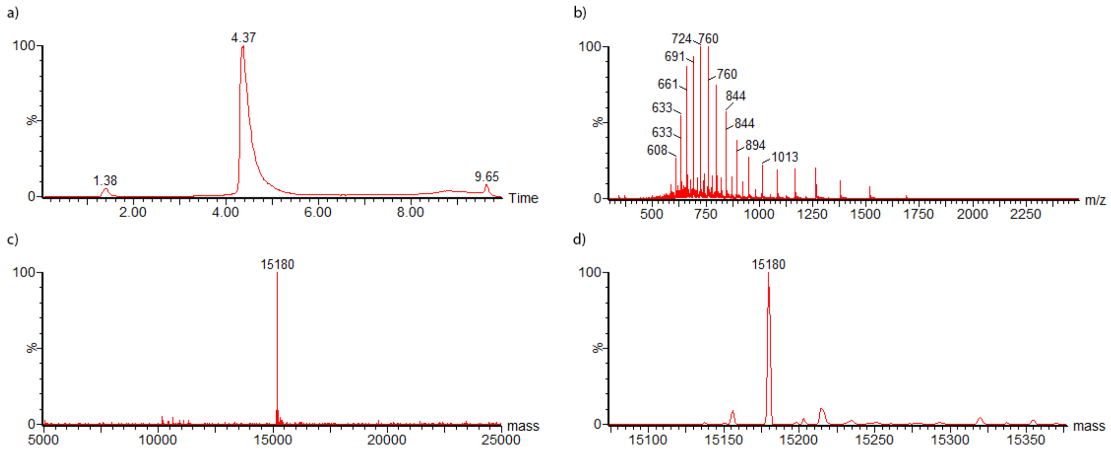

Supplementary Figure S32: LCMS analysis of Histone H3 K9Dha shows expected protein mass. a) total ion chromatogram b) ion series c) deconvoluted spectrum d) magnification of the major peak; calculated mass: 15180 g/mol; observed mass: 15180 g/mol.

3.6.2.3 Histone H4 K16Dha

Histone H4 K16Dha was synthesized following a previously published procedure.*(19)*

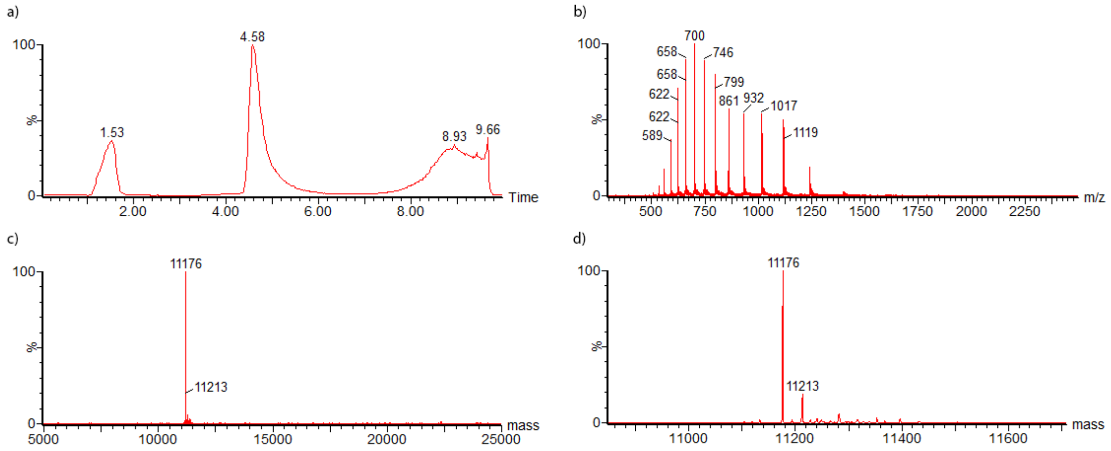

Supplementary Figure S33: LCMS analysis of Histone H4 K16Dha shows expected protein mass. a) total ion chromatogram b) ion series c) deconvoluted spectrum d) magnification of the major peak**;** calculated mass: 11177 g/mol; observed mass: 11176 g/mol.

3.6.2.4 FLAG-Histone H3 K4Dha

FLAG-tagged Histone H3 K4Dha was synthesized following a previously published procedure.*(79)*

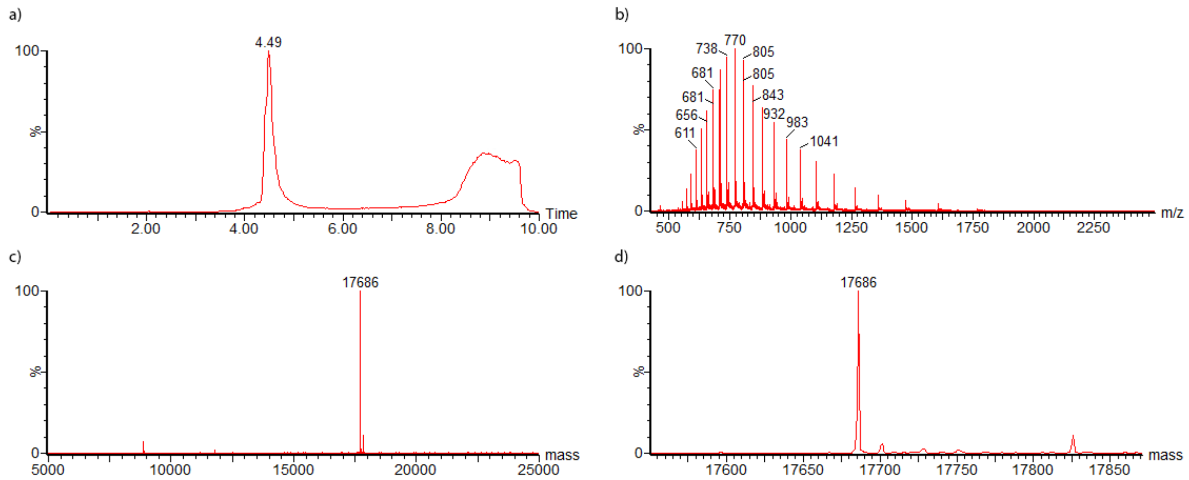

Supplementary Figure S34: LCMS analysis of FLAG-tagged Histone H3 K4Dha shows expected protein mass. a) total ion chromatogram b) ion series c) deconvoluted spectrum d) magnification of the major peak; calculated mass: 17686 g/mol; observed mass: 17686 g/mol.

3.6.2.5 FLAG-Histone H3 K27Dha

FLAG-tagged Histone H3 K27Dha was synthesized following a previously published procedure.*(79)*

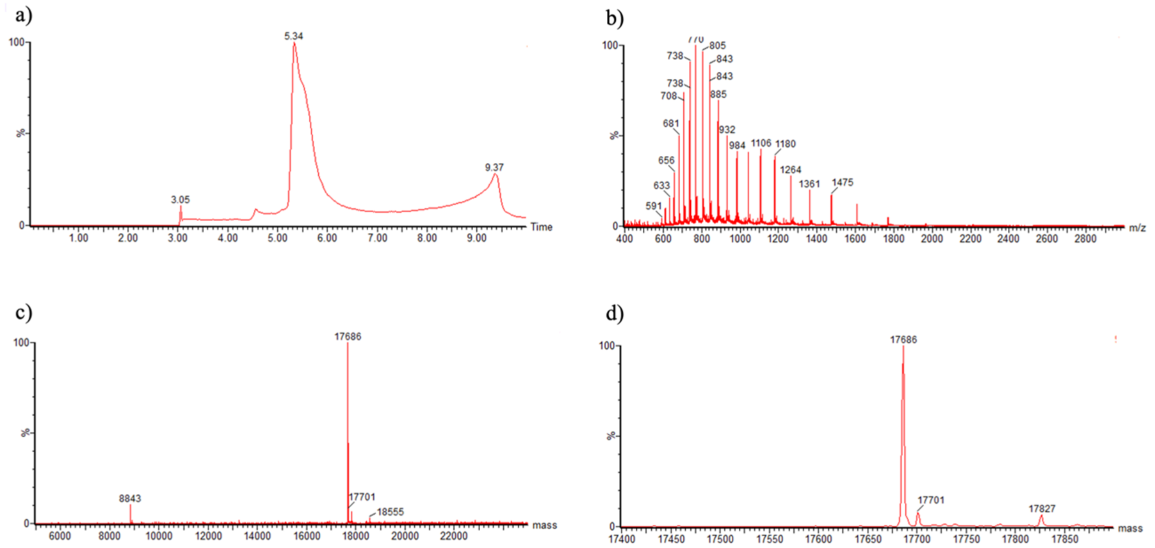

Supplementary Figure S35: LCMS analysis of FLAG-tagged Histone H3 K27Dha shows expected protein mass. a) total ion chromatogram b) ion series c) deconvoluted spectrum d) magnification of the major peak; calculated mass: 17686 g/mol; observed mass: 17686 g/mol.

3.6.2.6 FLAG-Histone H3 K36Dha

FLAG-tagged Histone H3 K36Dha was synthesized following a previously published procedure.*(79)*

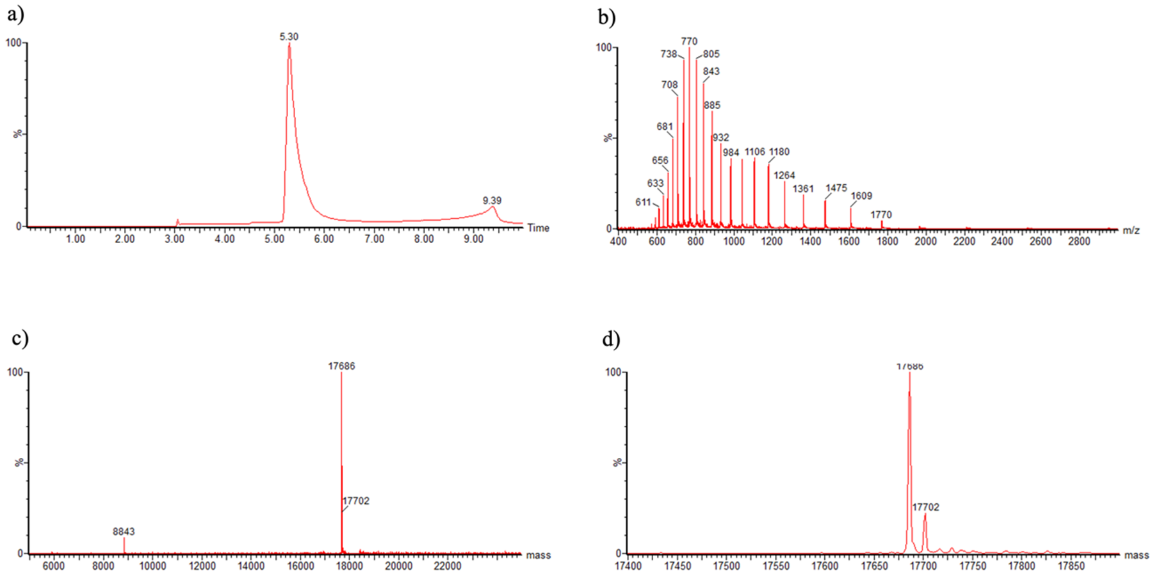

Supplementary Figure S36: LCMS analysis of FLAG-tagged Histone H3 K36Dha shows expected protein mass. a) total ion chromatogram b) ion series c) deconvoluted spectrum d) magnification of the major peak**;** calculated mass: 17686 g/mol; observed mass: 17686 g/mol.

3.6.2.7 Annexin V C316Dha

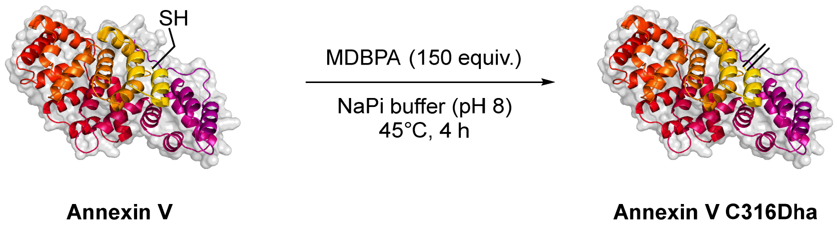

To 450 µL of a solution of Annexin V (2.00 mg/mL, 25.1 nmol, 1.00 equiv.) in NaP_i buffer (50 mM, pH 7.8) was added DTT (0.19 mg, 1.26 µmol, 50 equiv.). The mixture was incubated at room temperature for 20 min. and desalted by passing through a GE MiniTrap G-25 column pre-equilibrated with NaP_i buffer (100 mM, pH 8.0) according to the manufacturer's instructions. A solution of MDBPA (0.69 mg, 2.51 µmol, 100 equiv.) in 45 µL of DMSO was added and the mixture was shaken at 45°C for 3.5 h. Another 50 equiv. of MDBPA in DMSO were added and the mixture was shaken for another 25 min. The mixture was passed through a GE MiniTrap G-25 column pre-equilibrated with NaP_i buffer (100 mM, pH 7.0) and yielding 1.5 mL of a solution of Annexin V C316Dha with a concentration of 0.60 mg/mL (900 µg, 25.1 nmol, quantitative recovery). The product contained 15% double alkylated product.

The sample was aliquoted, frozen in liquid nitrogen and stored at −80°C.

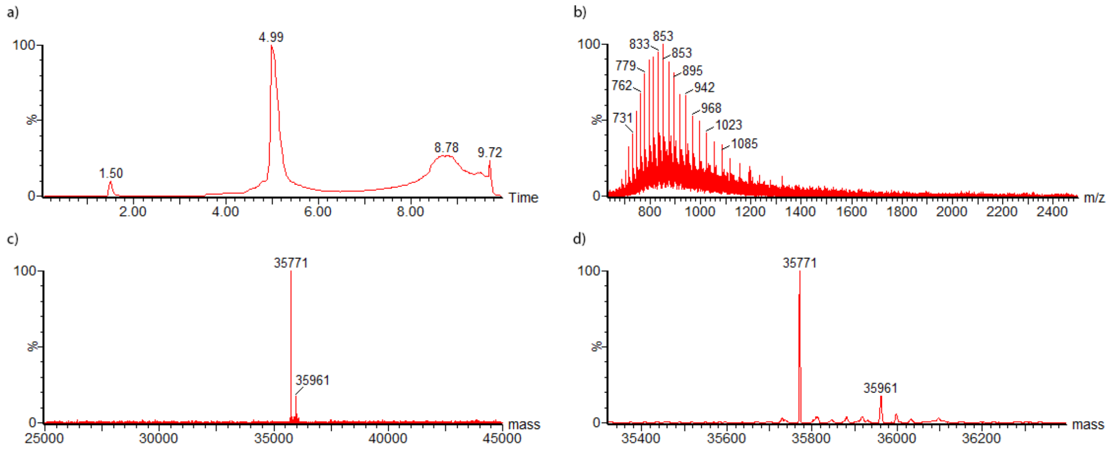

Supplementary Figure S37: LCMS analysis of Annexin V C316Dha shows expected protein mass. a) total ion chromatogram b) ion series c) deconvoluted spectrum d) magnification of the major peak**;** calculated mass: 34772 g/mol; observed mass: 34771 g/mol.

3.6.2.8 Npβ C61Dha

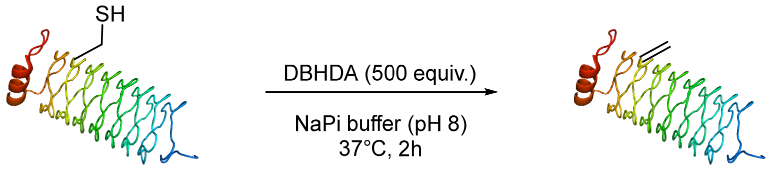

To a solution of Npβ (2.74 mg, 130 nmol, 1.00 equiv.) in 500 µL of NaP_i buffer (50 mM, pH 7.8) was added DTT (1.00 mg, 6.51 µmol, 50 equiv.). The mixture was vortexed and incubated at room temperature for 30 min before it was purified using a GE PD MiniTrap G-25 which was previously equilibrated with NaP_i buffer (100 mM,

pH 8.0) according to the manufacturer's instructions. A solution of DBHDA (19.7 mg, 65.1 µmol, 500 equiv.) in 160 µL of DMF was added to the purified protein and the reaction mixture was shaken at 37°C and 500 rpm for 2 h. The protein was purified using a GE PD MiniTrap G-25 which was previously equilibrated with NaP_i buffer (100 mM, pH 7.0) to yield 2000 µL of protein solution with a concentration of 0.92 mg/mL as determined by A280 spectrophotometry (1.84 mg, 87.6 nmol, 67% recovery).

The sample was aliquoted, frozen in liquid nitrogen and stored at −80°C.

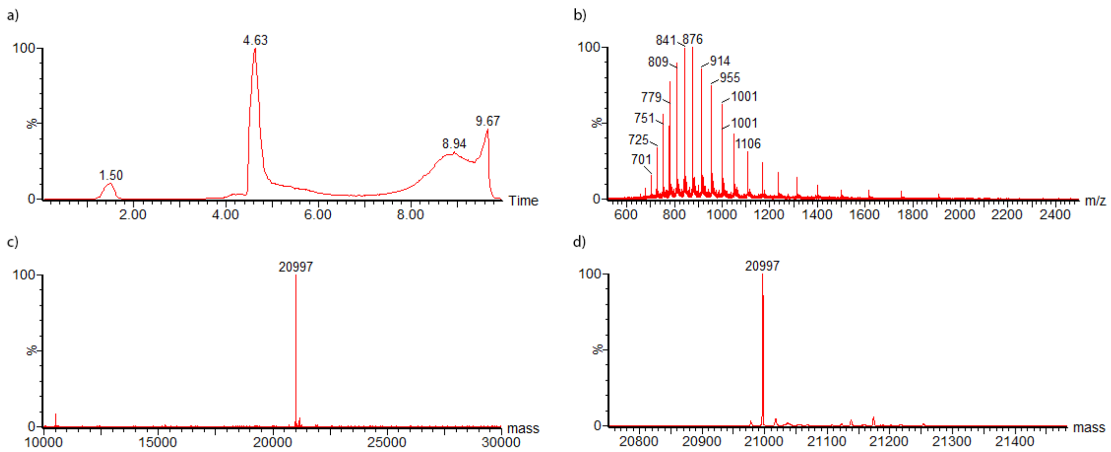

Supplementary Figure S38: LCMS analysis of Npβ C61Dha shows expected protein mass. a) total ion chromatogram b) ion series c) deconvoluted spectrum d) magnification of the major peak; calculated mass: 20997 g/mol; observed mass: 20997 g/mol.

3.6.2.9 pre-SUMO1 C51Dha

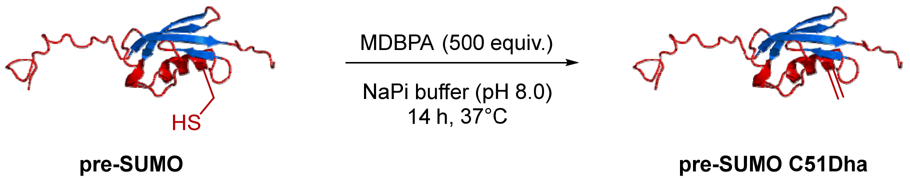

A solution of pre-SUMO1 (4000 µL, 2.30 mg/mL, 737 nmol, 1.00 equiv.) was incubated with DTT (5.7 mg, 36.9 µmol, 50.0 equiv.) for 30 min at room temperature. The protein was purified using GE PD MidiTraps G-25 which were previously equilibrated with NaP_i buffer (100 mM, pH 8.0) according to the manufacturer's instructions. To the purified protein solution was added a solution of MDBPA (57.4 µL, 369 µmol, 500 equiv.) in DMSO (142.6 µL), the mixture was vortexed and shaken at 37°C and 600 rpm for 14 h. The solution was concentrated to 2500 µL using a VivaSpin 20 concentrator (MWCO 3,000 Da). 2000 µL of this solution were purified using GE PD MidiTraps G-25 which were previously equilibrated with NaP_i buffer (100 mM, 3 M Gdn·HCl, pH 7.0) yielding 3000 µL of a solution of pre-SUMO1 C51Dha (2.01 mg, 199 nmol, 27% recovery).

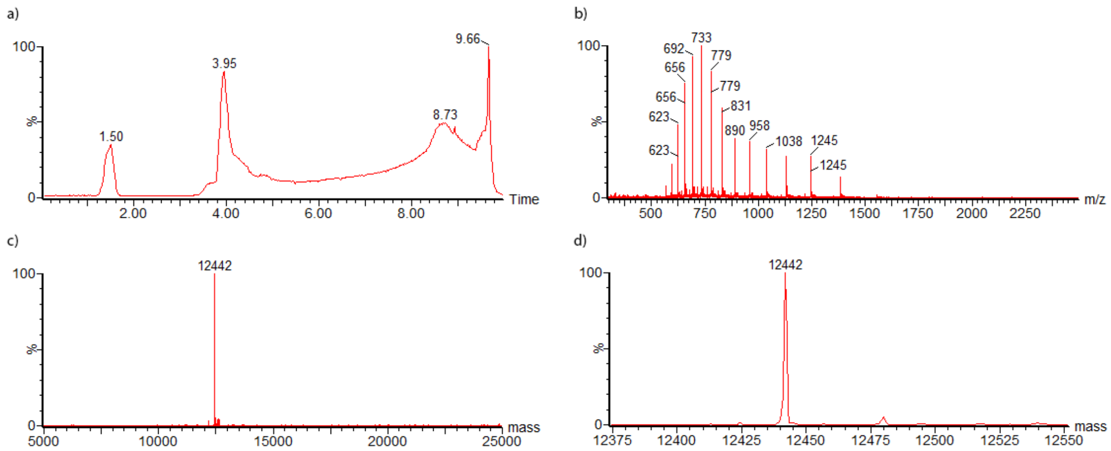

Supplementary Figure S39: LCMS analysis of pre-SUMO1 C51Dha shows expected protein mass. a) total ion chromatogram b) ion series c) deconvoluted spectrum d) magnification of the major peak**;** calculated mass: 12441 g/mol; observed mass: 12442 g/mol.

3.6.2.10 pre-SUMO1 C51Dha H97Dha

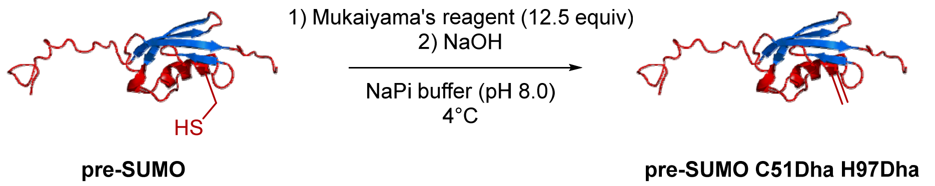

Solid preSUMO1 C51 C97 (1.0 mg, 80.4 nmol, 1.00 equiv) was dissolved in sodium phosphate buffer (500 µL, 50 mM, pH 8.0). The solution was cooled to 4°C before Mukaiyama's reagent (20 µL of a stock solution of 6.4 mg in 500 µL of milli-Q H_2O, 1.00 µmol, 12.5 equiv) was added. The solution was mixed and incubated at 4°C. After 10 min, 60 µL on aqueous NaOH (1.0 M) was added and the mixture was incubated for another 60 min. The reaction was quenched with sodium acetate buffer (500 µL, 200 mM, pH 4.0) before being allowed to warm to room temperature. The reaction mixture was purified via gel filtration using a PD MidiTrap G-25 previously equilibrated with TRIS buffer (100 mM, pH 8.0) to yield 1500 µL of a solution of preSUMO1 Dha51 Dha97 (0.39 mg/mL, 585 µg, 47.0 nmol, 59% recovery).

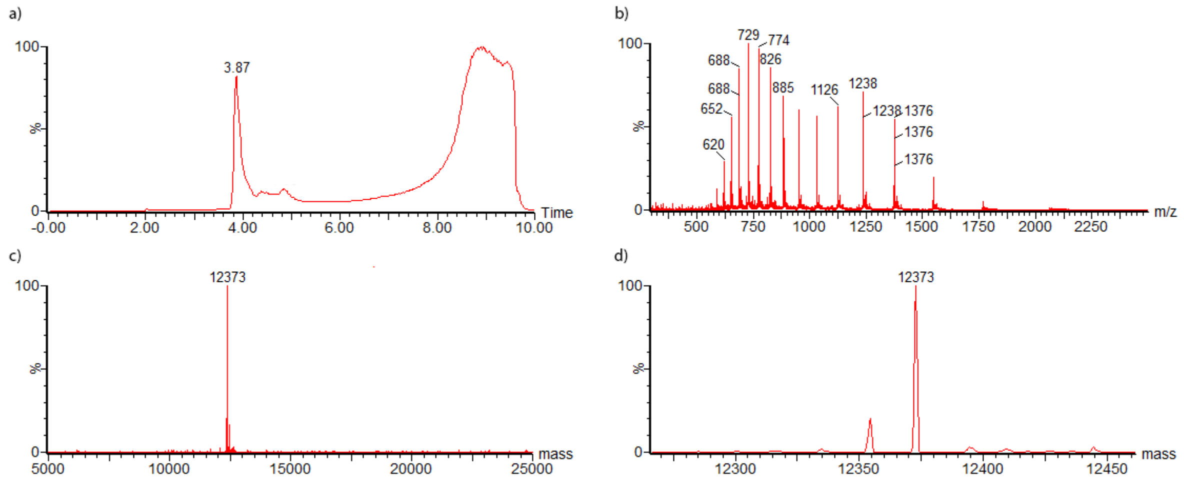

Supplementary Figure S40: LCMS analysis of pre-SUMO1 C51Dha shows expected protein mass. a) total ion chromatogram b) ion series c) deconvoluted spectrum d) magnification of the major peak**;** calculated mass: 12373 g/mol; observed mass: 12373 g/mol.

3.6.2.11 cAbVCAM Q1N Q118Dha

An aliquot of cAbVCAM (1.0 mL, 2.0 mg/mlL 137 µM) in sodium phosphate buffer (50 mM, pH 8.0) was treated with 10 equiv. of DTT (0.21 mg, 0.14 mmol) and the reaction was incubated at 25°C for 30 minutes. Then, a stock solution of 2,5-dibromohexanediamide (DBHDA; 0.5 M in DMSO) was added (67 µL , 250 equiv.) and the reaction mixture was shaken at 37°C and 500 rpm for 2 hours. The reaction was desalted with a MidiTrap G-25, preequilibrated in sodium phosphate buffer (50 mM, pH 8.0). The reaction was further incubated at 41°C for three more hours, when completion was determined by LC-MS. The sample was aliquoted, frozen in liquid nitrogen and stored at −80°C.

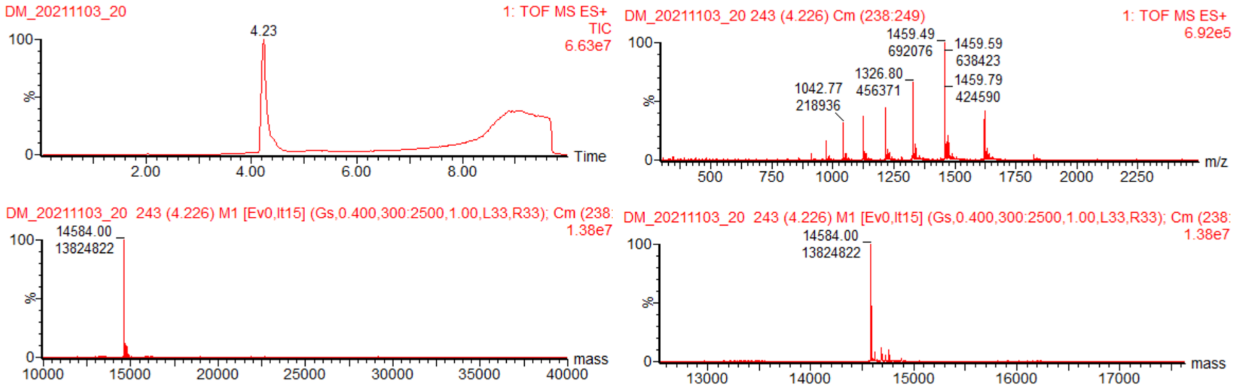

Supplementary Figure S41: LCMS analysis of cAbVCAM Q1N Q118Dha shows expected protein mass. a) total ion chromatogram b) ion series c) deconvoluted spectrum d) magnification of the major peak**;** calculated mass: 14585 g/mol; observed mass: 14584 g/mol.

3.6.3 Protein Cleavage

3.6.3.1 FLAG-Histone H3 Dha4

FLAG-Histone H3 Dha4 was desalted into milli-Q water using a PD SpinTrap G-25 microspin column (Cytiva). Stock solutions of tetrahydroxydiboron (5.6 mg in 1000 µL) and sodium ascorbate (14.0 mg in 500 µL) in milli-Q water were prepared freshly directly before the reactions were conducted.

To 100 µL of a solution of FLAG-Histone H3 Dha4 in milli-Q water (100 µg, 5.64 nmol, 1.00 equiv) were added aliquots of the previously prepared stock solutions of sodium ascorbate (2 µL, 282 nmol, 50 equiv) and tetrahydroxydiboron (18 µL, 1.13 µmol, 200 equiv). The solution was briefly vortexed and incubated at room temperature for 21 hours before being analysed by LC-MS (>95% conversion).

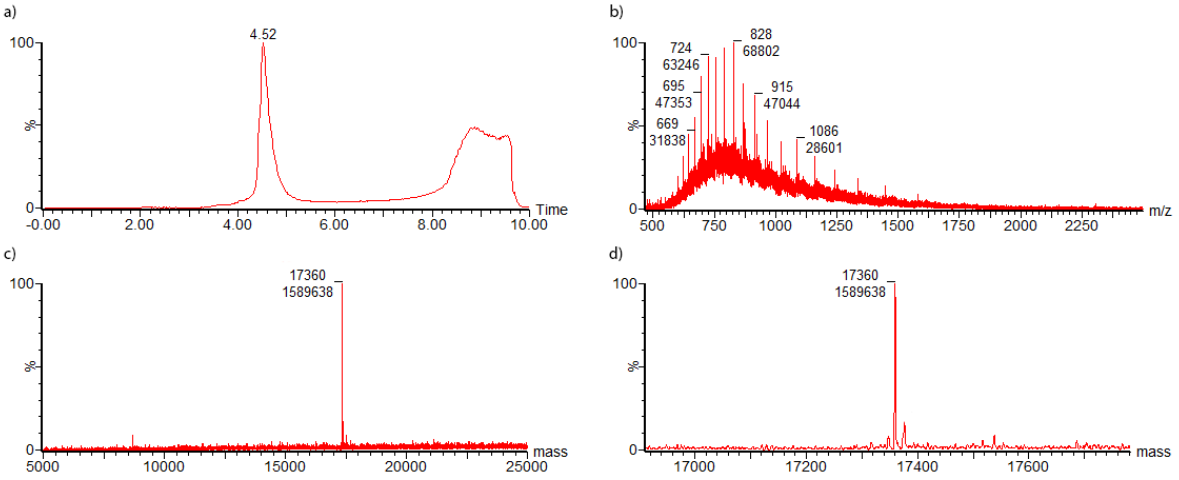

Supplementary Figure S42: LCMS analysis of the cleavage of FLAG-tagged Histone H3 Dha4 shows expected protein mass. a) total ion chromatogram b) ion series of the *C*-terminal fragment c) deconvoluted spectrum of the *C*-terminal fragment d) magnification of the major peak**;** calculated mass: 17361 g/mol; observed mass: 17360 g/mol.

3.6.3.2 Histone H3 Dha9

Lyophilized Histone H3 Dha9 (500 µg) was dissolved in milli-Q water (500 µL). Stock solutions of tetrahydroxydiboron (5.9 mg in 1000 µL) and sodium ascorbate (16.3 mg in 500 µL) in milli-Q water were prepared freshly directly before the reactions were conducted.

To the solution of Histone H3 Dha9 (500 µL, 500 µg, 32.9 nmol, 1.00 equiv) were added aliquots of the previously prepared stock solutions of sodium ascorbate (10 µL, 1.64 µmol, 50 equiv) and tetrahydroxydiboron (100 µL, 6.59 µmol, 200 equiv). The solution was briefly vortexed and incubated at room temperature for 23 hours before being analysed by LC-MS (>95% conversion).

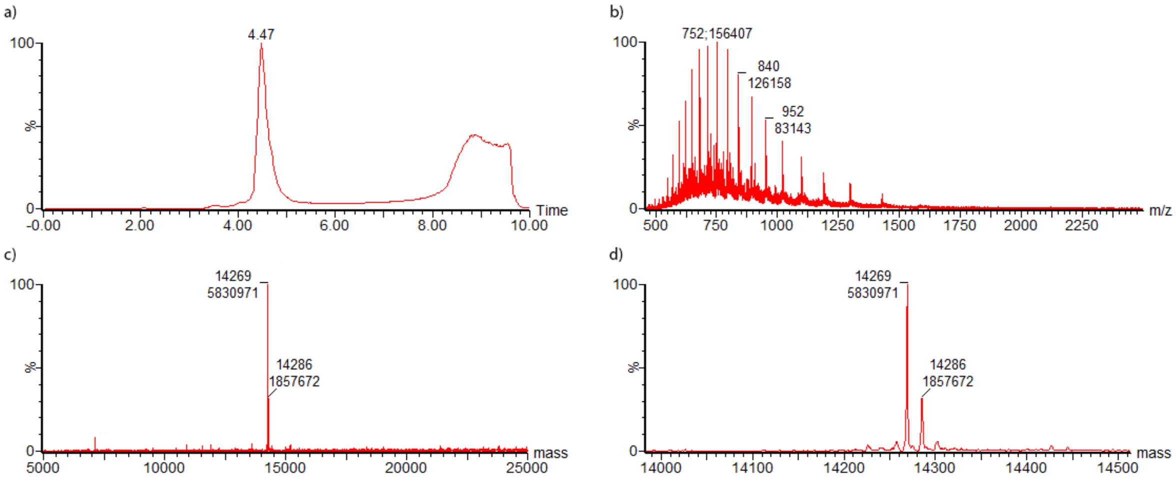

Supplementary Figure S43: LCMS analysis of the cleavage of Histone H3 Dha9 shows expected protein mass. a) total ion chromatogram b) ion series of the *C*-terminal fragment c) deconvoluted spectrum of the *C*-terminal fragment d) magnification of the major peak**;** calculated mass: 14270 g/mol; observed mass: 14269 g/mol, 14286 g/mol (*C*-terminal fragment + oxidation).

3.6.3.3 Histone H3 Dha10

Lyophilized Histone H3 Dha10 was dissolved in milli-Q water at 1.0 mg/mL. Stock solutions of tetrahydroxydiboron (5.9 mg in 1000 µL) and sodium ascorbate (13.0 mg in 1000 µL) in milli-Q water were prepared.

40 µL of Histone H3 Dha10 (40 µg, 2.63 nmol) were treated with sodium ascorbate (2 µL, 131 nmol, 50 equiv) followed by tetrahydroxydiboron (8 µL, 526 nmol, 200 equiv) and incubated at room temperature for 24 hours before being analysed by LC-MS (>95% conversion).

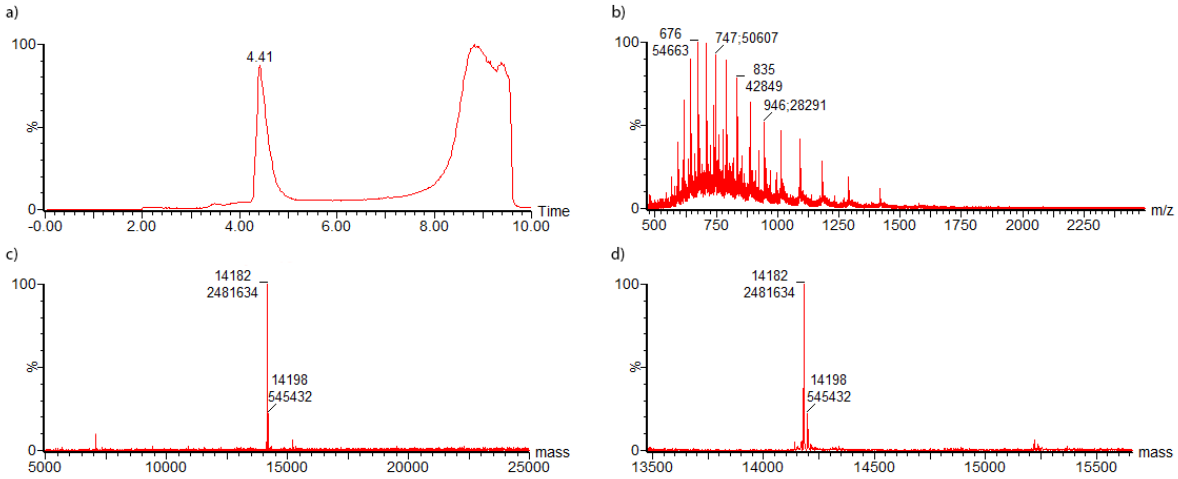

Supplementary Figure S44: LCMS analysis of the cleavage of Histone H3 Dha10 shows expected protein mass. a) total ion chromatogram b) ion series of the *C*-terminal fragment c) deconvoluted spectrum of the *C*-terminal fragment d) magnification of the major peak**;** calculated mass: 14182 g/mol; observed mass: 14182 g/mol, 14199 g/mol (*C*-terminal fragment + oxidation).

3.6.3.4 FLAG-Histone H3 Dha27

FLAG-Histone H3 Dha27 was desalted into milli-Q water using a PD G-25 minitrap column (Cytiva). Stock solutions of tetrahydroxydiboron (8.9 mg in 1000 µL, 100 mM) and sodium ascorbate (9.9 mg in 500 µL, 50 mM) in milli-Q water were prepared freshly directly before the reactions were conducted.

To 50 µL of a solution of FLAG-Histone H3 Dha27 in milli-Q water (46 µg, 2.60 nmol, 1.00 equiv, 51 µM) were added aliquots of the previously prepared stock solutions of sodium ascorbate (2.94 µL, 151 nmol, 58 equiv, 2.9 mM) and tetrahydroxydiboron (5.76 µL, 0.59 µmol, 226 equiv, 11.6 mM). The solution was briefly vortexed and incubated at room temperature for 18 hours before being analysed by LC-MS (>95% conversion).

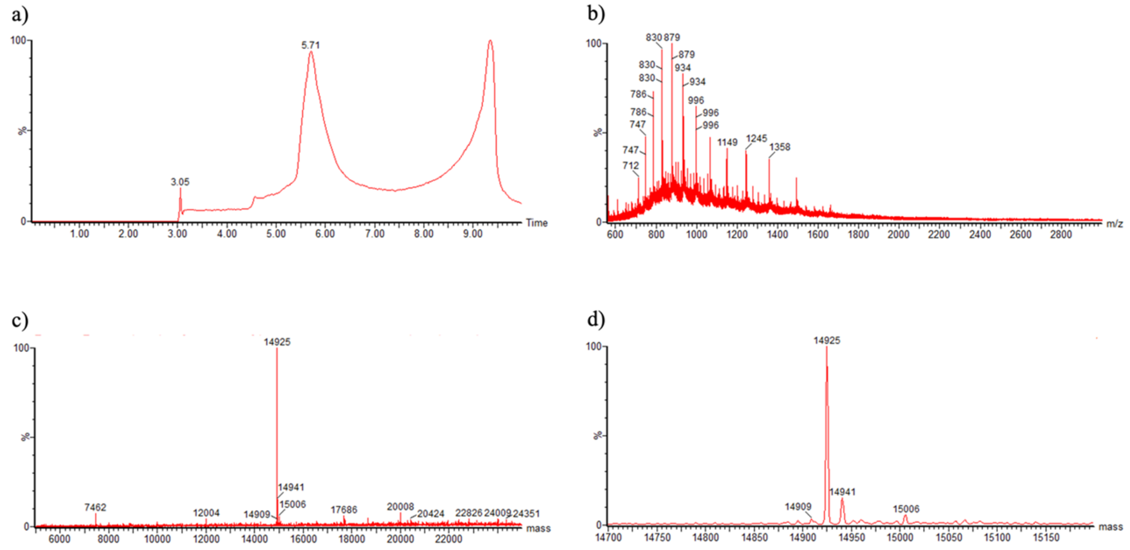

Supplementary Figure S45: LCMS analysis of the cleavage of FLAG-tagged Histone H3 K27Dha shows expected protein mass. a) total ion chromatogram b) ion series of the *C*-terminal fragment c) deconvoluted spectrum of the *C*-terminal fragment d) magnification of the major peak; calculated mass: 14925 g/mol; observed mass: 14925 g/mol.

3.6.3.5 FLAG-Histone H3 Dha36

FLAG-Histone H3 Dha36 was desalted into milli-Q water using a PD G-25 minitrap column (Cytiva). Stock solutions of tetrahydroxydiboron (8.9 mg in 1000 µL, 100 mM) and sodium ascorbate (9.9 mg in 500 µL, 50 mM) in milli-Q water were prepared freshly directly before the reactions were conducted.

To 50 µL of a solution of FLAG-Histone H3 Dha36 in milli-Q water (46 µg, 2.60 nmol, 1.00 equiv, 51 µM) were added aliquots of the previously prepared stock solutions of sodium ascorbate (2.94 µL, 151 nmol, 58 equiv, 2.9 mM) and tetrahydroxydiboron (5.76 µL, 0.59 µmol, 226 equiv, 11.6 mM). The solution was briefly vortexed and incubated at room temperature for 18 hours before being analysed by LC-MS (>95% conversion).

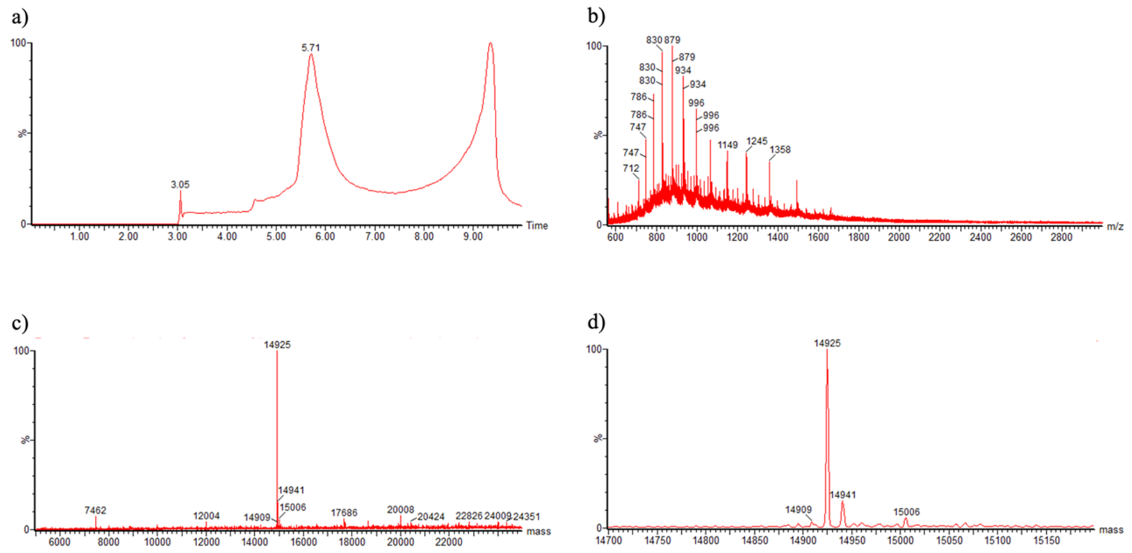

Supplementary Figure S46: LCMS analysis of the cleavage of FLAG-tagged Histone H3 K36Dha shows expected protein mass. a) total ion chromatogram b) ion series of the *C*-terminal fragment c) deconvoluted spectrum of the *C*-terminal fragment d) magnification of the major peak; calculated mass: 14925 g/mol; observed mass: 14925 g/mol.

3.6.3.6 Histone H4 Dha16

Histone H4 Dha16 was buffer exchanged into borate buffer (100 mM, pH 8.0) using a PD SpinTrap G-25 microspin column (Cytiva) and following the manufacturer's instructions. Stock solutions of tetrahydroxydiboron (5.8 mg in 1000 µL) and sodium ascorbate (13.3 mg in 500 µL) in milli-Q water were prepared freshly directly before the reactions were conducted.

To 90 µL of a solution of Histone H4 Dha16 in borate buffer (90 µg, 8.05 nmol, 1.00 equiv) were added aliquots of the previously prepared stock solutions of sodium ascorbate (3 µL, 403 nmol, 50 equiv) and tetrahydroxydiboron (25 µL, 1.61 µmol, 200 equiv). The solution was briefly vortexed and incubated at room temperature for 17 hours before being analysed by LC-MS (>95% conversion).

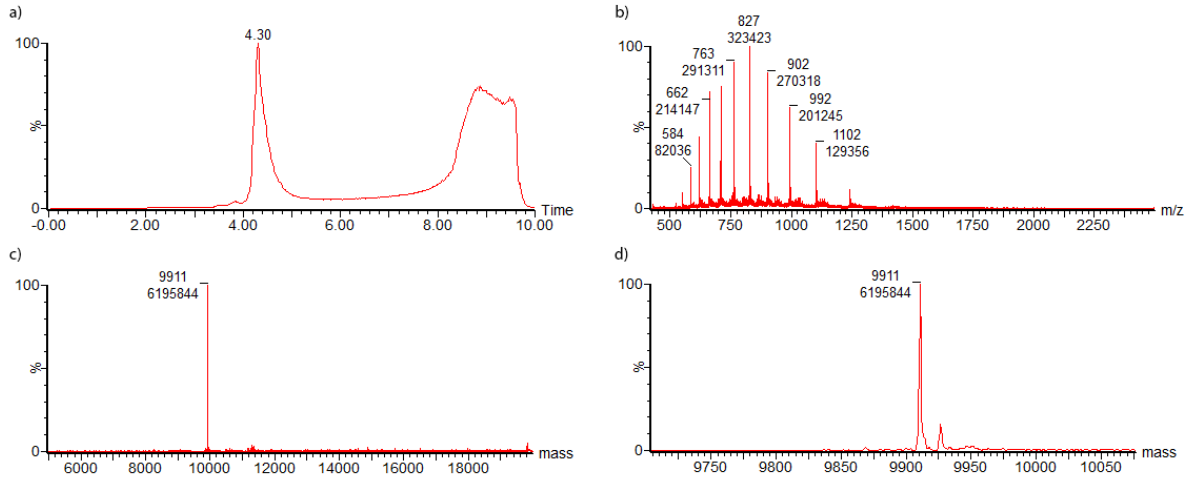

Supplementary Figure S47: LCMS analysis of the cleavage of Histone H4 Dha16 shows expected protein mass. a) total ion chromatogram b) ion series of the *C*-terminal fragment c) deconvoluted spectrum of the *C*-terminal fragment d) magnification of the major peak; calculated mass: 9912 g/mol; observed mass: 9911 g/mol.

3.6.3.7 Npβ Dha61

Npβ Dha61 was buffer exchanged into sodium borate buffer (100 mM, pH 8.0) using a PD SpinTrap G-25 microspin column (Cytiva) and following the manufacturer's instructions. The protein concentration was determined by A280 spectrophotometry (0.90 mg/mL).

Reaction at room temperature

Stock solutions of tetrahydroxydiboron (5.5 mg in 1000 µL) and sodium ascorbate (15.3 mg in 1000 µL) in milli-Q water were prepared freshly directly before the reactions were conducted. To 60 µL of a solution of Npβ Dha61 (0.90 mg/mL, 54.0 µg, 2.57 nmol) were added aliquots of the previously prepared stock solutions of sodium ascorbate (2.5 µL, 193 nmol, 75 equiv) and tetrahydroxydiboron (12.5 µL, 771 nmol, 300 equiv). The solution was briefly vortexed and incubated at room temperature for 24 h before being analysed by LC-MS (21% conversion).

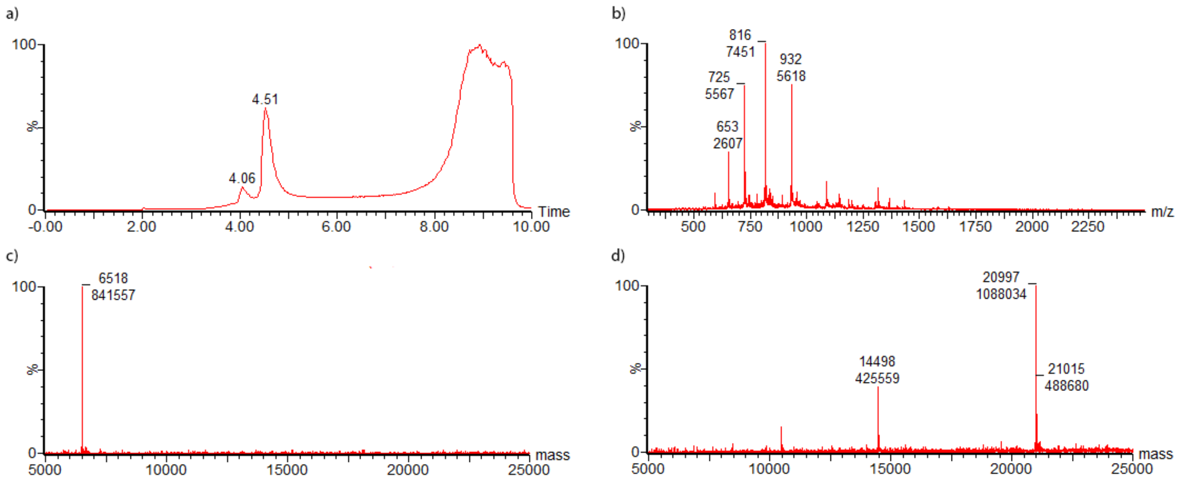

Supplementary Figure S48: LCMS analysis of the cleavage of Npβ Dha61 at room temperature shows expected protein mass. a) total ion chromatogram b) ion series of the *N*-terminal fragment c) deconvoluted spectrum of the *N*-terminal fragment d) deconvoluted spectrum of the *C*-terminal fragment; calculated mass for the *N*-terminal fragment: 6519 g/mol; observed mass: 6518 g/mol; calculated mass for the *C*-terminal fragment: 14499 g/mol; observed mass: 14498 g/mol.

Reaction at 45°C

Stock solutions of tetrahydroxydiboron (6.0 mg in 1000 µL) and sodium ascorbate (9.9 mg in 500 µL) in milli-Q water were prepared freshly directly before the reactions were conducted. To 65 µL of a solution of Npβ Dha61 (0.90 mg/mL, 58.5 µg, 2.79 nmol) were added aliquots of the previously prepared stock solutions of sodium ascorbate (5 µL, 5 mM final concentration) and tetrahydroxydiboron (30 µL, 20 mM final concentration). The mixture was briefly vortexed and shaken at 45°C and 500 rpm for 24 hours before being analysed by LC-MS (85% conversion).

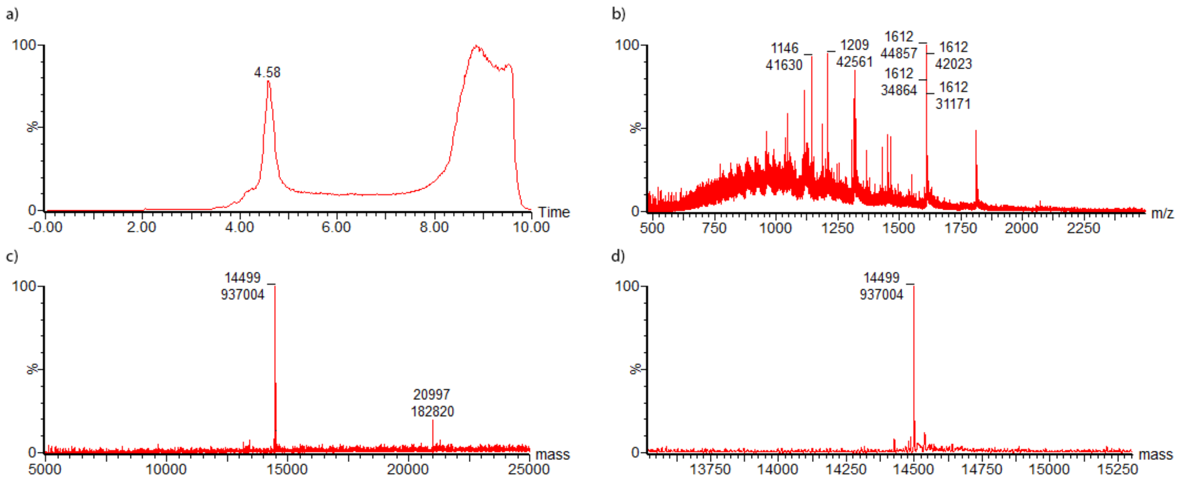

Supplementary Figure S49: LCMS analysis of the cleavage of Npβ Dha61 at 45°C shows expected protein mass. a) total ion chromatogram b) ion series of the *C*-terminal fragment c) deconvoluted spectrum of the *C*-terminal fragment d) magnification of the major peak**;** calculated mass: 14499 g/mol; observed mass: 14499 g/mol.

Reaction analysis by SDS-PAGE, Coomassie staining and densitometry

20 µL of sample were mixed with 5 µL of Laemmli buffer (5×) and heated at 95°C for 5 minutes. 10 µL of the sample analysed by gel electrophoresis (Invitrogen NuPAGE 10% bis-TRIS gel, MES buffer, 200 V, 75 min, 4°C). Protein bands were visualized by Coomassie staining. Stained gels were analysed by densitometric analysis in ImageJ 1.53f.*(65)* Conversions were calculated based on the area of cleavage products compared to the area of intact protein.

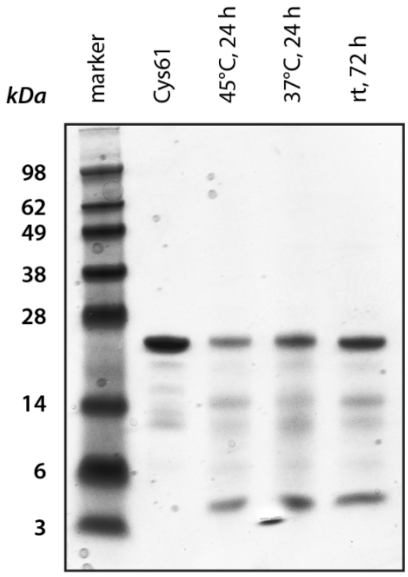

Supplementary Figure S50: SDS-PAGE analysis of diboron-promoted cleavage of Npβ Dha61 under various conditions was used for densitometric determination of cleavage conversions.

Supplementary Table S3: Densitometric determination of cleavage conversions for diboron-promoted cleavage of Npβ Dha61 under various conditions using SDS-PAGE and Coomassie staining.

Entry	Conditions	Cleavage [%]
**1**	45°C, 24 h, borate buffer (100 mM, pH 8.0)	52%
**2**	37°C, 24 h, borate buffer (100 mM, pH 8.0)	45%
**3**	room temperature, 3 days, borate buffer (100 mM, pH 8.0)	46%

3.6.3.8 preSUMO1 Dha51

preSUMO1 Dha51 was buffer exchanged into borate buffer (100 mM, pH 8.0) or TRIS buffer (100 mM, pH 8.0) using PD SpinTrap G-25 microspin columns (Cytiva) and following the manufacturer's instructions. The protein concentration was determined by A280 spectrophotometry (borate buffer: 0.41 mg/mL, TRIS buffer: 0.30 mg/mL).

Stock solutions of tetrahydroxydiboron (5.4 mg in 1000 µL) and sodium ascorbate (14.9 mg in 1000 µL) in milli-Q water were prepared freshly directly before the reactions were conducted. To 30 µL of a solution of preSUMO1 Dha51 in borate or TRIS buffer (9 µg – 12.3 µg, 0.72 nmol – 0.99 nmol) were added aliquots of the previously prepared stock solutions of sodium ascorbate (1.25 µL, final concentration 2.5 mM) and tetrahydroxydiboron (6.25 µL, final concentration 10 mM). The solution was briefly vortexed and incubated at room temperature for 48 hours or shaken at 37° and 500 rpm for 24 h before being analysed by SDS-PAGE (up to 14% conversion).

20 µL of sample were mixed with 5 µL of Laemmli buffer (5×) and heated at 95°C for 5 minutes. 10 µL of the sample analysed by gel electrophoresis (Invitrogen NuPAGE 10% bis-TRIS gel, MES buffer, 200 V, 120 min, 4°C). Protein bands were visualized by Coomassie staining. Stained gels were analysed by densitometric analysis in ImageJ 1.53f.*(65)* Conversions were calculated based on the area of cleavage products compared to the area of intact protein.

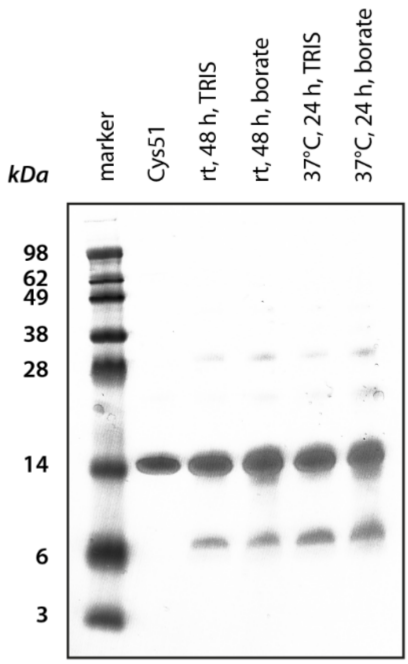

Supplementary Figure S51: SDS-PAGE analysis of diboron-promoted cleavage of preSUMO1 Dha51 under various conditions was used for densitometric determination of cleavage conversions.

Supplementary Table S4: Densitometric determination of cleavage conversions for diboron-promoted cleavage of Npβ Dha61 under various conditions using SDS-PAGE and Coomassie staining.

Entry	Conditions	Cleavage [%]
**1**	room temperature, 2 days, TRIS buffer (100 mM, pH 8.0)	14%
**2**	room temperature, 2 days, borate buffer (100 mM, pH 8.0)	12%
**3**	37°C, 24 h, TRIS buffer (100 mM, pH 8.0)	11%
**4**	37°C, 24 h, borate buffer (100 mM, pH 8.0)	11%

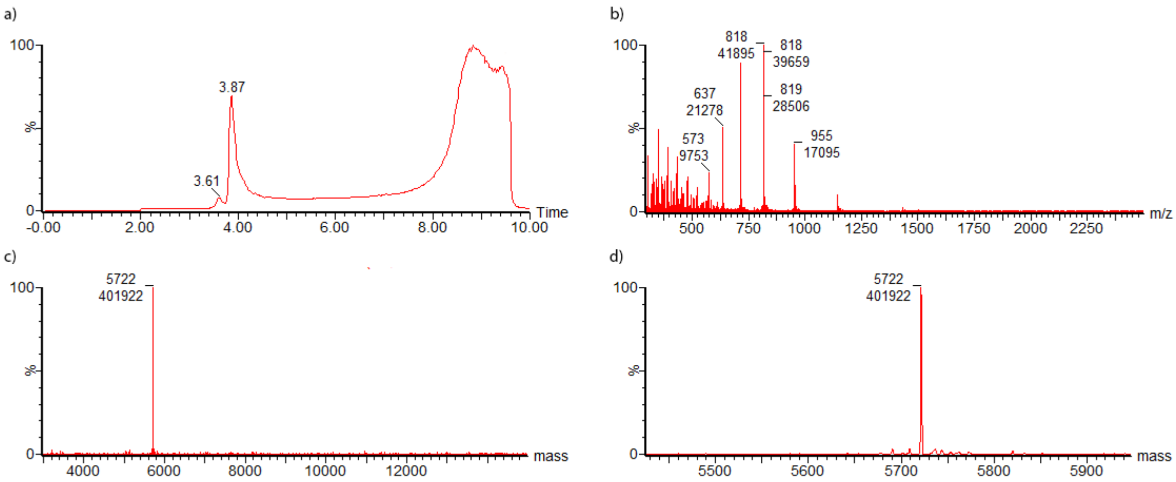

Supplementary Figure S52: LCMS analysis of the cleavage of preSUMO1 Dha51 shows expected protein mass. a) total ion chromatogram b) ion series of the *N*-terminal fragment c) deconvoluted spectrum of the *N*-terminal fragment d) magnification of the major peak**;** calculated mass: 5722 g/mol; observed mass: 5722 g/mol.

3.6.3.9 Annexin V Dha316

Annexin V Dha316 was buffer exchanged into borate buffer (100 mM, pH 8.0) using a PD SpinTrap G-25 microspin column (Cytiva) and following the manufacturer's instructions. The protein concentration was determined by A280 spectrophotometry (0.11 mg/mL).

Stock solutions of tetrahydroxydiboron (6.0 mg in 1000 µL) and sodium ascorbate (9.9 mg in 500 µL) in milli-Q water were prepared freshly directly before the reactions were conducted. To 65 µL of a solution of Annexin V Dha316 (0.11 mg/mL, 7.15 µg, 0.20 nmol) were added aliquots of the previously prepared stock solutions of sodium ascorbate (5 µL, 5 mM final concentration) and tetrahydroxydiboron (30 µL, 20 mM final concentration). The mixture was briefly vortexed and shaken at 45°C and 500 rpm for up to 24 hours before being analysed by LC-MS (39% after 3 hours, 52% after 24 hours).

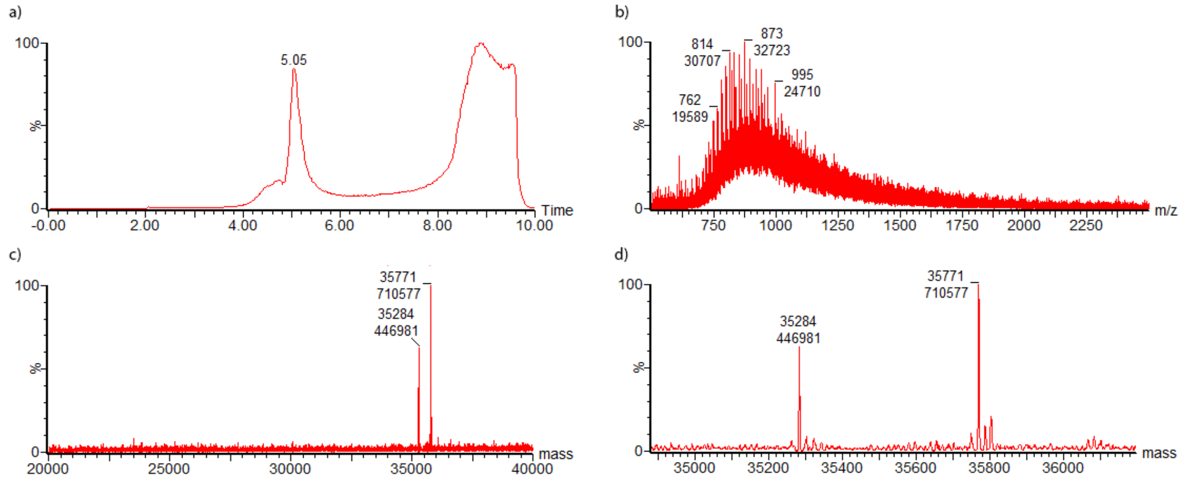

Supplementary Figure S53: LCMS analysis of the cleavage of Annexin V Dha316 **shows expected protein mass.** a) total ion chromatogram b) ion series c) deconvoluted spectrum d) magnification of the major peak; calculated mass: 35285 g/mol; observed mass: 35284 g/mol.

3.6.3.10 preSUMO1 Dha51 Dha97

Stock solutions of tetrahydroxydiboron (5.6 mg in 1000 µL) and sodium ascorbate (6.2 mg in 500 µL) in milli-Q water were prepared freshly directly before the reactions were conducted. To 60 µL of a solution of preSUMO1 Dha51 Dha97 in TRIS buffer (100 mM, pH 8.0) (0.39 mg/mL, 23.4 µg, 1.88 nmol, 1.00 equiv) were added aliquots of the previously prepared stock solutions of sodium ascorbate (3.0 µL, 188 nmol, 100 equiv) and tetrahydroxydiboron (12 µL, 752 nmol, 400 equiv). The mixture was vortexed and incubated at room temperature for 18 h.

LCMS analysis

5 µL of the reaction mixture were quenched by addition of 2 µL of 10% formic acid in milli-Q water (v/v). The sample was diluted with 50 µL of 0.1% formic acid in milli-Q water (v/v) and analysed by LCMS. The deconvoluted LCMS spectrum showed >90% cleavage at site 98. The total ion chromatogram showed <10% cleavage at site 51.

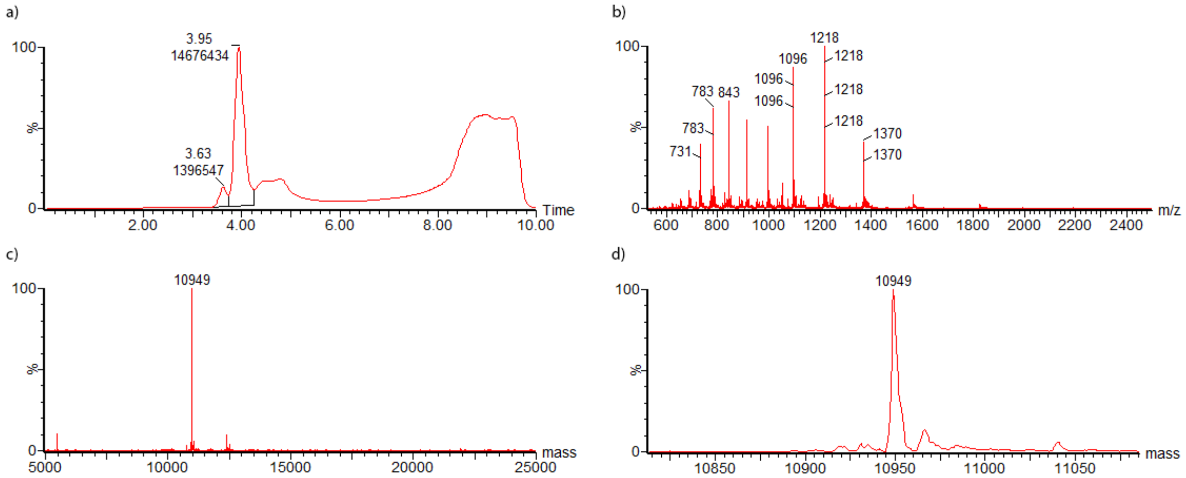

Supplementary Figure S54: LCMS analysis of the cleavage of preSUMO1 Dha51 Dha97 shows expected protein mass. a) total ion chromatogram b) ion series c) deconvoluted spectrum d) magnification of the major peak**;** calculated mass: 10950 g/mol; observed mass: 10949 g/mol.

SDS-PAGE analysis

10 µL of sample were mixed with 2.5 µL of Laemmli buffer (5×) and heated at 95°C for 5 minutes. 10 µL of the sample analysed by gel electrophoresis (Invitrogen NuPAGE 10% bis-TRIS gel, MES buffer, 200 V, 50 min, room temperature). Protein bands were visualized by Coomassie staining. Stained gels were analysed by densitometric analysis in ImageJ 1.53f.*(65)* Cleavage at site 51 was calculated based on the area of cleavage products compared to the area of intact protein. 78% of protein was not cleaved at site 51.

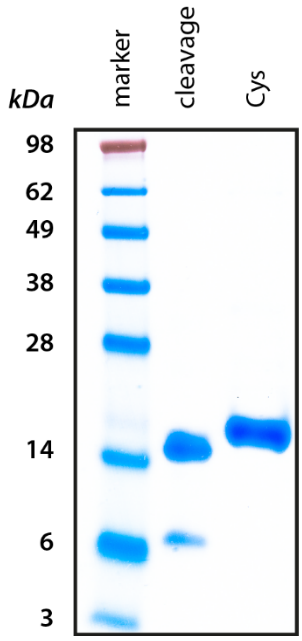

Supplementary Figure S55: Analysis of cleavage of preSUMO1 Dha51 Dha97 via SDS-PAGE shows high selectivity for cleavage at Dha97 over Dha51. Conditions: MES buffer, 200 V, 50 min, 10% Bis-TRIS gel, room temperature and coomassie staining (marker = SeeBlue® Plus2 Protein Standard, cleavage = reaction mixture, Cys = preSUMO1 C51 C97).

Subsequent functionalization: Thia-Michael addition

50 µL of the reaction mixture were treated with 0.5 µL of BME (1%, v/v). The mixture was shaken (500 rpm) at 37°C for 3 h before being analysed by LCMS. 81% conversion to the thia-Michael adduct was observed.

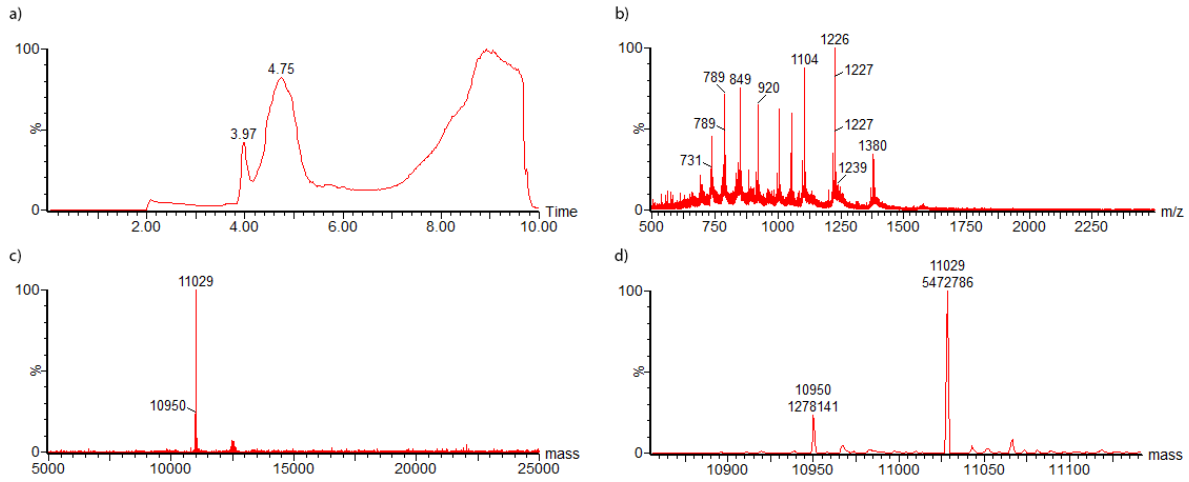

Supplementary Figure S56: LCMS analysis of thia-Michael addition of BME to cleaved preSUMO1 Dha51 Dha97 shows expected protein mass. a) total ion chromatogram b) ion series c) deconvoluted spectrum d) magnification of the major peak**;** calculated mass: 11028 g/mol; observed mass: 11029 g/mol.

3.6.3.11 cAbVCAM Q1N Q118Dha

cAbVCAM Q1N Q118Dha was concentrated to 4.5 mg/mL using a VivaSpin 500 concentrator (Sartorius). A solution of cAbVCAM Q1N Q118Dha (144 µg, 9.87 nmol, 1.00 equiv, 200 µM) in NaP_i (50 mM, pH 8) was prepared and added to solid sodium ascorbate (0.25 mg, 1.23 µmol, 125 equiv, 25 mM final concentration) and tetrahydroxydiboron (0.45 mg, 4.94 µmol, 500 equiv, 100 mM final concentration). The solution was briefly vortexed and incubated at room temperature for 18 hours before being analysed by LC-MS (55% conversion).

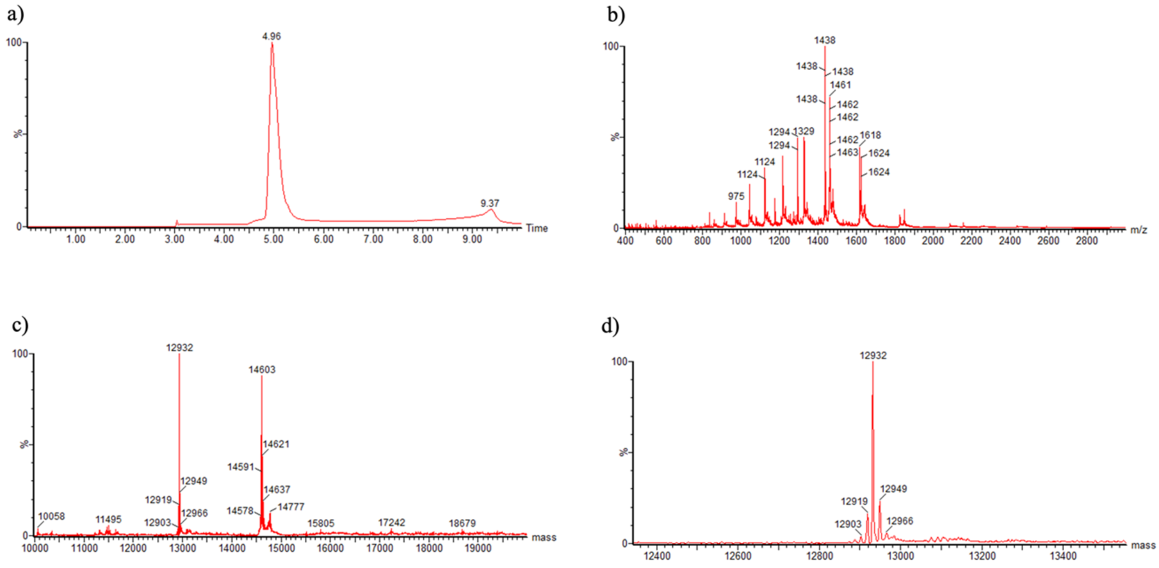

Supplementary Figure S57: LCMS analysis of the cleavage of cAbVCAM Q1N Q118Dha shows expected protein mass. a) total ion chromatogram b) ion series of the N-terminal fragment c) deconvoluted spectrum of the N-terminal fragment d) magnification of the major peak; calculated mass: 12932 g/mol; observed mass: 12932 g/mol.

Reaction Analysis by Ellman's Test

To demostarte that the disulfide bridge remained intact – the protein was reacted with Ellman's reagent (5,5′-dithiobis-(2-nitrobenzoic acid), DTNB). Three samples were tested – Cys, Dha and the cleavage product.

Ellman's reagent (4 mg) was dissolved into 1 mL buffer (NaP_i, 50 mM, pH 8). Samples were made up as such: 31.25 µL buffer, 1.25 µL reagent and 6.25 µL of sample. These were then incubated for 15 min before being diluted into 1% formic acid in water (v/v) for LCMS analysis.

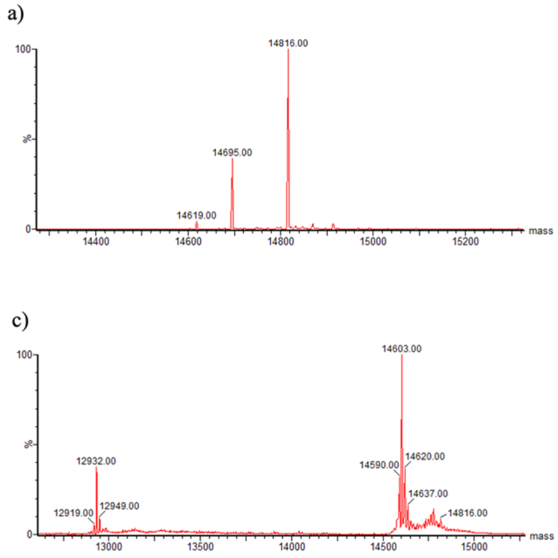

Supplementary Figure S58: LCMS analysis of Ellman's test with cAbVCAM Q1N Q118Dha confirms the absence of disulfide reduction. a) cAbVCAM Q1N Q118C - calculated mass: 14817 g/mol; observed mass: 14816 g/mol b) cAbVCAM Q1N Q118Dha (no reaction) c) cAbVCAM Q1N Q118Dha cleavage product (no reaction).

3.7 Isotopic Pattern Analysis

Isotopic pattern analysis was conducted for the *N*-terminal fragments observed for the cleavage of Npβ Dha61 and preSUMO1 Dha51 in order to distinguish between an unmodified and amidated *C*-terminus. In both cases, three different charge states (Npβ: 7+, 8+, 9+; preSUMO1: 6+, 7+, 8+) were analysed. Isotope patterns were calculated for the *N*-terminal cleavage fragments containing either unmodified and amidated *C*-termini using the isotope modelling algorithm included in MassLynx 4.1 (Waters). Calculated isotope patterns were aligned with the observed isotope patterns by their relative intensities and the deviation of the calculated masses for the unmodified (OH) and amidated (NH_2) *C*-termini from the observed spectrum was calculated. An average deviation was calculated for each of the analysed charge states.

Supplementary Table S5: Isotopic pattern analysis of the isotope patterns observed for the *N*-terminal fragments of the cleavage of Npβ Dha61 and preSUMO1 Dha51.

Entry	Protein	Charge State	Deviation OH [Da]	Deviation NH_2 [Da]
**1**	**Npβ**	7+	0.1447	0.0054
**2**		8+	0.1285	0.0059
**3**		9+	0.1145	0.0052
**4**	**preSUMO1**	6+	0.1672	0.0059
**5**		7+	0.1465	0.0067
**6**		8+	0.1317	0.0086

Isotopic pattern analysis confirmed the presence of α-amidated *C*-termini for both cases.

3.8 Protein Melting

Melting curves were recorded on a Prometheus NT.48 differential scanning fluorimeter (NanoTemper Technologies, Munich, Germany) for the respective cysteine mutants. Samples were loaded into high sensitivity quartz capillaries. Unfolding was detected during heating using a linear temperature gradient (20°C to 95°C, 1°C/min) and an excitation power of 50% (100% for Npβ). Melting curves were recorded at three different concentrations in phosphate buffer (20 mM NaPi, 50 mM NaF, pH 7.4). The data were analysed using PR.Stability Analysis v1.0.2 software (NanoTemper Technologies, Munich, Germany) and GraphPad Prism 8.0.0 (GraphPad Software Inc., San Diego, USA).

Supplementary Table S6: Melting data for Annexin V and Npβ.

Entry	Protein	T_{ON} [°C]	T_M [°C]
**1**	Annexin V	48.1±0.3	52.1±0.3
**2**	Npβ	34.6±1.6	50.9±0.3

3.9 In-lysate Dha Formation and Cleavage

3.9.1 E. coli BL21 (DE3) Lysate Preparation and Cleavage

E. coli BL21(DE3) were grown in 100 mL of LB media without antibiotics at 37°C for 16 hours. The cells were pelleted and the cell pellets were resuspended and lysed in 5 mL Tris buffer (20 mM Tris, 100 mM NaCl, pH 8.0) with a protease inhibitor cocktail and sonicated using a microtip. The supernatant was separated by centrifugation (20,000 rpm, 10 min at 4°C). 1 mL of supernatant was incubated with 10 mM DTT at room temperature for 30. DTT was removed via desalting into the same buffer using a PD MidiTrap G-25 column (Cytiva). To obtain the dehydroalanine (Dha) mutants of the cysteine-containing proteins, the above desalted solution was incubated with 500 molar equivalents of DBHDA. The mixtures were kept at 37°C for 3 h. After removing the excess DBHDA by desalting into cleavage buffer (10 mM Tris, pH 8.0, PD MidiTrap G-25, Cytiva), the Dha-containing proteins were cleaved at room temperature for 18 h in the presence of 100 mM tetrahydroxydiboron and 25 mM sodium ascorbate. Samples of untreated lysate, Dha mutants and the cleavage reaction mixture were digested with trypsin for MS/MS analysis.

3.9.2 In-lysate Cleavage of FLAG-Histone H3 Dha27 and Dha36

Competent *E. coli* BL21(DE3) cells were transformed with plasmids encoding for Histone eH3.1-K27C or eH3.1-K36C. Cells were cultured in 200 mL of LB media containing 100 µg/mL ampicillin at 37ºC until an OD_{600} of 0.5 was reached. 1 mM IPTG was added to induce protein expression. After 4 h of incubation at 37ºC, cell pellets were harvested and resuspended in 20 mL of wash buffer (50 mM Tris, 100 mM NaCl, pH 8.0) supplemented with cOmplete™ Mini protease inhibitor cocktail (Roche). After sonication with a microtip and centrifugation (20 min, 20,000 rpm at 4ºC), the supernatant was discarded. The cell pellets were incubated with 0.3 mL of DMSO for 10 min at room temperature followed by the addition of 3 mL of unfolding buffer (7 M GdnHCl, 20 mM Tris, 10 mM DTT, pH 7.5) and vigorous shaking for 1 h. The supernatant was collected after centrifugation for later cleavage experiments. In general, 1 mL of the supernatant was desalted into DBHDA reaction buffer (3 M GdnHCl, 100 mM sodium phosphate, pH 8.0) using a PD MidiTrap G-25 column (Cytiva). 500 molar equivalents of DBHDA were added immediately to the desalted solution, and the mixture was incubated at 37ºC for 3 h to yield the Dha mutants eH3.1-K27Dha or eH3.1-K36Dha. The

solution was desalted into cleavage buffer (10 mM Tris, pH 8.0) using PD MidiTrap G-25 columns (Cytiva). Then 100 mM tetrahydroxydiboron and 25 mM sodium ascorbate were added and the mixture was incubated at 25°C for 20 h. Standards of purified eH3 K27Dha and K36Dha (0.1 mg/mL in H_2O) were also cleaved under the same conditions for Western blot comparison. Aliquots of the lysates containing Histone eH3.1-K27 mutants were digested with chymotrypsin for LC-MS/MS analysis.

Western blot analysis

Samples were incubated with 4 × Laemmli buffer (5 μL) (BME free) and heated to 95 °C (10 min).

Samples were analysed by SDS-PAGE (MES running buffer, 12% bis-TRIS precast gel, 90-150 V, 1.5 h, room temperature) and Western blot. Western blot analysis was conducted using an alkaline phosphatase fused, primary mouse anti-HA monoclonal antibody (1:2000 dilution, Sigma Aldrich, Catalogue No. A5477). NBT/BCIP substrate solution (Sigma Aldrich, Catalogue No. B1911) was used to visualize proteins. Blocking was conducted using 5% (w/v) BSA in TBS buffer, with washes carried out using TBS and TBS-T buffer.

3.10 In-lysate Production of Neurokinin A

Plasmid Preparation

The human histone *eH3.1 (C96A/C110A)* gene with a *C*-terminal FLAG and HA tag was subcloned into a pET3d vector using NcoI and BamHI sites (Thermofischer, GeneArt service). The eH3.1-K27C and eH3.1-K36C mutants were generated by site-directed mutagenesis (QuickChange II site-directed mutagenesis kit, Agilent). The gene *NKA-eH3.1* encoding the neuropeptide Neurokinin A (NKA) was constructed by fusion with the *N*-terminus of histone eH3.1 using the In-Fusion cloning kit (Takara Bio). A cysteine residue was inserted between the *NKA* and *eH3.1* gene as a handle for Dha formation and cleavage.

eH3.1

ATGGCACGTACCAAACAGACCGCACGTAAAAGCACCGGTGGTAAAGCACCGCGTAAACAGCT
GGCAACCAAAGCAGCCCGTAAAAGCGCACCGGCAACCGGTGGTGTTAAAAAACCGCATCGTT
ATCGTCCGGGTACAGTTGCACTGCGTGAAATTCGTCGTTATCAGAAAAGTACCGAACTGCTG
ATTCGTAAACTGCCGTTTCAGCGTCTGGTTCGTGAAATTGCACAGGATTTCAAAACCGATCT
GCGTTTTCAGAGCAGCGCAGTTATGGCACTGCAAGAAGCAGCAGAAGCATATCTGGTTGGCC
TGTTTGAAGATACCAATCTGGCAGCAATTCATGCAAAACGTGTTACCATTATGCCGAAAGAT
ATTCAGCTGGCACGTCGTATTCGTGGTGAACGTGCCGGTGGTGATTATAAAGATGATGATGA
TAAAAGTGCAGCCGGTGGTTATCCGTATGATGTTCCGGATTATGCCTAA

eH3.1-K27C

ATGGCACGTACCAAACAGACCGCACGTAAAAGCACCGGTGGTAAAGCACCGCGTAAACAGCT
GGCAACCAAAGCAGCCCGTTGCAGCGCACCGGCAACCGGTGGTGTTAAAAAACCGCATCGTT
ATCGTCCGGGTACAGTTGCACTGCGTGAAATTCGTCGTTATCAGAAAAGTACCGAACTGCTG
ATTCGTAAACTGCCGTTTCAGCGTCTGGTTCGTGAAATTGCACAGGATTTCAAAACCGATCT
GCGTTTTCAGAGCAGCGCAGTTATGGCACTGCAAGAAGCAGCAGAAGCATATCTGGTTGGCC
TGTTTGAAGATACCAATCTGGCAGCAATTCATGCAAAACGTGTTACCATTATGCCGAAAGAT
ATTCAGCTGGCACGTCGTATTCGTGGTGAACGTGCCGGTGGTGATTATAAAGATGATGATGA
TAAAAGTGCAGCCGGTGGTTATCCGTATGATGTTCCGGATTATGCCTAA

eH3.1-K36C

ATGGCACGTACCAAACAGACCGCACGTAAAAGCACCGGTGGTAAAGCACCGCGTAAACAGCT
GGCAACCAAAGCAGCCCGTAAAAGCGCACCGGCAACCGGTGGTGTTTGCAAACCGCATCGTT
ATCGTCCGGGTACAGTTGCACTGCGTGAAATTCGTCGTTATCAGAAAAGTACCGAACTGCTG

ATTCGTAAACTGCCGTTTCAGCGTCTGGTTCGTGAAATTGCACAGGATTTCAAAACCGATCT
GCGTTTTCAGAGCAGCGCAGTTATGGCACTGCAAGAAGCAGCAGAAGCATATCTGGTTGGCC
TGTTTGAAGATACCAATCTGGCAGCAATTCATGCAAAACGTGTTACCATTATGCCGAAAGAT
ATTCAGCTGGCACGTCGTATTCGTGGTGAACGTGCCGGTGGTGATTATAAAGATGATGATGA
TAAAAGTGCAGCCGGTGGTTATCCGTATGATGTTCCGGATTATGCCTAA

neurokinin A

CATAAAACTGACTCGTTCGTCGGCCTGATG

NKA-eH3.1

ATGCATAAAACTGACTCGTTCGTCGGCCTGATGTGCGCACGTACCAAACAGACCGCACGTAA
AAGCACCGGTGGTAAAGCACCGCGTAAACAGCTGGCAACCAAAGCAGCCCGTAAAAGCGCAC
CGGCAACCGGTGGTGTTAAAAAACCGCATCGTTATCGTCCGGGTACAGTTGCACTGCGTGAA
ATTCGTCGTTATCAGAAAAGTACCGAACTGCTGATTCGTAAACTGCCGTTTCAGCGTCTGGT
TCGTGAAATTGCACAGGATTTCAAAACCGATCTGCGTTTTCAGAGCAGCGCAGTTATGGCAC
TGCAAGAAGCAGCAGAAGCATATCTGGTTGGCCTGTTTGAAGATACCAATCTGGCAGCAATT
CATGCAAAACGTGTTACCATTATGCCGAAAGATATTCAGCTGGCACGTCGTATTCGTGGTGA
ACGTGCCGGTGGTGATTATAAAGATGATGATGATAAAAGTGCAGCCGGTGGTTATCCGTATG
ATGTTCCGGATTATGCCCATCATCATCATCATCATCATCACTAATAA

Primers

NKA-eH3.1 Forward
5′- TGACTCGTTCGTCGGCCTGATGTGCGCACGTACCAAACAGACC-3′

NKA-eH3.1 Reverse
5′- CCGACGAACGAGTCAGTTTTATGCATGGTATATCTCCTTCTTAAAGT-3′

Expression of NKA-eH3.1 and Lysate Preparation

The constructed NKA-eH3.1 plasmid was used to transform *E. coli* BL21(DE3) competent cells and expressed as Histone H3 with the *N*-terminally fused neuropeptide Neurokinin A. Cells were grown in 300 mL of LB media (supplemented with 100 µg/ml ampicillin) at 37ºC and induced with 1 mM IPTG when an OD_{600} of 0.6 was reached. The incubation was continued for another 3 h at the same temperature. Cell pellets were resuspended in 20 mL of wash buffer

(50 mM Tris, 100 mM NaCl, pH 8.0) with supplemented with cOmplete™ Mini protease inhibitor cocktail (Roche) and sonicated using a microtip. The supernatant was separated by centrifugation (20,000 rpm, 20 min at 4°C) and discarded. To dissolve histone proteins, the cell pellets were mixed with 0.5 mL of DMSO at room temperature for 10 min before being shaken with 5 mL of unfolding buffer (7 M GdnHCl, 20 mM Tris, 10 mM DTT, pH 7.5) for 1 h. The mixture was centrifuged at 20,000 rpm for 10 min at room temperature. The supernatant was desalted into DBHDA reaction buffer (3 M GdnHCl, 100 mM sodium phosphate, pH 8.0) via PD-10 columns (Cytiva). After measuring the concentration of the protein mixtures in *E. coli* lysates by BCA assay (Pierce™ BCA protein assay kit, Thermo Fisher Scientific), 250 molar equivalents of DBHDA were added. The mixture was incubated at 37°C for 3 h to form NKA(Dha)-eH3.1, and desalted into cleavage buffer (10 mM Tris, pH 8.0) to remove excess DBHDA using PD-10 columns (Cytiva). The diboron cleavage was proceeded in the presence of 100 mM tetrahydroxydiboron and 25 mM sodium ascorbate. A 100 μg aliquot from each reaction solution (Cys, Dha and cleavage) was digested with trypsin for LC-MS/MS analysis. The remaining cleaved lysate was lyophilized on a freeze dryer (Christ Alpha 204 LSCbasic) for later assays.

Amino acid sequence

NKA-eH3.1

```
HKTDSFVGLMCARTKQTARKSTGGKAPRKQLATKAARKSAPATGGVKKPHRYRPGTVALREI
RRYQKSTELLIRKLPFQRLVREIAQDFKTDLRFQSSAVMALQEAAEAYLVGLFEDTNLAAIH
AKRVTIMPKDIQLARRIRGERAGGDYKDDDDKSAAGGYPYDVPDYAHHHHHHHH
```

Ex vivo characterization of Neurokinin A containing lysate

The ability of the lysates to contract rat uterus smooth muscle were test in an organ bath with a chamber volume of 10 mL. Briefly, individual 15 mm-long sections of uterus from three non-pregnant Wistar rats (250 g) were suspended in modified physiological Kreb's solution containing low calcium (1.26 mM) and low glucose (2.8 mM) at 25°C to minimise the spontaneous activity. Each uterus was tensioned to 1 g and the viability of the muscles was tested with 0.3 nM 5-hydroxytryptamine (5HT). The contractile response of the tissue to the sample preparation or Neurokinin A (NKA) standard (100 μM final concentration) was compared. In each case, the tissue was allowed to stabilize before adding the test compounds and effect of the sample preparation was tested in three independent uterus preparations. The

response of the tissue was recorded using PowerLab data acquisition system (ADI with a bridge amplifier, a calibrated force (g) transducers) and LabChart software (v8.1.19). The response was monitored for 30 seconds after the addition of each challenge and the tissue was allowed to recover for at least 2 minutes before the application of the next compound/sample.

3.11 LC-MS/MS Analysis of in-Lysate Dha Formation and Cleavage

Sample Preparation

'In-solution' tryptic or chymotryptic digestions were performed following an MS-compatible protocol. Specifically, 100 µg of cell lysate was diluted to 100 µL with 100 mM triethylammonium bicarbonate (TEAB) buffer containing 8 M urea and treated with 10 mM TCEP at room temperature for 30 min. The mixture was incubated with 50 mM of freshly prepared 2-chloroacetamide solution at room temperature for 30 min in the dark. Then a tenfold dilution was performed with a 50 mM TEAB buffer. The mixture was treated with trypsin (1:20 w/w trypsin:protein), or with 10 mM $CaCl_2$ and chymotrypsin (1:20 w/w chymotrypsin:protein) at 37°C overnight. The peptides were purified using an Oasis HLB 1cc Vac cartridge (Waters) and the peptide fragments were eluted with 250 µL of eluent (0.1% TFA/50% acetonitrile or 0.1% TFA/80% acetonitrile in milliQ water). The eluted peptides were concentrated overnight on a centrifugal evaporator (EZ-2.3 Elite, GeneVac SP).

Data Acquisition

Analysis of peptides was carried out using an Ultimate 3000 nano-LC 1000 system coupled to an Orbitrap Exploris 480 (Thermo Fisher Scientific). Dried samples were resuspended in ultra-pure water with 5% formic acid and 5% DMSO. Peptides were initially trapped on a C18 PepMap100 pre-column (300 µm inner diameter x 5 mm, 100A) and then separated on an in-house constructed C18 column (Reprosil-Gold, Dr. Maisch, 1.9 µm particle size) column (ID: 75 µm, length: 50 cm) at a flow rate of 200 nL/min. Peptides were either separated over 60 min (12-35%B) or 120 min (12-35%B) using mobile phase A (water and 0.1% formic acid) and mobile phase B (acetonitrile and 0.1% formic acid). Separated peptides were directly electrosprayed into an Orbitrap Exploris 480 mass spectrometer (Thermo Fisher Scientific). Mass spectra were acquired in the orbitrap (350-1400 m/z, resolution 60000, AGC target 3 x 106, maximum injection time 50 ms) in a data-dependent mode. The top 40 most abundant peaks in the survey scan were fragmented using CID (resolution 7500, AGC target 4 x104, maximum injection time 64 ms).

Data Analysis

Protein identification and quantification were performed using Andromeda search engine implemented in MaxQuant (2.0.1.0).*(80)* Peptides were searched against reference database (uniport proteome UP000000625, downloaded 19.05.2021) with additional sequence of recombinant protein provided below. Default settings were used apart from the following changes: semi-tryptic search was performed with the following variable modifications: cysteine carbamidomethylation, *N*-teminal acetylation, methionine oxidation, Cysteine → Dha (custom defined −33.9877 Da), *C*-terminal amidation (every peptide), Cysteine → lactamide (after cleavage) (custom defined −30.9881 Da). Up to four modifications were allowed per peptide. Additionally, match between runs function was enabled. As di-boron treated sampled had significantly less histone protein than control, the intensity of modified peptides was normalised based on sum of intensity of the TDSFVGLMCAR peptide which was not converted to Dha (carbamidomethylated Cys alone or together with oxidation of methionine).

3.12 Synthetic Chemistry

3.12.1 General Methodology

All chemical reactions were conducted under ambient conditions unless noted otherwise. Glassware for reactions that were conducted under inert atmosphere was thoroughly dried prior to use by heating *in vacuo*. Syringes used for the addition of reagents and solvents to reactions under inert atmosphere were purged with dry nitrogen thrice prior to use and airtight rubber septa were employed. Solvents were removed on a rotary evaporator where applicable. Chemical reagents were purchased from commercial suppliers (Alfa Aesar, Combi-Blocks, Fluorochem, Fisher Scientific, Sigma Aldrich, VWR) and used without further purification unless noted otherwise. DBHDA = 2,5-dibromohexanediamide; MDBPA = methyl 2,5-dibromopentanoate.

Chromatography

Chromatographic separations were performed by flash chromatography on CombiFlash instruments (Teledyne ISCO, USA). Analytical thin-layer chromatography (TLC) was performed on Merck aluminium backed sheets coated with silica gel 60 F_{254}. UV-active substances were visualized by short wavelength (254 nm) ultraviolet light or using a $KMnO_4$ staining solution and heating the TLC plate after application.

3.12.2 Analytical Techniques

3.12.2.1 Nuclear magnetic resonance spectroscopy (NMR spectroscopy)

Synthesized compounds were characterized by NMR spectroscopy. All NMR spectra were recorded using Bruker Advance-III HD 400 MHz, 500 MHz and 600 MHz spectrometers. ^{1}H- and ^{13}C-NMR spectra were referenced to the NMR solvent shift. Chemical shifts are given in parts per million (ppm) and coupling constants are declared in Hertz (Hz). The multiplicities of the signals are reported as singlet (s), doublet (d), triplet (t), quartet (q), pentet (pent), multiplet (m), broad (br) or any combination of these. Signals showing unexpected multiplicities are indicated by the prefix “app”.

3.12.2.2 IR spectrometry

IR spectra were recorded on a Bruker Tensor 27 Fourier Transform spectrophotometer equipped with a diamond ATR probe.

3.12.2.3 Melting Points

Uncorrected melting points were determined using a Leica Galen III microscope equipped with a heating stage and a Testo 720 thermocouple probe. Heating was conducted up to 200°C at a constant heating rate of 2°C/minute.

3.12.3 Small-molecule Synthetic Procedures

3.12.3.1 Tetradeuteroxydiboron ($B_2(OD)_4$) (**S1**)

Tetradeuteroxydiboron ($B_2(OD)_4$) was synthesized following a literature procedure.*(12)*

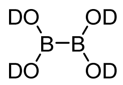

A 50 mL Schlenk flask was equipped with a dropping funnel and evacuated. The flask was thoroughly heated with a heat gun and allowed to cool under vacuum before being filled with N_2. Tetrakis(dimethylamino)diboron (3 mL, 13.0 mmol, 1.00 equiv) and D_2O (99.8% D, 5 mL) were added and the flask was cooled to 0°C. Deuterium chloride (35% wt in D_2O, 99% D) (15 mL) was added dropwise via the dropping funnel over 20 min leading to formation of a white precipitate. The mixture was allowed to warm to room temperature and stirred for an additional hour. The precipitate was collected by filtration, washed with 3 mL of 10% DCl in D_2O and 3 mL of $CDCl_3$ and dried *in vacuo* to yield the title compound as a white solid (854 mg, 9.09 mmol, 70%).

98% deuterium incorporation was determined by ^{1}H-NMR in DMSO-d_6 against mesitylene as internal standard.

^{1}H-NMR (400 MHz, DMSO-d_6, 300 K): δ (ppm) = 8.66 (s, 0.008 H), 7.62 (s, 0.013 H).

^{11}B-NMR (128 MHz, DMSO-d_6, 300 K): δ (ppm) = 31.1 (s).

The NMR data are in agreement with previous literature reports.*(12)*

3.12.3.2 Synthesis of Ac-Gly-Dha-NHBn **1**

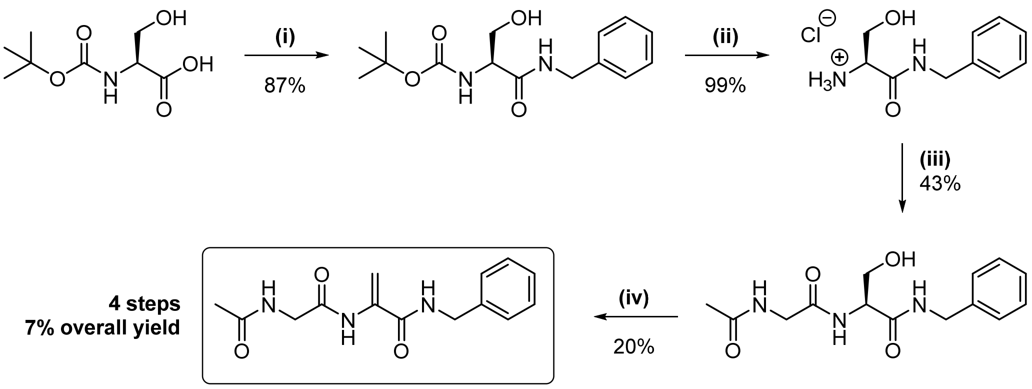

Supplementary Figure S59: Model substrate Ac-Gly-Dha-NHBn (1) was synthesized from commercial starting materials in four steps.

Boc-Ser-NHBn (S2)

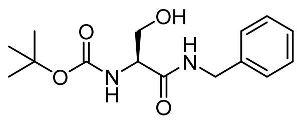

Boc-Ser-OH (1.00 g, 4.87 mmol, 1.00 equiv) and *N*-methylmorpholine (589 µL, 5.36 mmol, 1.10 equiv) were dissolved in EtOAc (30 mL). The solution was cooled to −10°C before isobutyl chloroformate (732 µL, 5.36 mmol, 1.10 equiv) and a solution of benzylamine (586 µL, 5.36 mmol, 1.10 equiv) in EtOAc (10 mL) were added subsequently. The mixture was kept at 10 to 15°C for 2 hours, water was added (20 mL) and stirring continued for 10 min. The organic layer was separated and washed with 1 M HCl (20 mL), saturated aqueous Na_2CO_3 solution (20 mL) and brine (20 mL) and the solvent was evaporated. The residue was titurated with petroleum ether and dried *in vacuo* to yield the title compound as a white solid (1.25 g, 4.23 mmol, 87%).

^{1}H-NMR (400 MHz, DMSO-d_6, 300 K): δ (ppm) = 8.30 (t, J = 5.8 Hz, 1H, CON*H*), 7.33 – 7.17 (m, 5H, Ar-*H*), 6.65 (d, J = 7.9 Hz, 1H, CON*H*), 4.84 (t, J = 5.7 Hz, 1H, O*H*), 4.29 (app qd, J = 15.3, 5.9 Hz, 2H, CH_2), 4.05 – 3.96 (m, 1H, C*H*), 3.64 – 3.51 (m, 2H, CH_2), 1.39 (s, 9H, CH_3).

^{13}C-NMR (101 MHz, DMSO-d_6, 300 K): δ (ppm) = 170.5 (*C*=O), 155.2 (*C*=O), 139.4 (C_{Ar}), 128.1 (C_{Ar}), 126.9 (C_{Ar}),126.6 (C_{Ar}), 78.2 (C_{quart}), 61.8 (CH_2), 57.0 (*C*H), 42.0 (CH_2), 28.2 (CH_3).

The NMR data are in agreement with literature values.*(81)*

H-Ser-NHBn Hydrochloride (S3)

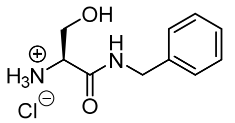

Boc-Ser-NHBn (3.75 g, 12.7 mmol, 1.00 equiv) was dissolved in DCM (120 mL). HCl (4 M in dioxane, 31.9 mL, 127 mmol, 10.0 equiv) was added and the mixture was stirred at room temperature for an hour. The solvent was evaporated and the title compound was afforded as a white solid (2.92 g, 12.6 mmol, 99%).

^{1}H-NMR (400 MHz, DMSO-*d6*, 300 K): δ (ppm) = 9.09 (t, *J* = 5.9 Hz, 1H, CON*H*), 8.29 (s, 3H, N*H3*Cl), 7.35 – 7.22 (m, 5H, Ar-*H*), 5.55 (t, *J* = 4.9 Hz, 1H, O*H*), 4.34 (d, *J* = 5.9 Hz, 2H, C*H2*), 3.88 (t, *J* = 5.0 Hz, 1H, C*H*), 3.85 – 3.73 (m, 2H, C*H2*).

^{13}C-NMR (101 MHz, DMSO-*d6*, 300 K): δ (ppm) = 166.8 (*C*=O), 138.7 (*C*Ar), 128.3 (*C*Ar), 127.2 (*C*Ar),126.9 (*C*Ar), 60.3 (*C*H2), 54.4 (*C*H), 42.2 (*C*H2).

The NMR data are in agreement with literature values.*(82)*

Ac-Gly-Ser-NHBn (S4)

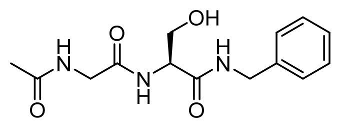

N-acetyl glycine (1.02 g, 8.67 mmol, 1.00 equiv), N-methylmorpholine (2.38 mL, 21.7 mmol, 2.50 equiv) and isobutyl chloroformate (1.12 mL, 8.67 mmol, 1.00 equiv) were dissolved in DMF (100 mL). The mixture was stirred at room temperature for 15 min, before H-Ser-NHBn hydrochloride (2.00 g, 8.67 mmol, 1.00 equiv) was added as a solid in one portion. The mixture was stirred overnight and the solvent was evaporated. The crude product was purified by reversed-phase flash column chromatography (C_{18} silica, 0-100% MeCN in H_2O) and lyophilized to yield Ac-Gly-Ser-NHBn as a white solid (1.10 g, 3.75 mmol, 43%).

^{1}H-NMR (400 MHz, DMSO-*d6*, 300 K): δ (ppm) = 8.36 (t, *J* = 6.0 Hz, 1H, CON*H*), 8.13 (t, *J* = 5.6 Hz, 1H, CON*H*), 7.96 (d, *J* = 7.9 Hz, 1H, CON*H*), 7.35 – 7.17 (m, 5H, Ar-*H*), 4.94 (t, *J* = 5.4 Hz, 1H, O*H*), 4.35 – 4.26 (m, 3H), 3.81 – 3.56 (m, 4H), 1.84 (s, 3H, C*H3*).

^{13}C-NMR (101 MHz, DMSO-*d6*, 300 K): δ (ppm) = 169.9 (*C*=O), 169.8 (*C*=O), 169.2 (*C*=O), 139.3 (*C*Ar), 128.2 (*C*Ar), 127.0 (*C*Ar),126.6 (*C*Ar), 61.6 (*C*H2), 55.3 (*C*H), 42.2 (*C*H2), 42.0 (*C*H2), 22.4 (*C*H3).

IR (silicone, neat): $\tilde{\nu}$ (cm^{-1}) = 3273, 1672, 1650, 1562, 1428, 1284, 1092, 1062, 1032, 725, 691.

HRMS (ESI+): *m/z* for $C_{14}H_{20}O_4N_3$ $[M+H]^+$: calculated: 294.1448; found: 294.1449.

m.p.: 184–187°C

Ac-Gly-Dha-NHBn (1)

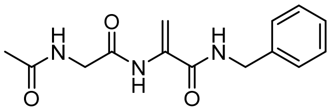

Ac-Gly-Ser-NHBn (200 mg, 0.68 mmol, 1.00 equiv) was dissolved in DMF (5 mL) and cooled to 0°C. MsCl (74 µL, 0.95 mmol, 1.40 equiv) was added followed by Et_3N (237 µL, 1.70 mmol, 2.50 equiv). The mixture was allowed to slowly warm to room temperature and stirred overnight. Another portion of MsCl (74 µL, 0.95 mmol, 1.40 equiv) and Et_3N (237 µL, 1.70 mmol, 2.50 equiv) were added and the mixture was stirred for an additional day. The title compound was obtained after flash chromatography (twice, silica, 0-100% DCM/DCM:MeOH 9:1) as a white solid (37.5 mg, 0.14 mmol, 20%).

^{1}H-NMR (400 MHz, DMSO-d_6, 300 K): δ (ppm) = 9.11 (s, 1H, CON*H*), 8.95 (t, *J* = 6.0 Hz, 1H, CON*H*), 8.29 (t, *J* = 5.9 Hz, 1H, CON*H*), 7.37 – 7.19 (m, 5H, Ar-*H*), 6.13 (s, 1H, C=C*H*), 5.55 (s, 1H, C=C*H*), 4.37 (d, *J* = 6.0 Hz, 2H, CH_2), 3.80 (d, *J* = 5.9 Hz, 2H, CH_2), 1.88 (s, 3H, CH_3).

^{13}C-NMR (101 MHz, DMSO-d_6, 300 K): δ (ppm) = 169.9 (*C*=O), 168.6 (*C*=O), 163.6 (*C*=O), 139.1 (C_{Ar}), 135.0 (*C*=CH_2), 128.3 (C_{Ar}), 127.2 (C_{Ar}), 126.8 (C_{Ar}), 102.8 (C=CH_2), 43.1 (CH_2), 42.6 (CH_2), 22.3 (CH_3).

IR (diamond ATR, neat): $\tilde{\nu}$ (cm^{-1}) = 3275, 1678, 1658, 1625, 1554, 1532, 1305, 1246, 972, 730, 712, 696, 642.

HRMS (ESI+): *m/z* for $C_{14}H_{17}O_3N_3Na$ $[M+Na]^+$: calculated: 298.1162; found: 294.1161.

m.p.: 169–172°C

N-Benzyl-2-oxopropanamide (2)

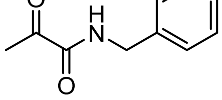

Pyruvic acid (355 µL, 5.04 mmol, 1.10 equiv) was dissolved in DMF (15 mL) and cooled to 0°C. HATU (2.26 g, 5.95 mmol, 1.30 equiv) and DIPEA (2.4 mL, 13.7 mmol, 3.00 equiv) were added subsequently and the mixture was stirred for 10 min. Benzylamine (500 µL, 4.58 mmol, 1.00 equiv) was added dropwise, the solution was allowed to warm to room temperature and stirred for 2 hours. The

mixture was diluted with 1 M HCl (20 mL) and extracted with EtOAc (3 × 20 mL). The crude product was purified by flash chromatography (silica, 0-100% EtOAc in petroleum ether) to yield the title compound as a yellow oil (142 mg, 0.80 mmol, 18%).

^{1}H-NMR (400 MHz, DMSO-d_6, 300 K): δ (ppm) = 9.10 (t, J = 5.8 Hz, 1H, CON*H*), 7.35 – 7.19 (m, 5H, Ar-*H*), 4.31 (d, J = 6.5 Hz, 2H, CH_2), 2.36 (s, 3H, CH_3).

^{13}C-NMR (101 MHz, DMSO-d_6, 300 K): δ (ppm) = 197.2 (*C*=O), 161.2 (*C*=O), 138.7 (C_{Ar}), 128.3 (C_{Ar}), 127.4 (C_{Ar}), 126.9 (C_{Ar}), 42.1 (CH_2), 25.0 (CH_3).

The analytical data are in accordance with the literature.*(83)*

N-Benzyl-2-hydroxypropanamide (3)

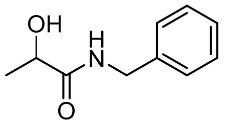

N-Benzyl-2-oxopropanamide (50.0 mg, 0.28 mmol, 1.00 equiv) was dissolved in MeOH (5 mL). To the stirred solution was added $NaBH_4$ (21.4 mg, 0.56 mmol, 2.00 equiv) in one portion and the mixture was stirred for one hour. The solvent was evaporated and the crude product was purified by flash chromatography (silica, 2-10% MeOH in DCM) to yield the title compound as a colorless oil (22.8 mg, 0.13 mmol, 45%).

^{1}H-NMR (400 MHz, DMSO-d_6, 300 K): δ (ppm) = 8.22 (t, J = 6.1 Hz, 1H, CON*H*), 7.36 – 7.17 (m, 5H, Ar-*H*), 5.51 (br s, 1H, O*H*), 4.28 (d, J = 6.3 Hz, 2H, CH_2), 4.01 (q, J = 6.8 Hz, 1H, C*H*), 1.23 (d, J = 6.8 Hz, CH_3).

^{13}C-NMR (101 MHz, DMSO-d_6, 300 K): δ (ppm) = 174.5 (*C*=O), 139.8 (C_{Ar}), 128.2 (C_{Ar}), 127.1 (C_{Ar}), 126.6 (C_{Ar}), 67.3 (*C*H), 41.6 (CH_2), 21.1 (CH_3).

The analytical data are in accordance with the literature.*(84)*

3.13 Peptide Synthesis & Analysis

3.13.1 Synthesis of Pentapeptide 4

Rink Amide Loading. Rink amide resin (200 μmol, 276 mg, maximum loading 0.81 mmol/g) was placed in a fritted syringe and swollen in CH_2Cl_2 (10 mL) for 30 min before being washed with DMF (5×). The resin was then treated with a solution of 20 vol % piperidine in DMF (2 × 3 min) and washed with DMF (5×), CH_2Cl_2 (5×), and DMF (5×) before being shaken with a solution of Fmoc-Thr(O*t*Bu)-OH (317 mg, 800 μmol), HATU (303 mg, 4.0 equiv, 800 μmol) and *i*Pr2NEt (278 μL, 8.0 equiv, 1.6 mmol) in DMF (4 mL) at room temperature for 2.5 h. The solution was ejected, and the coupling was repeated a second time with the same amounts as listed previously. The resin was then washed DMF (5×) and CH_2Cl_2 (5×) and then treated with a solution of pyridine:Ac_2O (v/v, 9:1, 5 mL) for 10 min. The resin was then washed with DMF (5×), CH_2Cl_2 (5×), and DMF (5×).

Fmoc Deprotection. A given resin-bound peptide was washed with CH_2Cl_2 (5×) and DMF (5×) before being treated with a solution of 20 vol % piperidine in DMF (2 × 3 min). The resin was again washed with DMF (5×), CH_2Cl_2 (5×), and DMF (5×).

PyBOP Coupling Conditions. A given resin-bound peptide was washed with CH_2Cl_2 (5×) and DMF (5×) before being treated with a solution of 20 vol % piperidine in DMF (2 × 3 min). The resin was again washed with DMF (5×), CH_2Cl_2 (5×), and DMF (5×). The resin was shaken for 1 h at room temperature with a solution of the desired Fmoc-protected amino acid (4 equiv), PyBOP (4 equiv), and 4-methylmorpholine (NMM) (8 equiv) in DMF (0.1 M in regard to loaded peptide). The coupling solution was discharged and the resin washed with DMF (5×), CH_2Cl_2 (5×), and DMF (5×).

Rink Amide Resin Cleavage Conditions. Resin-bound peptide was washed with CH_2Cl_2 (5×) and DMF (5×) before being treated with a solution of 20 vol % piperidine in DMF (2 × 3 min). The resin was again washed with DMF (5×), CH_2Cl_2 (5×) and DMF (5×) and then treated with pyridine:Ac_2O (v/v, 9:1, 5 mL) for 10 min to afford a capped N-terminus. The resin was then washed with DMF (5×), CH_2Cl_2 (5×), DMF (5×) and CH_2Cl_2 (20×). The resin was treated with a solution of TFA:*i*Pr_3SiH:H_2O:ethandithiol (94:1:2.5:2.5, v/v/v) and then concentrated under

a stream of N_2 and dried *in vacuo*. The peptide was then precipitated from ice-cold Et_2O, and centrifuged to pellet the desired peptide product.

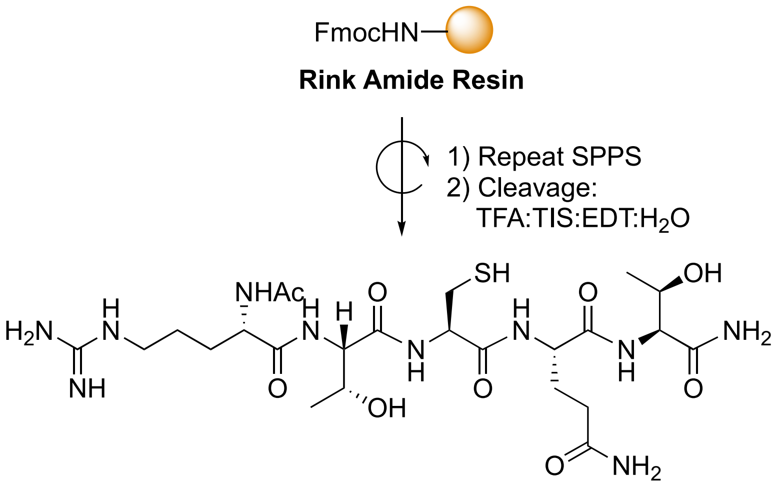

According to the general peptide methods listed – the pentapeptide AcNH-Arg-Thr-Cys-Gln-Thr-NH_2 was synthesised via iterative SPPS on 200 µmol scale to yield 185 mg of crude peptide.

Dha Formation

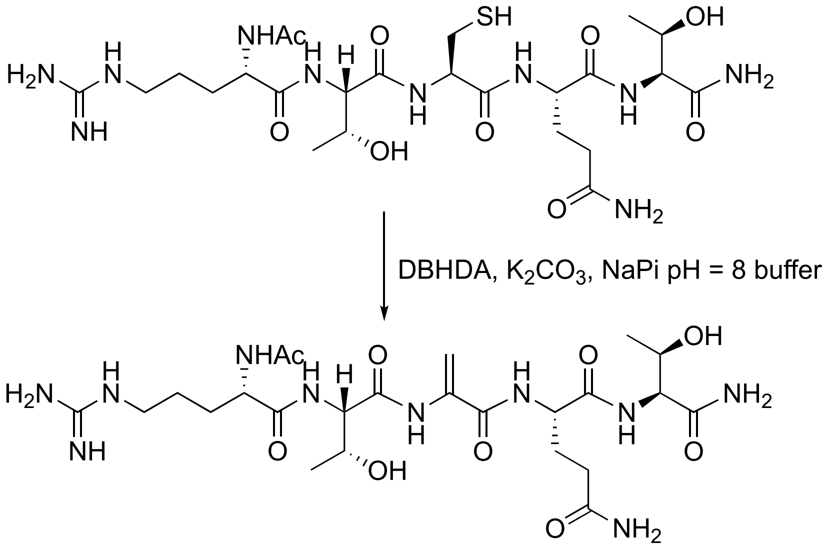

Crude cysteine containing peptide (50 mg, 0.067 mmol – assuming mass of mono TFA salt) was dissolved in buffer (NaP_i, 50 mM, pH 8). Potassium carbonate (18 mg, 0.134 mmol,

2 equiv) was added followed by the addition of DBHDA (100 mg, 0.33 mmol, 5 equiv) in DMSO (300 μmol). The solution was mixed at 25 °C for 1 h, and then 37 °C for 1.5 h. The mixture was centrifuged to remove precipitate, filtered and purified by preparative reverse-phase HPLC (0-20% B over 9.0 mins; A = H_2O, B = MeCN, +0.1% formic acid; flow rate 25 ml min^{-1}; Waters XBridge C18 5 μm OBD, 19 x 100 mm). Fractions containing the product were pooled and lyophilised to yield the desired peptide (7.06 mg, 11 μmol, 20% from resin).

Cysteine alkylation

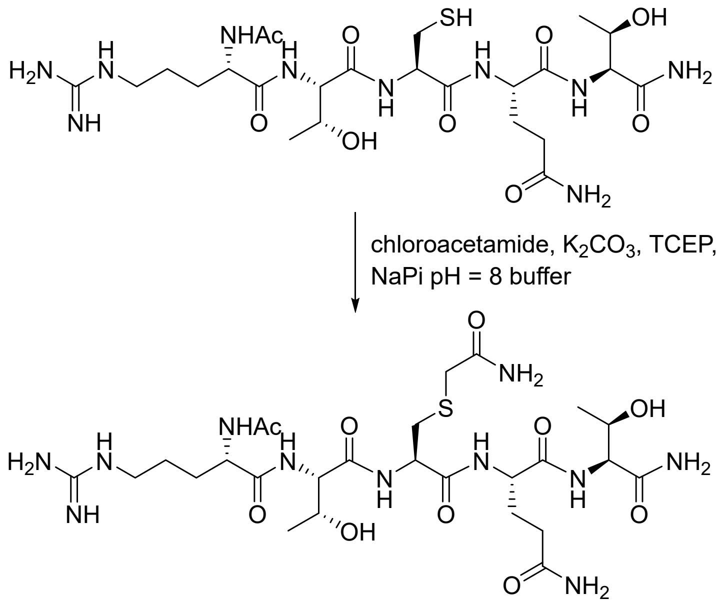

Crude cysteine containing peptide (20 mg, 0.027 mmol – assuming mass of mono TFA salt) was dissolved in 1 mL buffer (NaPi, 50 mM, pH = 8). Potassium carbonate (11 mg, 0.081 mmol, 3 equiv) was added followed by the addition of TCEP (80 μL, 0.041 mmol, 1.5 equiv, 0.5 M in H_2O). Chloroacetamide (13 mg, 0.135 mmol, 5 equiv) was then added and the solution was mixed at 25 °C for 30 min. The mixture was then filtered and purified by preparative reverse-phase HPLC (0-20% B over 9.0 mins; A = H_2O, B = MeCN, +0.1% formic acid; flow rate 25 ml min^{-1}; Waters XBridge C18 5 μm OBD, 19 x 100 mm). Fractions containing the product were pooled and lyophilised to yield the desired peptide (7.2 mg, 9.5 μmol, 35% from resin). The product peptide was used for NMR analysis.

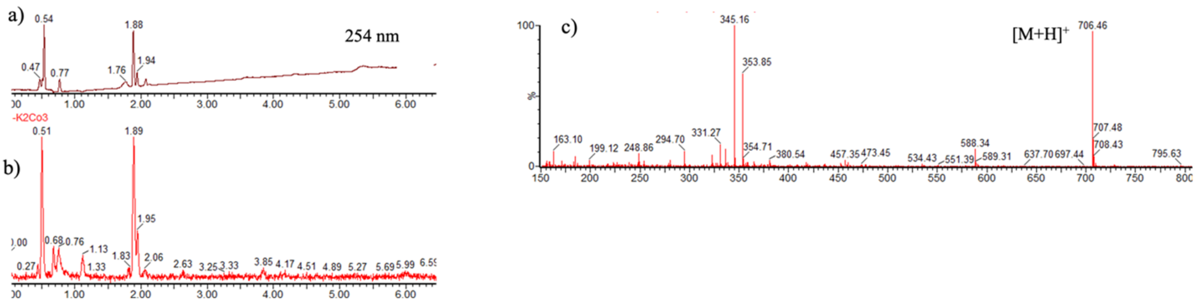

Supplementary Figure S60: LCMS analysis of crude alkylated Cys peptide AcNH-Arg-Thr-Cys(CH_2CONH_2)-Gln-Thr-NH_2 shows expected peptide mass. a) UV trace at 254 nm – product peak at 1.88 min b) TIC trace c) extracted MS of peak at 1.88 min - calculated mass: 706.3 g/mol; observed mass: 706.5 g/mol.

3.13.2 Peptide Analysis

Analysis of the peptide and fractions was carried out on a Waters Acquity UPLC bearing a ELS detector, PDA detector, and coupled Waters SQ Detector 2 mass spec. Samples were run at (0-20% B over 5.4 mins; A = H_2O, B = MeCN, +0.1% formic acid; flow rate 0.6 ml min^{-1}; Waters Acquity UPLC BEH C18 1.7 µm, 2.1 x 100 mm).

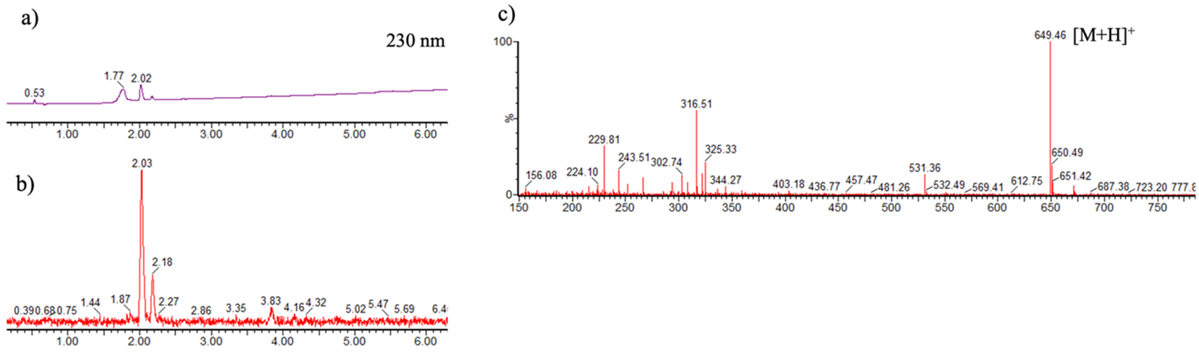

Supplementary Figure S61: LCMS analysis of crude peptide AcNH-Arg-Thr-Cys-Gln-Thr-NH_2 shows expected peptide mass. a) UV trace at 230 nm – product peak at 2.02 min b) TIC trace c) extracted MS of peak at 2.03 min - calculated mass: 649.7 g/mol; observed mass: 649.5 g/mol.

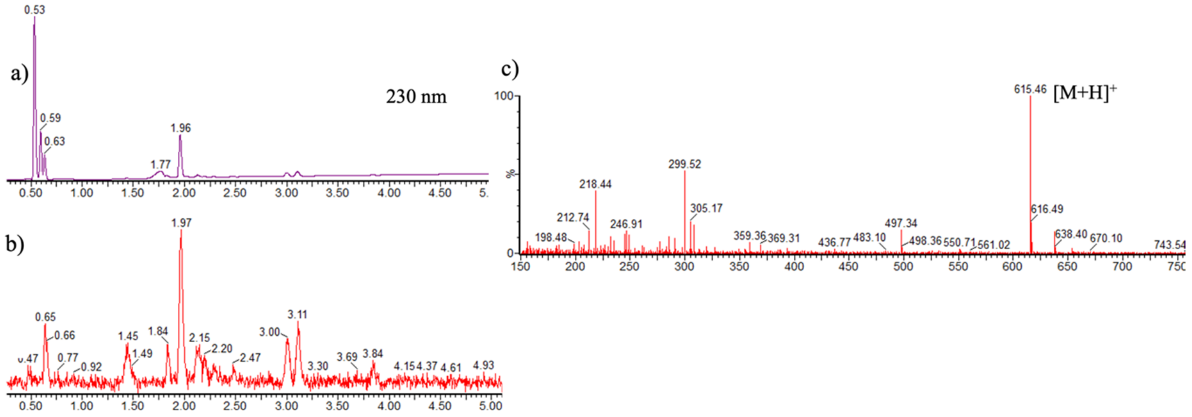

Supplementary Figure S62: LCMS analysis of crude Dha peptide AcNH-Arg-Thr-Dha-Gln-Thr-NH_2 shows expected peptide mass. a) UV trace at 230 nm – product peak at 1.96 min b) TIC trace c) extracted MS of peak at 1.97 min - calculated mass: 615.7 g/mol; observed mass: 615.5 g/mol.

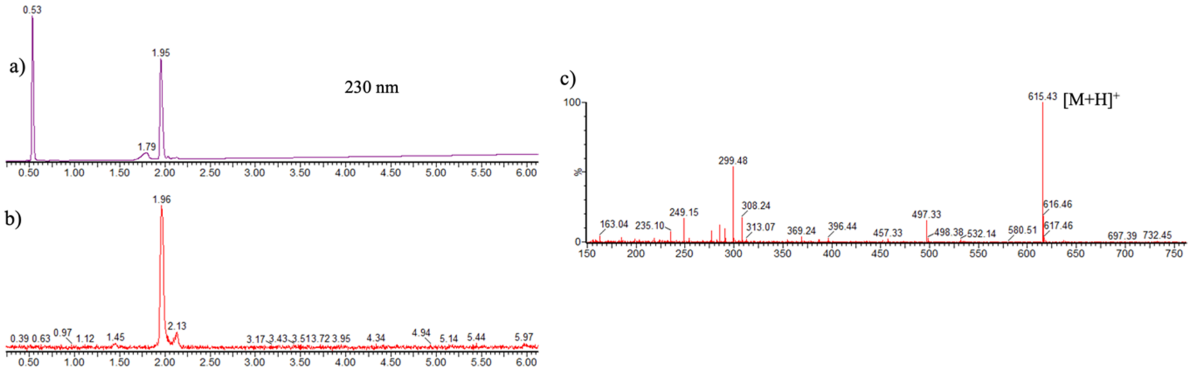

Supplementary Figure S63: LCMS analysis of purified Dha peptide AcNH-Arg-Thr-Dha-Gln-Thr-NH_2: shows expected peptide mass. a) UV trace at 230 nm – product peak at 1.95 min b) TIC trace c) extracted MS of peak at 1.96 min - calculated mass: 615.7 g/mol; observed mass: 615.4 g/mol.

3.14 Reduction of *N*-Benzyl-2-oxopropanamide with tetrahydroxydiboron

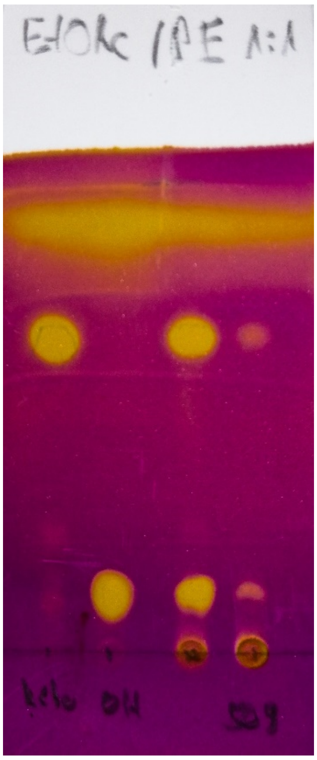

Supplementary Figure S64: TLC analysis shows the reduction of *N*-Benzyl-2-oxopropanamide (2) with tetrahydroxydiboron. From left to right: *N*-Benzyl-2-oxopropanamide (**2**); N-benzyl-2-hydroxypropanamide (**3**), reference compound; co-spot with reaction mixture; reaction mixture.

N-Benzyl-2-oxopropanamide (**2**) (5.0 mg, 28.2 µmol, 1.00 equiv) was dissolved in water (2 mL). Tetrahydroxydiboron (25.3 mg, 282 µmol, 10.0 equiv) was added and the mixture was stirred at room temperature. After 24 h, the mixture was analysed by TLC (EtOAc/petroleum ether 1:1, $KMnO_4$ staining). Comparison with starting material and authentic reference confirmed formation of *N*-Benzyl-2-hydroxypropanamide (**3**).

3.15 Cleavage of Model Substrate Ac-Gly-Dha-NHBn

Ac-Gly-Dha-NHBn (**1**) (100 mg, 0.36 mmol, 1.00 equiv) was suspended in water (30 mL). Tetrahydroxydiboron (325 mg, 3.63 mmol, 10.0 equiv) was added and the mixture was vortexed for 5 min. The mixture was stirred at room temperature for 24 h, lyophilized and purified by column chromatography (0-10% MeOH in DCM). *N*-acetylglycine (5.2 mg, 40.4 µmol, 12%) and *N*-acetylglycinamide (5.9 mg, 50.8 µmol, 14%) were isolated and identified by ^{1}H-NMR analysis and comparison with authentic references.

3.16 Cleavage of Peptides

To a 1 mM solution of pentapeptide above (0.5 mg, 0.76 µmol) in buffer (0.75 mL, NaPi, 10 mM, pH 8) was added tetrahydroxydiboron (1.35 mg, 15.2 µmol) and sodium ascorbate (0.75 mg, 3.8 µmol). The mixture was incubated at 25 °C for 20 h before being lyophilised and then dissolved in D_2O for NMR analysis.

Note – this was repeated using buffer made up in D_2O and this sample was also analysed by NMR and MS.

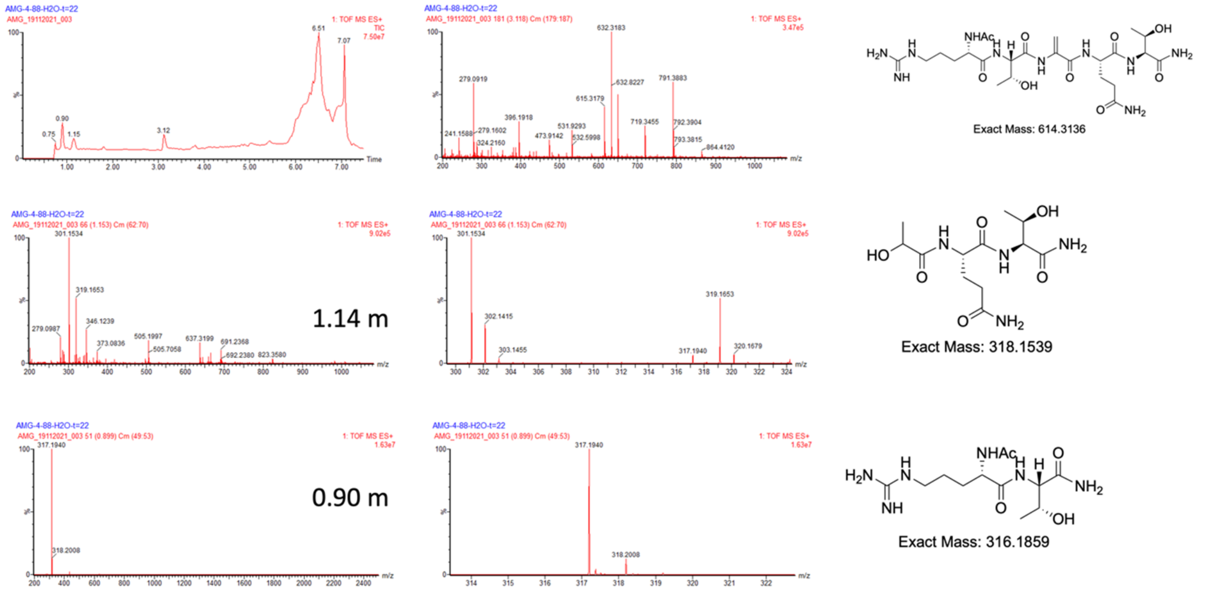

Supplementary Figure S65: LCMS analysis of crude cleavage reaction run in H_2O allows the clear observation of the mass of the two fragments.

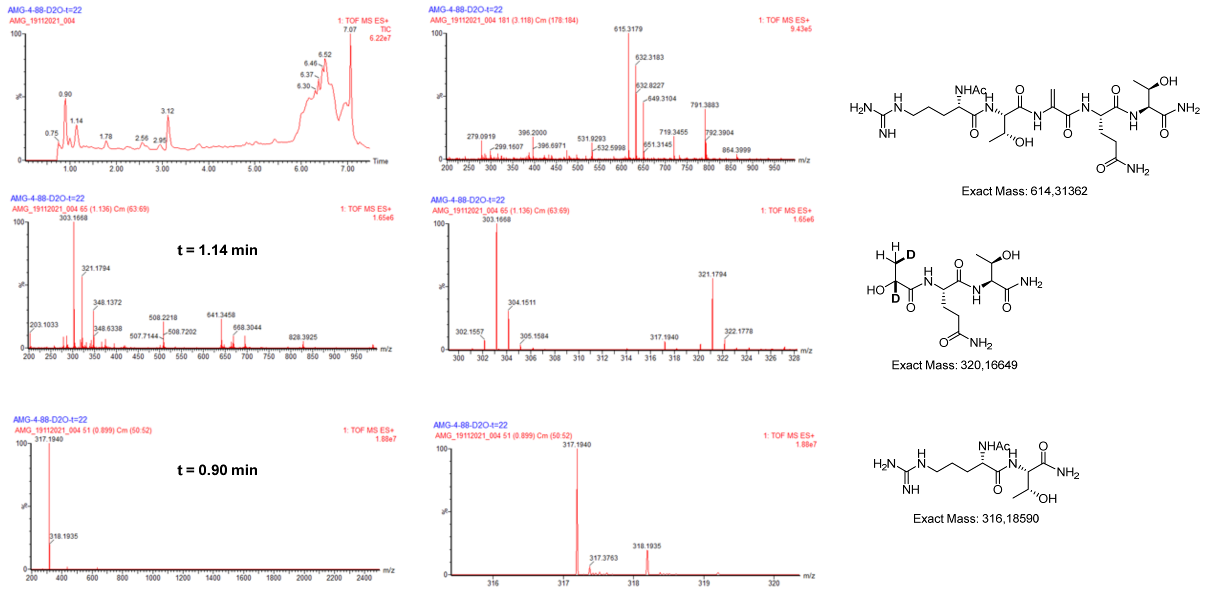

Supplementary Figure S66: LCMS analysis of crude cleavage reaction run in H_2O allows the clear observation of the mass of the two fragments. The *C*-terminal fragment clearly shows a mass increase by two mass units.

3.17 Comparison of Cleavage Rates between Protein and Peptides

Stock solutions of $B_2(OH)_4$ (8.9 mg/mL, 100 mM, in 10 mM NaPi pH 8) and sodium ascorbate (9.9 mg/mL, 50 mM) were freshly prepared. Stock solutions of substrates were prepared as follows: dipeptide **1** (5 mM in DMSO), pentapeptide **4** (10 mM in H_2O), Histone H3 K9Dha (330 μM in H_2O) and Histone eH3.1 K4Dha (345 μM in H_2O). For each substrate, 3 independent experiments were prepared with the following final concentrations – substrate (50 μM), $B_2(OH)_4$ (10 mM, 200 equiv), sodium ascorbate (2.5 mM, 50 equiv) and made up to the final volume with buffer (NaP_i, 10 mM, pH 8). Dipeptide and pentapeptide were made up to a final volume of 500 μL, while H3 K9Dha was made up to 50 μL, and eH3 K4Dha 30 μL. Time points were taken at 0, 5, 10, 15, 30, 60, 90, 120, 180, 240, 300 minutes by quenching the reaction with formic acid solution. Specifically, the dipeptide – 25 μL was added to 2.5 μL of 10% formic acid in H_2O; pentapeptide – 5 μL was diluted into 50 μL of 1% formic acid in H_2O. For both proteins 0.5 μL was diluted into 30 μL of 1% formic acid in H_2O. Analysis was carried out using a Waters Xevo G2-XS QTof mass spectrometer.

For the dipeptide **1**, 5 μL was injected and the sample analysed over a gradient of 5-80% B (0.1 formic in MeCN) over 2.5 min, 0.2 mL min^{-1} (Waters Acquity UPLC BEH C18 1.7 μm, 2.1 x 50 mm). In this case – even after significant optimisation the starting dipeptide and product lactamide fragment co-elute. The peak was summed, and the intensities of the ions were used to calculate the % of starting material remaining.

For the pentapeptide **4**, 1.5 μL was injected and the sample analysed over a gradient of 0-40% B (0.1 formic in MeCN) over 3.5 min, 0.2 mL min^{-1} (Waters Acquity UPLC BEH C18 1.7 μm, 2.1 x 50 mm). The intensities of the peaks corresponding to the starting pentapeptide and the product N-terminal amide fragment were used to calculate the % of starting material remaining.

For the proteins, 1.5 μL was injected and the sample analysed as per all other protein samples presented above. After deconvolution the intensities of the starting material and product were used to calculate the % of starting material remaining.

3.18 Antioxidative Effect of Sodium Ascorbate

To demonstrate the antioxidative effect of sodium ascorbate a number of solutions were prepared and analysed by ^{11}B NMR (Bruker AVIII HD 500). Solutions were made up in D_2O and consisted of the following:

1. $B_2(OH)_4$ (50 mM)
2. $B_2(OH)_4$ (50 mM) + H_2O_2 (10 mM)
3. $B_2(OH)_4$ (50 mM) + H_2O_2 (10 mM) + NaAsc (50 mM)
4. $B_2(OH)_4$ (50 mM) + H_2O_2 (25 mM)
5. $B_2(OH)_4$ (50 mM) + H_2O_2 (25 mM) + NaAsc (50 mM)
6. $B_2(OH)_4$ (50 mM) + H_2O_2 (50 mM)
7. $B_2(OH)_4$ (50 mM) + H_2O_2 (50 mM) + NaAsc (50 mM)

Spectra were recorded for 32 scans and the intensities of the $B_2(OH)_4$ and oxidised $B(OH)_3$ were compared.

4 NMR Spectra

S1 (^{1}H, ^{11}B – DMSO-*d6*)

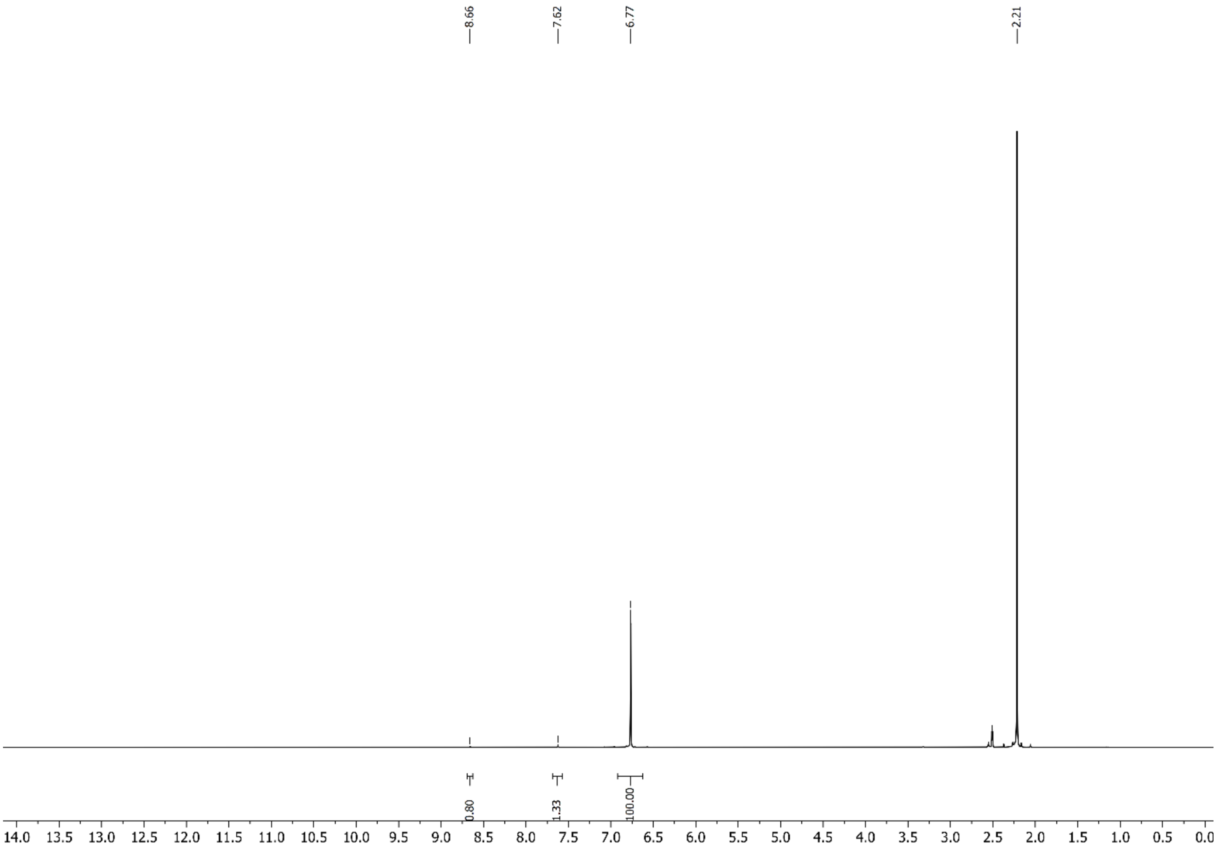

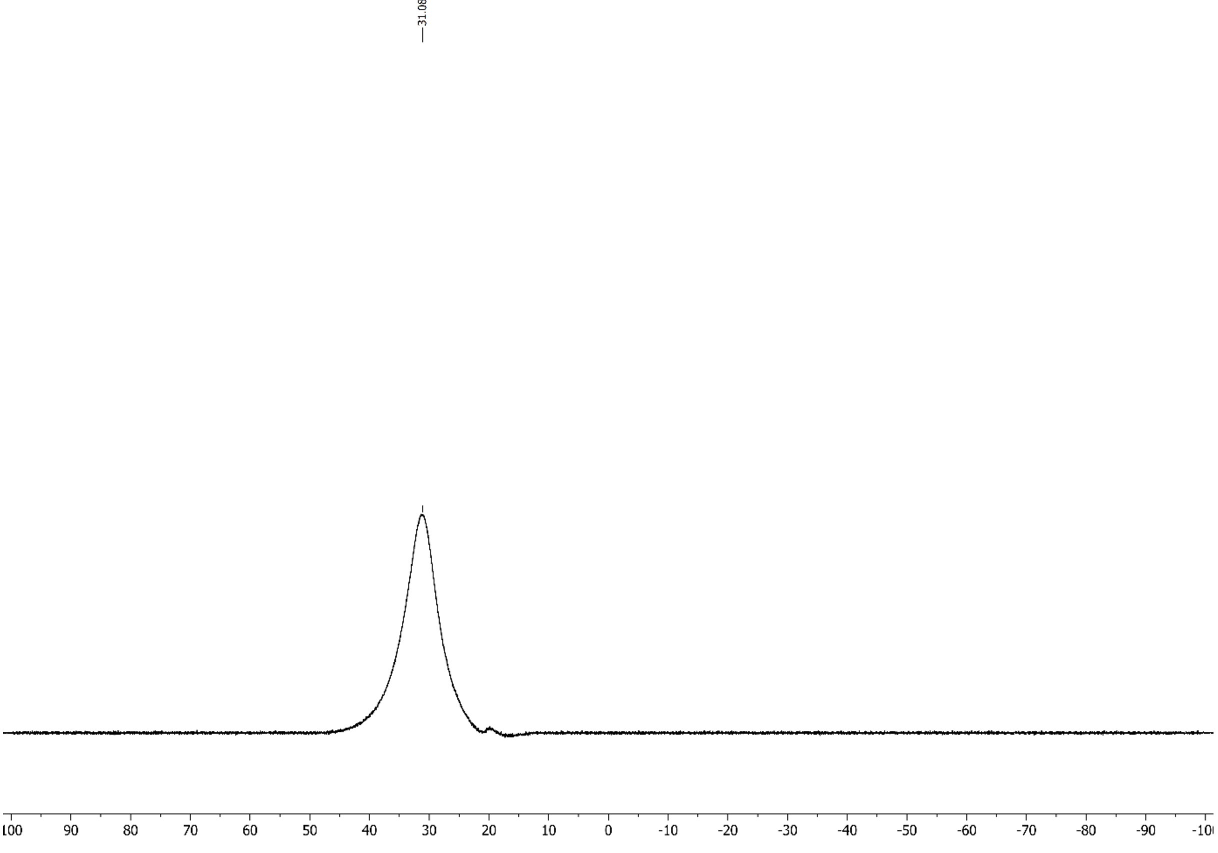

S2 (^{1}H, [13]C – DMSO-d_6)

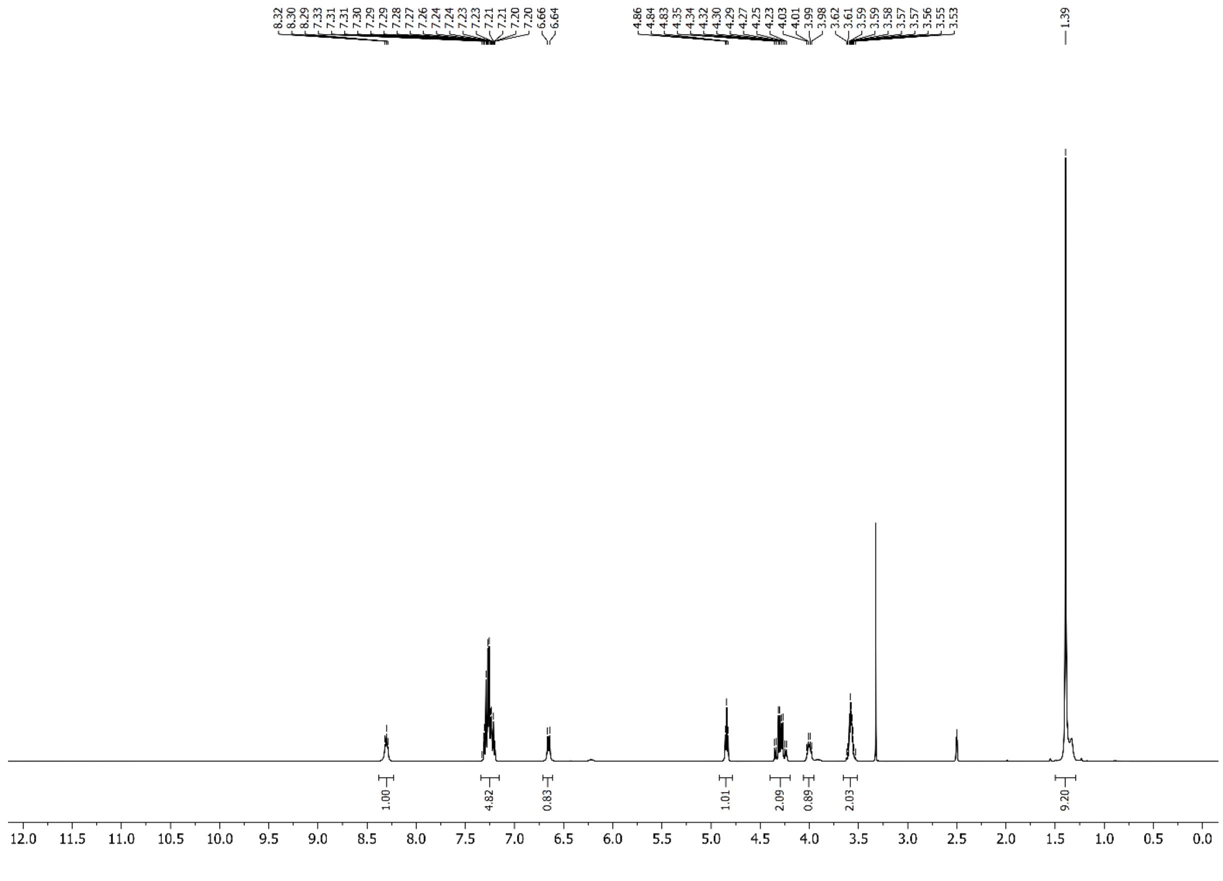

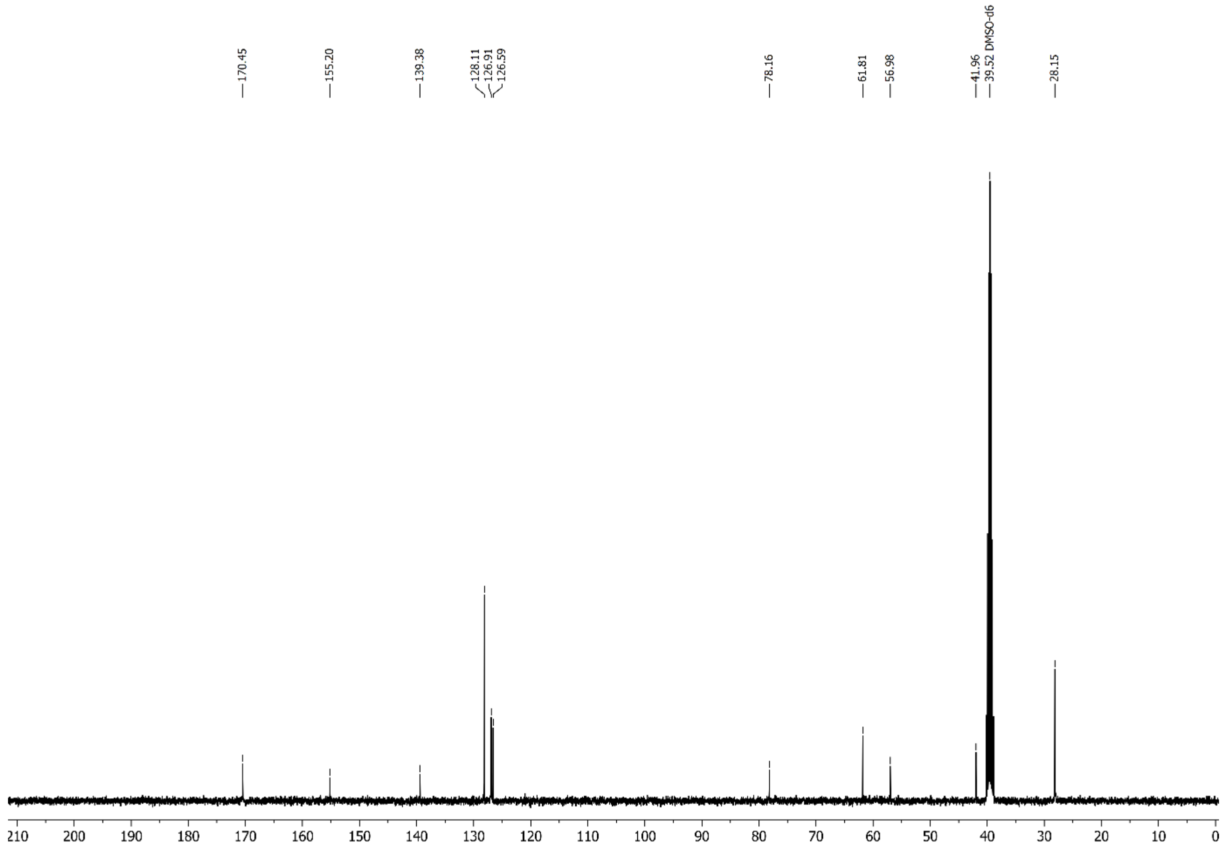

S3 (^{1}H, ^{13}C – DMSO-d_6)

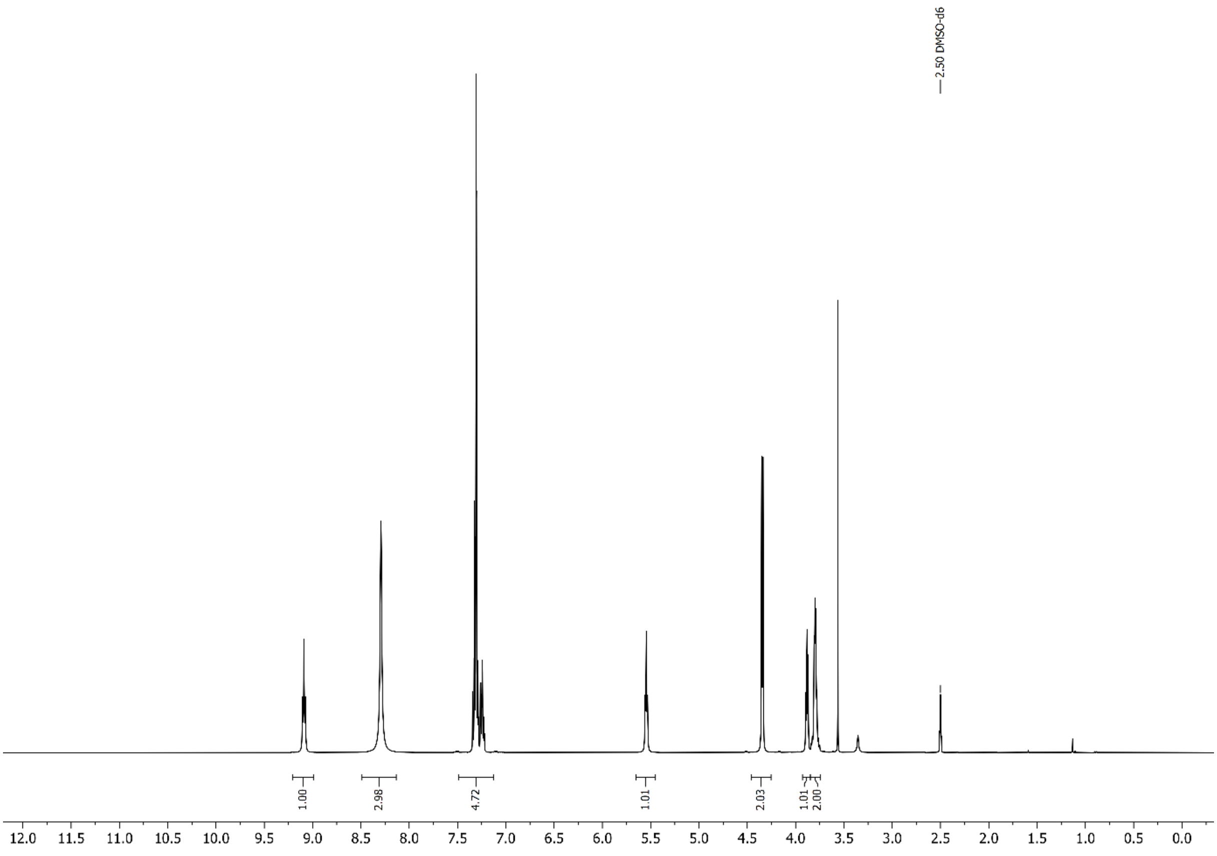

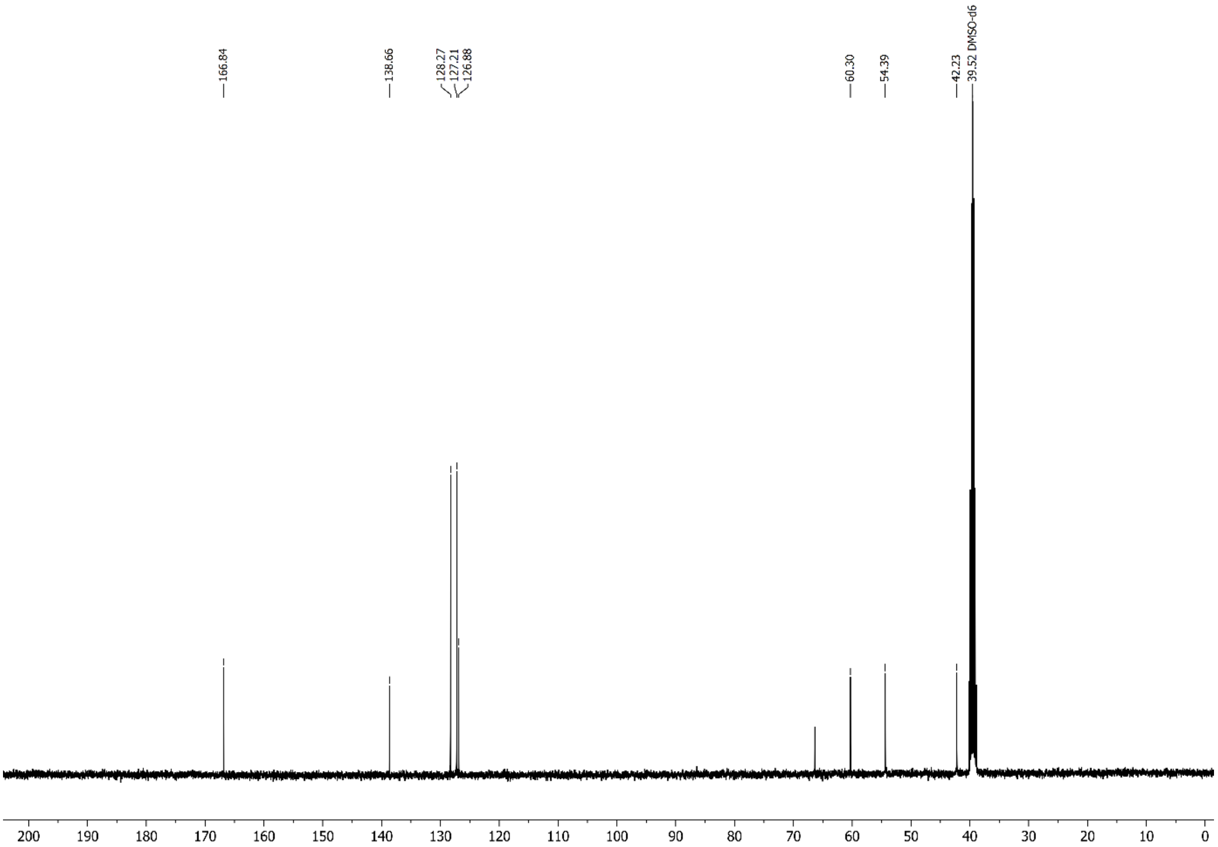

S4 (^{1}H, ^{13}C – DMSO-d_6)

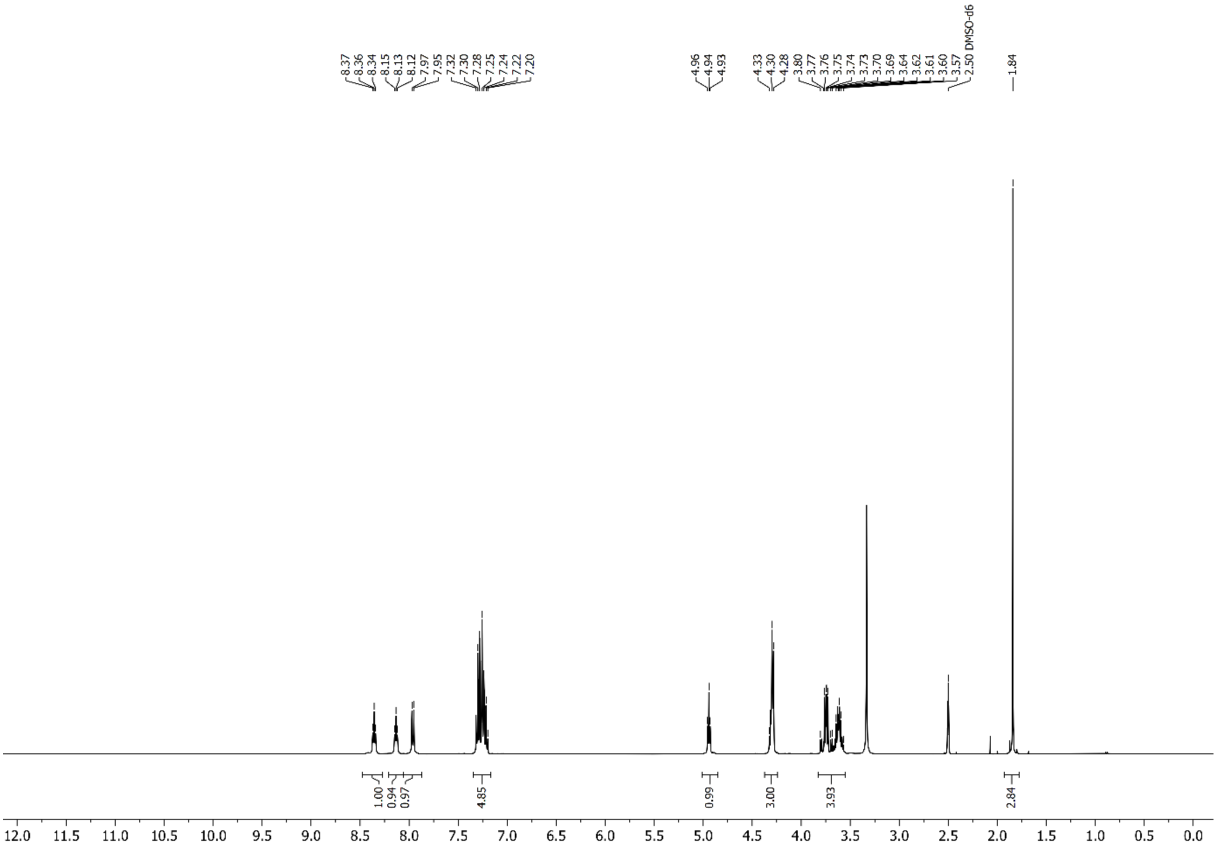

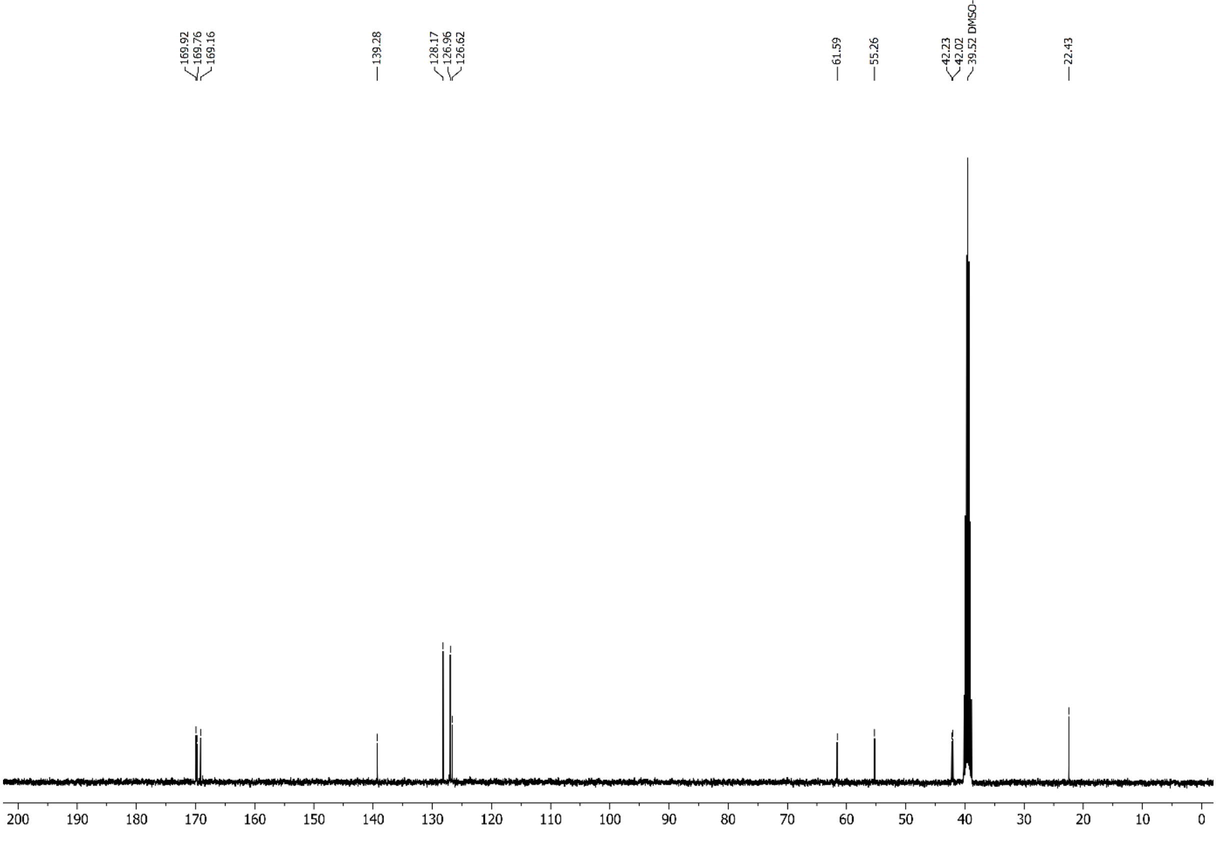

1 (^{1}H, ^{13}C – DMSO-d_6)

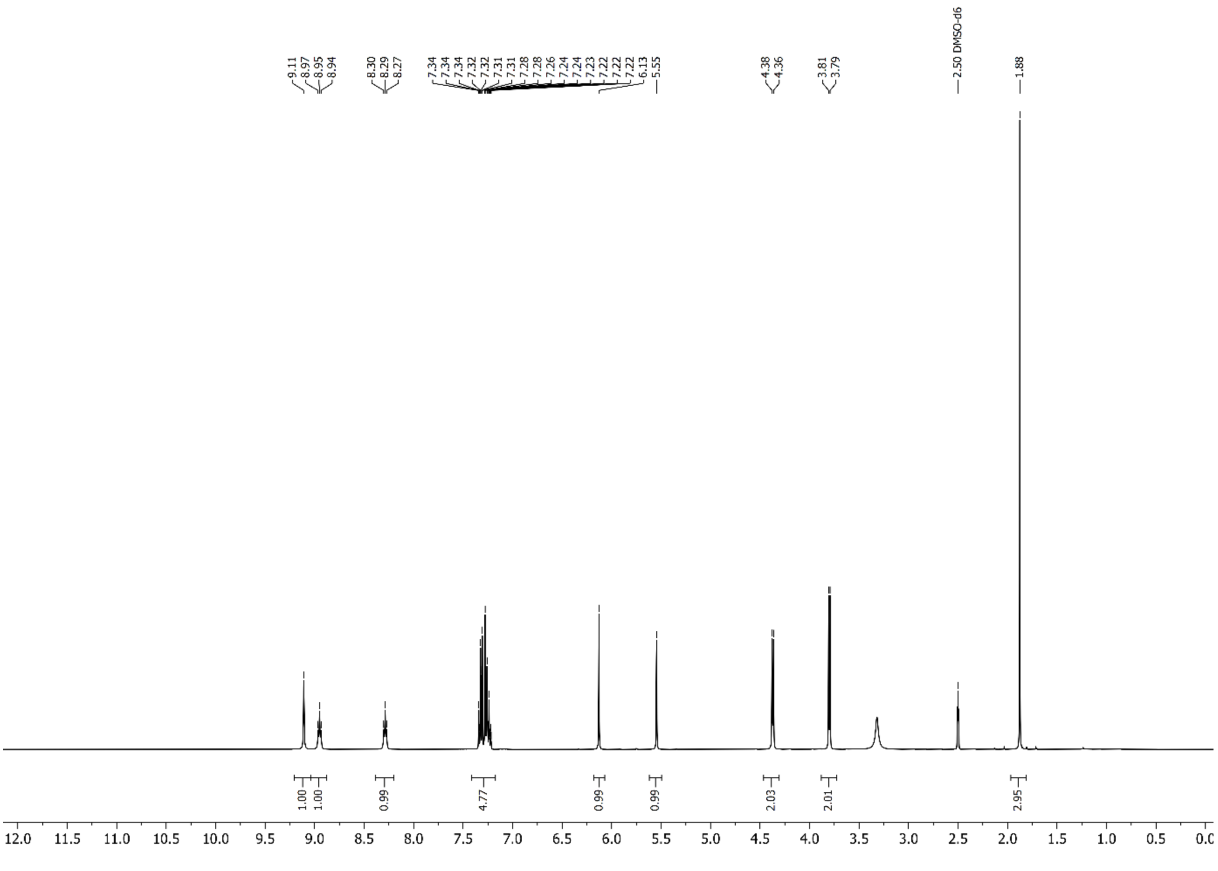

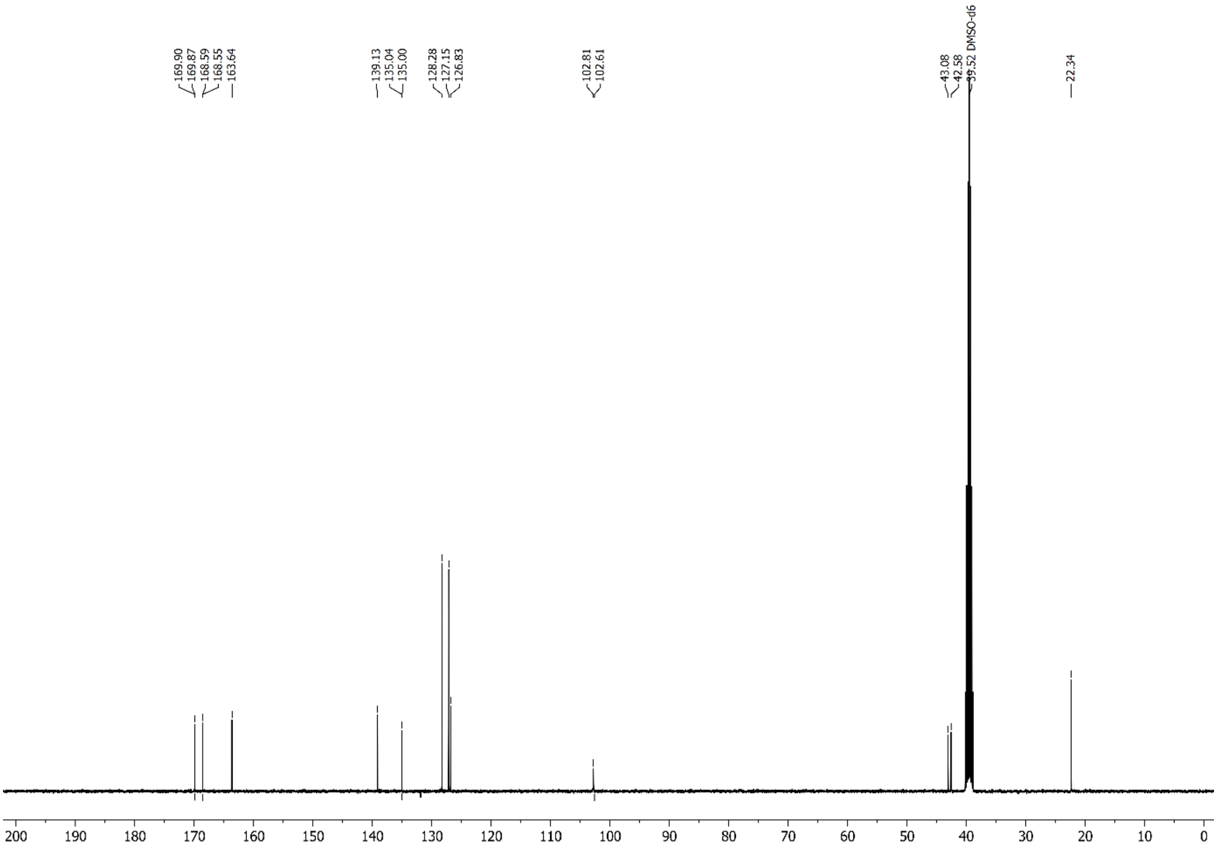

2 (^{1}H, ^{13}C – DMSO-*d6*)

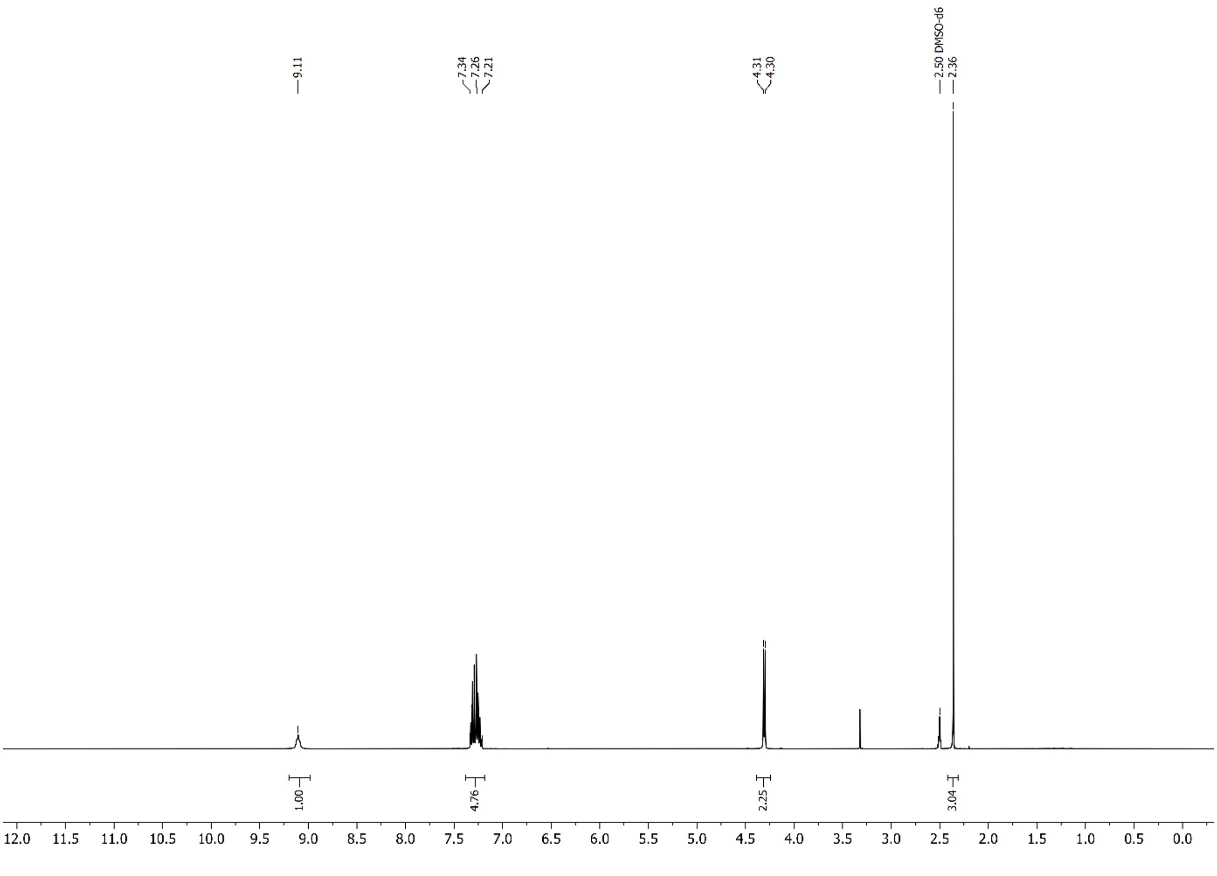

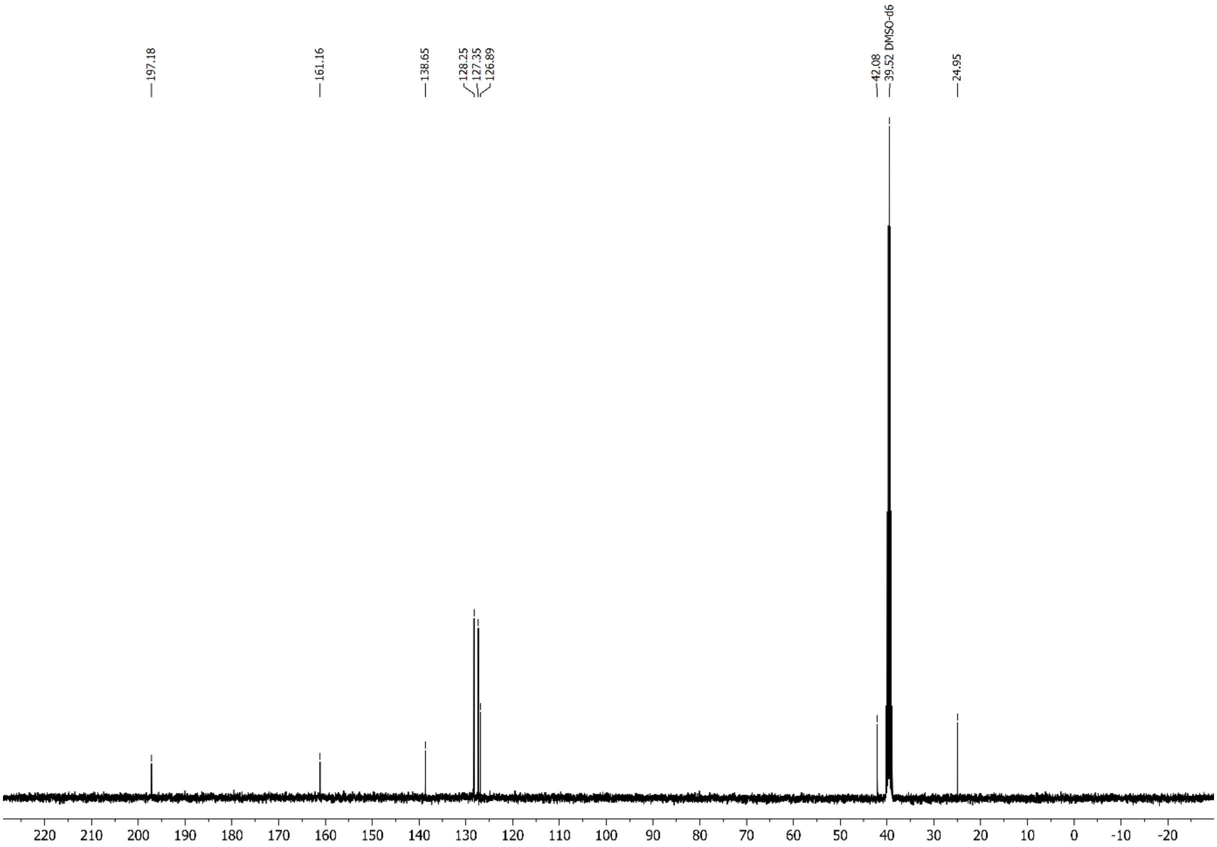

3 (^{1}H, ^{13}C – DMSO-d_6)

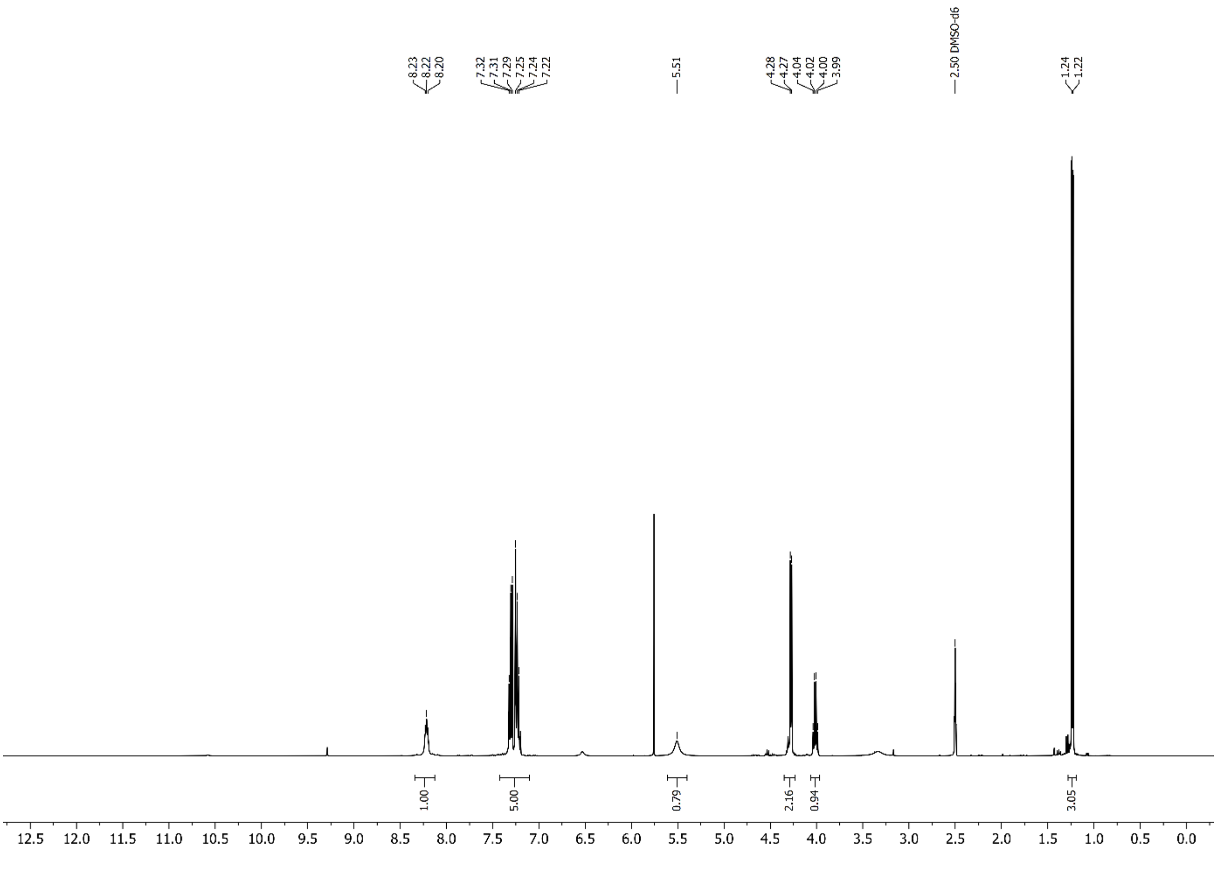

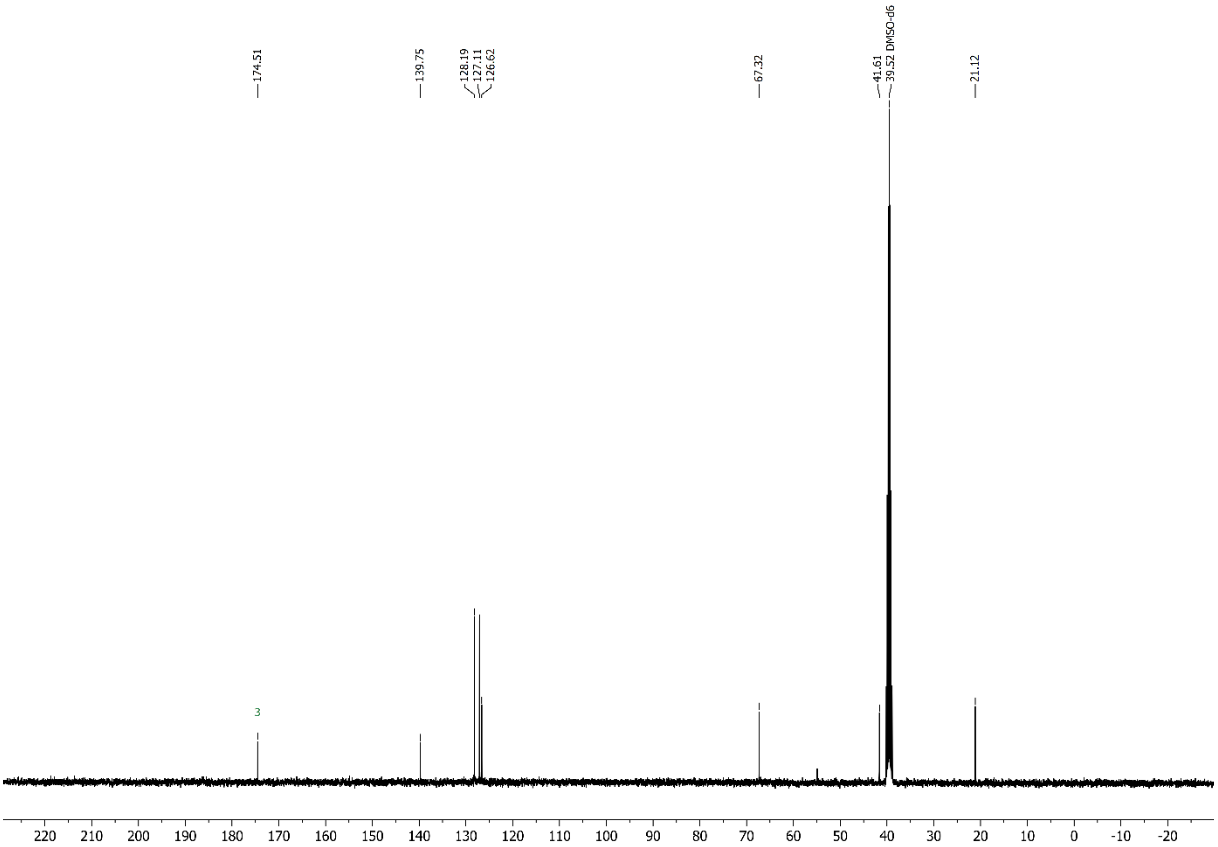

REFERENCES AND NOTES

1. E. A. Doherty, J. A. Doudna, Ribozyme structures and mechanisms. *Annu. Rev. Biophys. Biomol. Struct.* **30**, 457–475 (2001).

2. K. F. Blount, O. C. Uhlenbeck, The structure-function dilemma of the hammerhead ribozyme. *Annu. Rev. Biophys. Biomol. Struct.* **34**, 415–440 (2005).

3. S. Tsukiji, M. Miyagawa, Y. Takaoka, T. Tamura, I. Hamachi, Ligand-directed tosyl chemistry for protein labeling in vivo. *Nat. Chem. Biol.* **5**, 341–343 (2009).

4. T. Tamura, I. Hamachi, Chemistry for covalent modification of endogenous/native proteins: From test tubes to complex biological systems. *J. Am. Chem. Soc.* **141**, 2782–2799 (2019).

5. J. M. Chalker, G. J. L. Bernardes, B. G. Davis, A "tag-and-modify" approach to site-selective protein modification. *Acc. Chem. Res.* **44**, 730–741 (2011).

6. C. D. Spicer, B. G. Davis, Selective chemical protein modification. *Nat. Commun.* **5**, 4740 (2014).

7. D. B. Diaz, A. K. Yudin, The versatility of boron in biological target engagement. *Nat. Chem.* **9**, 731–742 (2017).

8. T. L. Halo, J. Appelbaum, E. M. Hobert, D. M. Balkin, A. Schepartz, Selective recognition of protein tetraserine motifs with a cell-permeable, pro-fluorescent bis-boronic acid. *J. Am. Chem. Soc.* **131**, 438–439 (2009).

9. T. A. Mollner, P. G. Isenegger, B. Josephson, C. Buchanan, L. Lercher, D. Oehlrich, D. F. Hansen, S. Mohammed, A. J. Baldwin, V. Gouverneur, B. G. Davis, Post-translational insertion of boron in proteins to probe and modulate function. *Nat. Chem. Biol.* **17**, 1245–1261 (2021).

10. E. C. Neeve, S. J. Geier, I. A. I. Mkhalid, S. A. Westcott, T. B. Marder, Diboron(4) compounds: From structural curiosity to synthetic workhorse. *Chem. Rev.* **116**, 9091–9161 (2016).

11. M. Zhou, K. Li, D. Chen, R. Xu, G. Xu, W. Tang, Enantioselective reductive coupling of imines templated by chiral diboron. *J. Am. Chem. Soc.* **142**, 10337–10342 (2020).

12. S. P. Cummings, T. Le, G. E. Fernandez, L. G. Quiambao, B. J. Stokes, Tetrahydroxydiboron-mediated palladium-catalyzed transfer hydrogenation and deuteriation of alkenes and alkynes using water as the stoichiometric H or D atom donor. *J. Am. Chem. Soc.* **138**, 6107–6110 (2016).

13. W. Ding, Q. Song, Chemoselective catalytic reduction of conjugated α,β-unsaturated ketones to saturated ketones via a hydroboration/protodeboronation strategy. *Org. Chem. Front.* **3**, 14–18 (2016).

14. K. A. Korvinson, H. K. Akula, C. T. Malinchak, D. Sebastian, W. Wei, T. A. Khandaker, M. R. Andrzejewska, B. Zajc, M. K. Lakshman, Catalytic reductions without external hydrogen gas: Broad scope hydrogenations with tetrahydroxydiboron and a tertiary amine. *Adv. Synth. Catal.* **362**, 166–176 (2020).

15. H.-C. Du, N. Simmons, J. C. Faver, Z. Yu, M. Palaniappan, K. Riehle, M. M. Matzuk, A mild, DNA-compatible nitro reduction using $B_2 (OH)_4$. *Org. Lett.* **21**, 2194–2199 (2019).

16. J. Kim, C. R. Bertozzi, A bioorthogonal reaction of N- oxide and boron reagents. *Angew. Chem.* **127**, 16003–16007 (2015).

17. Z.-Y. Sun, S. Zhou, K. Yang, M. Guo, W. Zhao, X. Tang, G. Wang, Tetrahydroxydiboron-promoted radical addition of alkynols. *Org. Lett.* **22**, 6214–6219 (2020).

18. M. Flinker, H. Yin, R. W. Juhl, E. Z. Eikeland, J. Overgaard, D. U. Nielsen, T. Skrydstrup, Efficient water reduction with sp3-sp3Diboron(4) Compounds: Application to hydrogenations, H-D exchange reactions, and carbonyl reductions. *Angew. Chem. Int. Ed.* **56**, 15910–15915 (2017).

19. T. H. Wright, B. J. Bower, J. M. Chalker, G. J. L. Bernardes, R. Wiewiora, W.-L. Ng, R. Raj, S. Faulkner, M. R.obert J. Vallée, A. Phanumartwiwath, O. D. Coleman, M.-L. Thézénas, M. Khan, S. R. G. Galan, L. Lercher, M. W. Schombs, S. Gerstberger, M. E. Palm-Espling, A. J. Baldwin, B. M. Kessler, T. D. W. Claridge, S. Mohammed, B. G. Davis, Posttranslational mutagenesis: A chemical strategy for exploring protein side-chain diversity. *Science* **354**, aag1465 (2016).

20. T. Klein, U. Eckhard, A. Dufour, N. Solis, C. M. Overall, Proteolytic cleavage - mechanisms, function, and "omic" approaches for a near-ubiquitous posttranslational modification. *Chem. Rev.* **118**, 1137–1168 (2018).

21. H. Neurath, K. A. Walsh, Role of proteolytic enzymes in biological regulation (a review). *Proc. Natl. Acad. Sci.* **73**, 3825–3832 (1976).

22. M. Vizovišek, R. Vidmar, M. Drag, M. Fonović, G. S. Salvesen, B. Turk, Protease specificity: Towards in vivo imaging applications and biomarker discovery. *Trends Biochem. Sci.* **43**, 829–844 (2018).

23. E. A. Bayer, J. Grootjans, R. Alon, M. Wilchek, Affinity cleavage and targeted catalysis of proteins using the avidin-biotin system. *Biochemistry* **29**, 11274–11279 (1990).

24. B. G. Davis, R. F. Sala, D. R. W. Hodgson, A. Ullman, K. Khumtaveeporn, D. A. Estell, K. Sanford, R. R. Bott, J. B. Jones, Selective protein degradation by ligand-targeted enzymes: Towards the creation of catalytic antagonists. *Chembiochem* **4**, 533–537 (2003).

25. I. Collins, H. Wang, J. J. Caldwell, R. Chopra, Chemical approaches to targeted protein degradation through modulation of the ubiquitin–proteasome pathway. *Biochem. J.* **474**, 1127–1147 (2017).

26. M. Schauperl, J. E. Fuchs, B. J. Waldner, R. G. Huber, C. Kramer, K. R. Liedl, Characterizing protease specificity: How many substrates do we need? *PLOS ONE* **10**, e0142658 (2015).

27. P. Roepstorff, J. Fohlman, Letter to the editors. *Biomed. Mass Spectrom.* **11**, 601–601 (1984).

28. IUPAC-IUB joint commission on biochemical nomenclature (JCBN), nomenclature and symbolism for amino acids and peptides. *Eur. J. Biochem.* **138**, 9–37 (1984).

29. G. R. Jacobson, M. H. Schaffer, G. R. Stark, T. C. Vanaman, Specific chemical cleavage in high yield at the amino peptide bonds of cysteine and cystine residues. *J. Biol. Chem.* **248**, 6583–6591 (1973).

30. A. Li, R. C. Sowder, L. E. Henderson, S. P. Moore, D. J. Garfinkel, R. J. Fisher, Chemical cleavage at aspartyl residues for protein identification. *Anal. Chem.* **73**, 5395–5402 (2001).

31. D. L. Crimmins, S. M. Mische, N. D. Denslow, Chemical cleavage of proteins in solution. *Curr. Protoc. Protein Sci.* **41**, 1–11 (2005).

32. H. E. Elashal, M. Raj, Site-selective chemical cleavage of peptide bonds. *Chem. Commun.* **52**, 6304–6307 (2016).

33. G. Li, S. Ma, M. Szostak, Amide bond activation: The power of resonance. *Trends Chem.* **2**, 914–928 (2020).

34. B. Dang, M. Mravic, H. Hu, N. Schmidt, B. Mensa, W. F. DeGrado, SNAC-tag for sequence-specific chemical protein cleavage. *Nat. Methods* **16**, 319–322 (2019).

35. Y. Seki, K. Tanabe, D. Sasaki, Y. Sohma, K. Oisaki, M. Kanai, Serine-selective aerobic cleavage of peptides and a protein using a water-soluble copper-organoradical conjugate. *Angew. Chem. Int. Ed.* **53**, 6501–6505 (2014).

36. F. B. Peters, A. Brock, J. Wang, P. G. Schultz, Photocleavage of the polypeptide backbone by 2-nitrophenylalanine. *Chem. Biol.* **16**, 148–152 (2009).

37. S. T. Prigge, A. S. Kolhekar, B. A. Eipper, R. E. Mains, L. M. Amzel, Substrate-mediated electron transfer in peptidylglycine alpha-hydroxylating monooxygenase. *Nat. Struct. Biol.* **6**, 976–983 (1999).

38. J. M. Chalker, S. B. Gunnoo, O. Boutureira, S. C. Gerstberger, M. Fernández-González, G. J. L. Bernardes, L. Griffin, H. Hailu, C. J. Schofield, B. G. Davis, Methods for converting cysteine to dehydroalanine on peptides and proteins. *Chem. Sci.* **2**, 1666–1676 (2011).

39. F. P. Seebeck, J. W. Szostak, Ribosomal synthesis of dehydroalanine-containing peptides. *J. Am. Chem. Soc.* **128**, 7150–7151 (2006).

40. J. Wang, S. M. Schiller, P. G. Schultz, A biosynthetic route to dehydroalanine-containing proteins. *Angew. Chem. Int. Ed.* **46**, 6849–6851 (2007).

41. A. Yang, S. Ha, J. Ahn, R. Kim, S. Kim, Y. Lee, J. Kim, D. Söll, H. Y. Lee, H. S. Park, A chemical biology route to site-specific authentic protein modifications. *Science* **354**, 623–626 (2016).

42. A. Patchornik, M. Sokolovsky, Nonenzymatic cleavages of peptide chains at the cysteine and serine residues through their conversion into dehydroalanine. I. Hydrolytic and oxidative cleavage of dehydroalanine residues. *J. Am. Chem. Soc.* **86**, 1206–1212 (1964).

43. T. B. Nguyen, J. Sorres, M. Q. Tran, L. Ermolenko, A. Al-Mourabit, Boric acid: A highly efficient catalyst for transamidation of carboxamides with amines. *Org. Lett.* **14**, 3202–3205 (2012).

44. J. Wang, W. Zheng, Y. Zheng, Theoretical study on homolytic B–B cleavages of diboron(4) compounds. *RSC Adv.* **7**, 49251–49272 (2017).

45. H. Steen, M. Mann, The abc's (and xyz's) of peptide sequencing. *Nat. Rev. Mol. Cell Biol.* **5**, 699–711 (2004).

46. L. Zhang, L. Jiao, Super electron donors derived from diboron. *Chem. Sci.* **9**, 2711–2722 (2018).

47. C. L. Hawkins, M. J. Davies, Generation and propagation of radical reactions on proteins. *Biochim. Biophys. Acta Bioenerg.* **1504**, 196–219 (2001).

48. A. Broisat, S. Hernot, J. Toczek, J. de Vos, L. M. Riou, S. Martin, M. Ahmadi, N. Thielens, U. Wernery, V. Caveliers, S. Muyldermans, T. Lahoutte, D. Fagret, C. Ghezzi, N. Devoogdt, Nanobodies targeting mouse/human VCAM1 for the nuclear imaging of atherosclerotic lesions. *Circ. Res.* **110**, 927–937 (2012).

49. A. A. Tokmakov, A. Kurotani, T. Takagi, M. Toyama, M. Shirouzu, Y. Fukami, S. Yokoyama, Multiple post-translational modifications affect heterologous protein synthesis. *J. Biol. Chem.* **287**, 27106–27116 (2012).

50. G. Walsh, R. Jefferis, Post-translational modifications in the context of therapeutic proteins. *Nat. Biotechnol.* **24**, 1241–1252 (2006).

51. T. Nuijens, E. Piva, J. A. W. Kruijtzer, D. T. S. Rijkers, R. M. J. Liskamp, P. J. L. M. Quaedflieg, Enzymatic C-terminal amidation of amino acids and peptides. *Tetrahedron Lett.* **53**, 3777–3779 (2012).

52. D. J. Merkler, C-Terminal amidated peptides: Production by the in vitro enzymatic amidation of glycine-extended peptides and the importance of the amide to bioactivity. *Enzym. Microb. Technol.* **16**, 450–456 (1994).

53. J. R. Gareau, C. D. Lima, The SUMO pathway: Emerging mechanisms that shape specificity, conjugation and recognition. *Nat. Rev. Mol. Cell Biol.* **11**, 861–871 (2010).

54. A. Flotho, F. Melchior, Sumoylation: A regulatory protein modification in health and disease. *Annu. Rev. Biochem.* **82**, 357–385 (2013).

55. X. Lu, S. K. Olsen, A. D. Capili, J. S. Cisar, C. D. Lima, D. S. Tan, Designed semisynthetic protein inhibitors of Ub/Ubl E1 activating enzymes. *J. Am. Chem. Soc.* **132**, 1748–1749 (2010).

56. D. R. Nässel, M. Zandawala, T. Kawada, H. Satake, Tachykinins: Neuropeptides that are ancient, diverse, widespread and functionally pleiotropic. *Front. Neurosci.* **13**, (2019).

57. R. Patacchini, L. Quartara, P. Rovero, C. Goso, C. A. Maggi, Role of C-terminal amidation on the biological activity of neurokinin A derivatives with agonist and antagonist properties. *J. Pharmacol. Exp. Ther.* **264**, 17–21 (1993).

58. D. S. Waugh, An overview of enzymatic reagents for the removal of affinity tags. *Protein Expr. Purif.* **80**, 283–293 (2011).

59. K.-H. Kim, B. L. Seong, Peptide amidation: Production of peptide hormonesin vivo andin vitro. *Biotechnol. Bioprocess Eng.* **6**, 244–251 (2001).

60. D. Mathur, S. Prakash, P. Anand, H. Kaur, P. Agrawal, A. Mehta, R. Kumar, S. Singh, G. P. S. Raghava, PEPlife: A repository of the half-life of peptides. *Sci. Rep.* **6**, 36617 (2016).

61. L. Zhang, L. Jiao, Pyridine-catalyzed radical borylation of aryl halides. *J. Am. Chem. Soc.* **139**, 607–610 (2017).

62. G. Yan, D. Huang, X. Wu, Recent advances in C-B bond formation through a free radical pathway. *Adv. Synth. Catal.* **360**, 1040–1053 (2018).

63. T. Taniguchi, Boryl radical addition to multiple bonds in organic synthesis. *Eur. J. Org. Chem.* **2019**, 6308–6319 (**2019**).

64. L. Kollipara, R. P. Zahedi, Protein carbamylation: In vivo modification or in vitro artefact? *Proteomics* **13**, 941–944 (2013).

65. C. A. Schneider, W. S. Rasband, K. W. Eliceiri, NIH image to ImageJ: 25 years of image analysis. *Nat. Methods* **9**, 671–675 (2012).

66. D. K. Wilkins, S. B. Grimshaw, V. Receveur, C. M. Dobson, J. A. Jones, L. J. Smith, Hydrodynamic radii of native and denatured proteins measured by pulse field gradient NMR techniques. *Biochemistry* **38**, 16424–16431 (1999).

67. A. J. Baldwin, L. E. Kay, NMR spectroscopy brings invisible protein states into focus. *Nat. Chem. Biol.* **5**, 808–814 (2009).

68. R. B. Kapust, J. Tözsér, T. D. Copeland, D. S. Waugh, The P1′ specificity of tobacco etch virus protease. *Biochem. Biophys. Res. Commun.* **294**, 949–955 (2002).

69. S. Nallamsetty, R. B. Kapust, J. Tözsér, S. Cherry, J. E. Tropea, T. D. Copeland, D. S. Waugh, Efficient site-specific processing of fusion proteins by tobacco vein mottling virus protease in vivo and in vitro. *Protein Expr. Purif.* **38**, 108–115 (2004).

70. E. R. LaVallie, J. M. McCoy, D. B. Smith, P. Riggs, Enzymatic and chemical cleavage of fusion proteins. *Curr. Protoc. Mol. Biol.* **28**, 5–17 (1994).

71. R. J. Jenny, K. G. Mann, R. L. Lundblad, A critical review of the methods for cleavage of fusion proteins with thrombin and factor Xa. *Protein Expr. Purif.* **31**, 1–11 (2003).

72. S. J. Wearne, Factor Xa cleavage of fusion proteins. *FEBS Lett.* **263**, 23–26 (1990).

73. V. Rahali, J. Gueguen, Chemical cleavage of bovine β-lactoglobulin by BNPS-skatole for preparative purposes: Comparative study of hydrolytic procedures and peptide characterization. *J. Protein Chem.* **18**, 1–12 (1999).

74. H. Y. Tang, D. W. Speicher, Identification of alternative products and optimization of 2-nitro-5-thiocyanatobenzoic acid cyanylation and cleavage at cysteine residues. *Anal. Biochem.* **334**, 48–61 (2004).

75. H. Zhang, M. Li, S. Shi, C. Yin, S. Jia, Z. Wang, Y. Liu, Design and identification of a high efficient formic acid cleavage site for separation of fusion protein. *Protein J.* **34**, 9–17 (2015).

76. F. Schäfer, A. Schäfer, K. Steinert, A highly specific system for efficient enzymatic removal of tags from recombinant proteins. *J. Biomol. Tech.* **13**, 158–171 (2002).

77. B. Lee, F. M. Richards, The interpretation of protein structures: Estimation of static accessibility. *J. Mol. Biol.* **55**, 379–400 (1971).

78. S. Mitternacht, FreeSASA: An open source C library for solvent accessible surface area calculations. *F1000Res.* **5**, 189 (2016).

79. B. Josephson, C. Fehl, P. G. Isenegger, S. Nadal, T. H. Wright, A. W. J. Poh, B. J. Bower, A. M. Giltrap, L. Chen, C. Batchelor-McAuley, G. Roper, O. Arisa, J. B. I. Sap, A. Kawamura, A. J. Baldwin, S. Mohammed, R. G. Compton, V. Gouverneur, B. G. Davis, Light-driven post-translational installation of reactive protein side chains. *Nature* **585**, 530–537 (2020).

80. J. Cox, M. Mann, MaxQuant enables high peptide identification rates, individualized p.p.b.-range mass accuracies and proteome-wide protein quantification. *Nat. Biotechnol.* **26**, 1367–1372 (2008).

81. A. Yang, F. Hu, Z. Li, M. Chen, J. Cai, L. Wang, T. Zhang, C. Zhao, F. Zhang, Improved synthesis and impurity identification of (R)-lacosamide. *Org. Process Res. Dev.* **23**, 818–824 (2019).

82. A. M. King, C. Salomé, J. Dinsmore, E. Salomé-Grosjean, M. de Ryck, R. Kaminski, A. Valade, H. Kohn, Primary amino acid derivatives: Compounds with anticonvulsant and neuropathic pain protection activities. *J. Med. Chem.* **54**, 4815–4830 (2011).

83. D. Ghosh, R. Nandi, S. Khamarui, S. Ghosh, D. K. Maiti, Selective amidation by a photocatalyzed umpolung reaction. *Chem. Commun.* **55**, 3883–3886 (2019).

84. M. Trincado, K. Kühlein, H. Grützmacher, Metal-ligand cooperation in the catalytic dehydrogenative coupling (DHC) of polyalcohols to carboxylic acid derivatives. *Chem. Eur. J.* **17**, 11905–11913 (2011).
